# Genomic positional conservation identifies topological anchor point RNAs linked to developmental loci

**DOI:** 10.1186/s13059-018-1405-5

**Published:** 2018-03-15

**Authors:** Paulo P. Amaral, Tommaso Leonardi, Namshik Han, Emmanuelle Viré, Dennis K. Gascoigne, Raúl Arias-Carrasco, Magdalena Büscher, Luca Pandolfini, Anda Zhang, Stefano Pluchino, Vinicius Maracaja-Coutinho, Helder I. Nakaya, Martin Hemberg, Ramin Shiekhattar, Anton J. Enright, Tony Kouzarides

**Affiliations:** 1 The Gurdon Institute, University of Cambridge, Tennis Court Road, Cambridge, CB2 1QN UK; 2 European Molecular Biology Laboratory, European Bioinformatics Institute (EMBL-EBI), Wellcome Genome Campus, Hinxton, Cambridge, CB10 1SD UK; 3 Department of Clinical Neurosciences and NIHR Biomedical Research Centre, University of Cambridge, Cambridge, UK; 4 Centro de Genómica y Bioinformática, Facultad de Ciencias, Universidad Mayor, Santiago, Chile; 5 University of Miami Miller School of Medicine, Sylvester Comprehensive Cancer Center, Department of Human Genetics, Biomedical Research Building, Miami, FL 33136 USA; 6 School of Pharmaceutical Sciences, University of São Paulo, Av. Prof. Lineu Prestes 580, São Paulo, 05508 Brazil; 7 Wellcome Trust Sanger Institute, Wellcome Trust Genome Campus, Hinxton, CB10 1SA UK; 8 Department of Pathology, University of Cambridge, Tennis Court Road, Cambridge, CB2 1QP UK; 9 Present address: MRC Prion Unit, UCL Institute of Neurology, Queen Square House, Queen Square, London, WC1N 3BG UK; 10 Advanced Center for Chronic Diseases (ACCDiS), Facultad de Ciencias Químicas y Farmacéuticas, Universidad de Chile, Santiago, Chile; 11 Present address: The Milner Therapeutics Institute, University of Cambridge, Tennis Court Road, Cambridge, CB2 1QN UK

**Keywords:** lncRNAs, Development, Chromatin architecture, Topology, Zinc finger, Cancer

## Abstract

**Background:**

The mammalian genome is transcribed into large numbers of long noncoding RNAs (lncRNAs), but the definition of functional lncRNA groups has proven difficult, partly due to their low sequence conservation and lack of identified shared properties. Here we consider promoter conservation and positional conservation as indicators of functional commonality.

**Results:**

We identify 665 conserved lncRNA promoters in mouse and human that are preserved in genomic position relative to orthologous coding genes. These positionally conserved lncRNA genes are primarily associated with developmental transcription factor loci with which they are coexpressed in a tissue-specific manner. Over half of positionally conserved RNAs in this set are linked to chromatin organization structures, overlapping binding sites for the CTCF chromatin organiser and located at chromatin loop anchor points and borders of topologically associating domains (TADs). We define these RNAs as topological anchor point RNAs (tapRNAs). Characterization of these noncoding RNAs and their associated coding genes shows that they are functionally connected: they regulate each other’s expression and influence the metastatic phenotype of cancer cells *in vitro* in a similar fashion. Furthermore, we find that tapRNAs contain conserved sequence domains that are enriched in motifs for zinc finger domain-containing RNA-binding proteins and transcription factors, whose binding sites are found mutated in cancers.

**Conclusions:**

This work leverages positional conservation to identify lncRNAs with potential importance in genome organization, development and disease. The evidence that many developmental transcription factors are physically and functionally connected to lncRNAs represents an exciting stepping-stone to further our understanding of genome regulation.

**Electronic supplementary material:**

The online version of this article (10.1186/s13059-018-1405-5) contains supplementary material, which is available to authorized users.

## Background

Long noncoding RNAs (lncRNAs) comprise the main transcriptional output of the mammalian genome, with recent surveys cataloguing over 100,000 lncRNA genes in humans [1, 2]. While many of these lncRNAs are transcribed by RNA polymerase II and are predominantly spliced and polyadenylated in a similar manner to protein-coding genes, no sequence or structural features have been identified yet that are predictive of their biological functions.

The functions of only a small fraction of lncRNAs have been experimentally tested. A recent screen using transcriptional CRISPR interference knock-down showed that expression of hundreds of lncRNAs is essential for cellular growth, with them playing highly cell type-specific roles [3]. While lncRNA transcription can influence the expression of neighbouring genes [4], a number of studies have shown that lncRNAs themselves have diverse roles regulating genome function and gene expression at different levels [5–8]. From a mechanistic point of view, lncRNAs can act both post-transcriptionally and at the level of chromatin organization, structure and transcription, where they can affect genes in the immediate genomic vicinity (*in cis*) and/or in other genomic locations (*in trans*) to repress or promote expression. Well-studied repressors include lncRNAs associated with imprinted loci such as *AIRN* (*Antisense IGF2R ncRNA*) and *KCNQ1OT1* (*KCNQ1 opposite strand/antisense transcript 1*), which promote silencing of genomically associated genes in an allele-specific manner [9]. Examples of activator RNA loci include the lncRNAs *HOTTIP* (*HOXA transcript at the distal tip*) and transcripts with ‘enhancer-like function’, such as *ncRNA-a*, which promote expression of neighbouring genes [10–14].

In contrast to coding genes, most lncRNAs do not exhibit high levels of primary sequence conservation across species [1, 15, 16]. In fact, the increasing catalogue of characterised lncRNAs, such as *AIRN* and *XIST* (*X inactive-specific transcript*), indicates that they evolve under different functional constraints and exhibit higher evolutionary plasticity [17, 18]. Other indications as to what some of these differing constraints may be include the early observation that lncRNAs often have promoters that exhibit higher conservation and that this conservation extends to longer sequence stretches than observed for promoters of coding genes [15].

lncRNAs with conserved promoters and expressed in different species have been reported to be associated with orthologous genes that often have developmental functions [5, 19–23]. For example, the lncRNA *SOX2OT* (*SOX2 overlapping transcript*) has alternative syntenic isoforms transcribed from highly conserved promoters in all vertebrate groups, with similar expression patterns, particularly in the central nervous system [24]. Importantly, other characterised lncRNAs that fall in the same category, such as *HOTTIP, NeST/INFG-AS1* and *Evf2/Dlx6os* (*Dlx6 opposite strand transcript*), and many imprinted lncRNAs were demonstrated to participate in the regulation of the associated genes [12, 25–28]. Moreover, recent transcriptomic and cross-species analyses have shown that synteny is observed for hundreds of lncRNAs across the genomes of amniotes and beyond [16, 29]. This suggests a functional association that has been maintained across evolution, although the functional implications and regulatory features of this genomic organization are still not well understood.

Here, we systematically characterise genomic positional conservation of lncRNAs in mammals and investigate whether this feature is indicative of their biological roles. We identify 1700 positionally conserved lncRNAs, transcribed from 665 conserved syntenic promoters, and find that they are predominantly associated with developmental genes, with which they are generally co-expressed in a conserved tissue-specific manner. Our analysis identifies a new subgroup of lncRNAs, which are positioned at topological anchor points (loop end points and chromatin boundaries). These RNAs, which we call topological anchor point (tap)RNAs, have conserved domains and motifs, can regulate the expression of associated genes and similarly affect cancer-related phenotypes. This analysis provides a rich resource for the in-depth characterization of the heterogeneous family of lncRNAs and their relationship to chromatin topological organization.

## Results

### A promoter-centric approach identifies positionally conserved RNAs

There is a paucity of information regarding the classification of lncRNAs into groups with common functionality. Here we considered that the conserved position of lncRNAs relative to coding genes may define a basis for identifying and characterizing common properties and for functional indexing. We therefore set out to identify spliced lncRNAs that are positionally conserved in mammalian genomes. We compiled a comprehensive catalogue of human and mouse transcripts based on (1) Gencode annotation [30, 31]; (2) human and mouse RNA-sequencing (RNA-Seq) data from six matched tissues (brain, cerebellum, heart, kidney, liver and testis) [32]; and (3) RNA-Seq data from four similar human and mouse cell lines (embryonic stem (ES), leukaemia, lymphoblast and muscle cells) produced by the ENCODE project (Additional file 1: Table S1) [33, 34]. In total, we processed 80 RNA-Seq datasets and successfully mapped 2.6 billion reads.

**Fig. 1:**
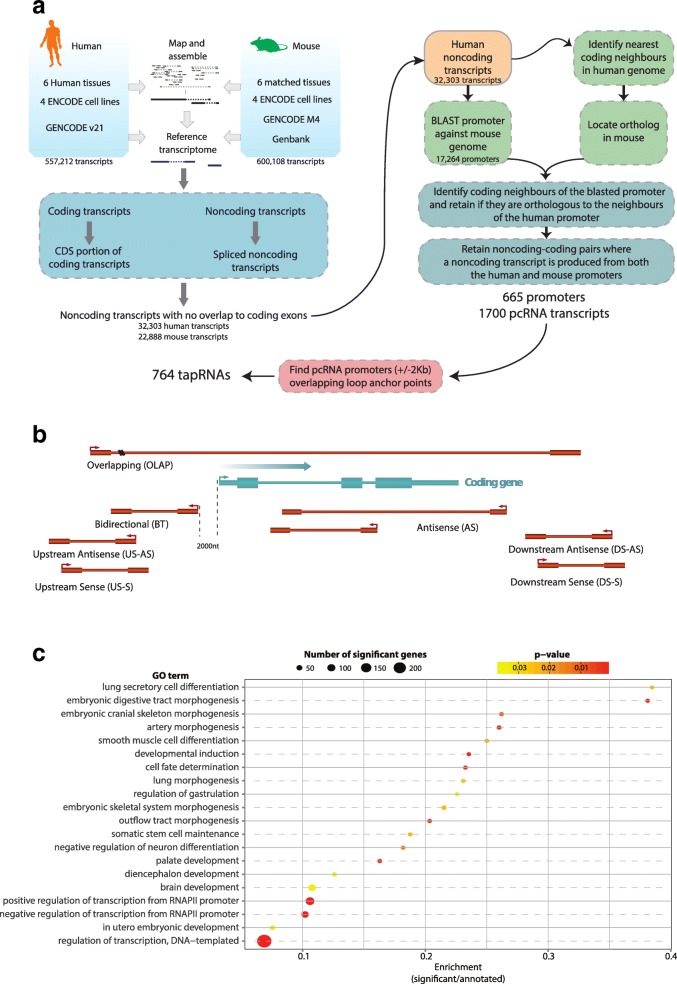
Identification of pcRNAs and tapRNAs. **a** Workflow used for the identification of pcRNAs and tapRNAs. **b** The possible orientations of a pcRNA (*red*) relative to a coding gene (*blue*). **c** Gene Ontology (*GO*) enrichment analysis of pcRNA-associated coding genes. The *x-axis* shows the enrichment score, calculated as the number of pcRNA-associated genes in a given GO category divided by the total number of genes in the category. The size of the *points* indicates the absolute number of pcRNA-associated genes in the given GO category. The colour-coding indicates the adjusted *p* value. *CDS* coding sequence

Our analysis pipeline is designed for comprehensive identification of human and mouse transcripts from both Gencode and the RNA-Seq data, with evidence of splicing, no overlap with coding exons in the same transcriptional orientation and no significant coding potential (Fig. 1a). Promoter sequences of human lncRNAs were then aligned to the mouse genome to identify syntenic lncRNAs (Additional file 2: Supplementary methods) with high promoter conservation. Syntenic lncRNAs were defined as positionally conserved if their promoters were genomically associated with orthologous genes and produced spliced lncRNAs in the same relative transcriptional orientation (either sense or antisense relative to the coding gene) in both mouse and human (Additional file 2: Supplementary methods).

The resulting set of positionally conserved lncRNAs (pcRNAs) comprises 1700 transcripts, including splicing isoforms, associated with 665 unique conserved promoters and a total of 626 orthologous coding genes (Additional file 3: Table S2). The majority of pcRNAs (82 %, 1401/1700 transcripts, transcribed from 595 independent promoters) were Gencode annotated human transcripts, while 299 (18 %) represented novel transcripts assembled from the RNA-Seq data. We also found that a small number of pcRNAs (138, transcribed from 32 independent promoters) overlapped syntenic microRNA (miRNA) loci.

We classified pcRNAs into seven categories based on their genomic orientations relative to the associated coding genes (Fig. 1b).

The predominant category is bidirectional transcripts (42 % of all pcRNAs), followed by antisense (18 %), with all other categories similarly represented (between 5 and 9 %) (Additional file 4: Figure S1a). On average they are 1.3 kb long (Additional file 4: Figure S1b), have three to four exons (mean 3.6 exons per pcRNA; Additional file 4: Figure S1c) and are proximal to protein-coding genes (Additional file 4: Figure S1d). Analysis of FANTOM5 data [35] revealed that the majority of human pcRNAs have transcription start sites (TSSs) supported by CAGE tags (9 bp median distance between TSS and closest CAGE tag; Additional file 4: Figure S2a), providing further evidence for our identification of pcRNA promoters. Despite being less conserved than their associated coding genes, human pcRNAs have on average 31 % sequence identity with their mouse counterparts (Additional file 4: Figure S2b) and display conservation at the intron–exon junctions (Additional file 4: Figure S2c). Furthermore, inspection of the lncATLAS database [36], which provides scores for expression of lncRNAs in subcellular compartments, showed that pcRNAs are predominantly localised in the nucleus (Additional file 4: Figure S2d).

### Genomic association and conserved co-regulation with genes encoding developmental transcription factors

Gene Ontology (GO) analysis of the 626 coding genes with which pcRNAs are associated showed a very strong enrichment for genes with roles in *regulation of transcription from RNA polymerase II promoter* (GO:0045944 and GO:0000122, adjusted *p* values = 1.2 × 10^−15^ and 8.5 × 10^−10^ respectively; Fig. 1c; Additional file 5: Table S3). These are transcription factors involved in *cell fate determination* (GO:0001709, adjusted *p* value = 0.00265) and *developmental induction* (GO:0031128, adjusted *p* value = 0.00265), which are central in the determination of a variety of specific developmental systems and embryonic stages, such as regulation of gastrulation, stem cell maintenance and organ morphogenesis (Additional file 5: Table S3). Notably, many of these genes belong to major gene families containing regulators of lineage specification, such as *SOX* genes (including *SOX1*, *2, 4, 9* and *21*), *FOX* genes (*FOXA2*, *D3*, *E3*, *F1*, *I* and *P4*), *HOX* genes (e.g. *HOXA1*, *A2*, *A3*, *A11*, *A13, B3, C5* and *D8*) and other homeodomain genes, as well as several nuclear receptors, such as *NR2E1*, *NR2F1* and *NR2F2* (Additional file 5: Table S3 and Additional file 6: Table S4). To verify whether the enrichment observed for pcRNAs could indirectly result from the preferential location of developmental genes in gene-sparse regions, we repeated the GO enrichment analysis controlling for the size of the intergenic region surrounding pcRNA-associated coding genes and confirmed a significant enrichment for developmental terms (Additional file 2: Supplementary methods and Additional file 4: Figure S2e).

To quantify the expression of pcRNAs and their associated protein-coding genes, we used publicly available RNA-Seq data (Additional file 1: Table S1), as well as a custom code set for the Nanostring expression assay that probed approximately 50 human and mouse manually selected pcRNAs and associated orthologous protein-coding genes (Additional file 2: Supplementary methods) across a broad panel of RNA from human and mouse tissues and cell lines (Additional file 7: Table S5).

**Fig. 2:**
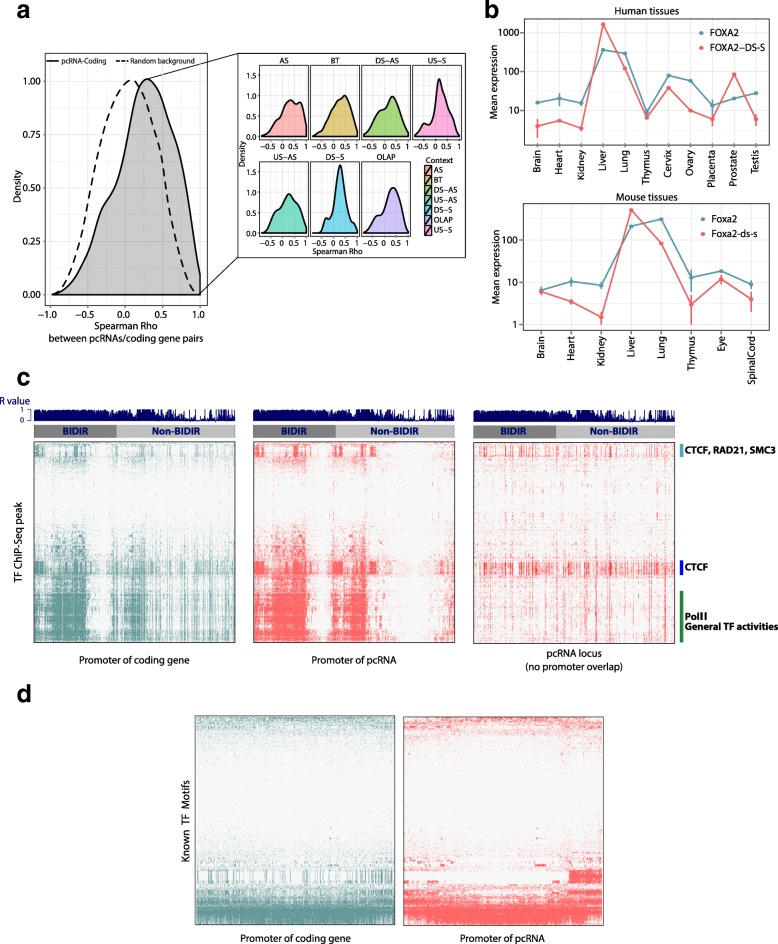
pcRNA expression and regulation. **a** Density distribution of the Spearman’s correlation coefficients between pcRNAs and corresponding coding genes in human tissues and cell lines (mean Spearman’s rho 0.25, permutation test *p* value < 10^−6^). The *dotted line* shows the background distribution of all pairwise Spearman’s correlations between pcRNAs and pcRNA-associated coding genes. *Inset*: Distributions of the Spearman’s correlation coefficients divided by the positional category of the pcRNA. *AS* antisense, *BT* bidirectional, *DS-AS* downstream antisense, *DS-S* downstream sense, *OLAP* overlapping, *US-AS* upstream antisense, *US-S* upstream sense. **b** Nanostring expression profiles of *FOXA2* and *FOXA-DS-S* across human (*top*) and mouse (*bottom*) tissues. The *points* indicate the mean value of two technical replicates, while the *vertical bars* report the value of each replicate. **c** Transcription factor binding patterns in the promoters of pcRNAs (*middle*), their associated coding genes (*left*), and across pcRNA loci (*right*). The heatmaps present the distribution of experimentally validated TF-binding sites from 2216 ENCODE ChIP-Seq experiments (*y-axis*), showing a high degree of co-occupancy between the promoters of pcRNAs (*x-axis*) and their associated coding genes. The blue bar graph on top of each heatmap shows correlation (r values) between a pcRNA and its associate coding gene. The color bars next to the right heatmap indicate the TF groups showing dominant binding patterns. **d** Same as in **c** but indicating the presence of TF-binding motifs based on known motifs annotated in JASPAR (freeze 2014–12–10, 263 motifs), in Kheradpour and Kellis [100] (2065 motifs) and in Jolma et al. [101] (843 motifs)

Both RNA-Seq and Nanostring data showed that pcRNAs have significantly conserved expression patterns across mouse and human tissues (mean Spearman’s correlation 0.26, *p* value < 10^−6^; Additional file 4: Figure S3a–d) and their expression is positively correlated with the associated coding genes (mean Spearman’s correlation 0.25, *p* value < 10^−6^; Fig. 2a; Additional file 4: Figure S3e). Furthermore, the distance between a pcRNA and the corresponding coding gene has a negligible effect on their correlation of expression, indicating that co-expression is not merely a consequence of their proximity (R^2^ = 0.008, *p* value = 3.23 × 10^−4^; Additional file 4: Figure S3f).

We found that pcRNAs display a tissue-specific expression profile and have significantly higher tissue specificity than their associated coding genes (Additional file 4: Figure S4a–d), also when correcting for their lower expression level (Additional file 4: Figure S4e, f). Tissue-specific pcRNAs tend to be genomically associated with coding genes involved in developmental and differentiation processes relevant to that particular tissue, such as neural differentiation genes for brain-specific pcRNAs and endoderm developmental genes for liver-specific pcRNAs (Additional file 4: Figure S5a–d). We validated the tissue expression and temporal co-induction for a number of coding–non-coding pairs by qPCR (Additional file 4: Figure S6). Taken together, these data suggest that pcRNAs and their corresponding coding genes are often co-regulated in mouse and human.

The Nanostring data also revealed that pcRNAs often form clusters of co-expression with several functionally related regulatory genes (Additional file 4: Figure S7a, b). For instance, two connected clusters are comprised of transcription factors that are master regulators of endoderm—in particular liver cell differentiation (*HNF1A*, *FOXA2*/*HNF3B* and *HNF4A*) [37, 38]—and associated pcRNAs. For example, *FOXA2* and its associated pcRNA *FOXA2-DS-S* exhibit very similar expression profiles across tissues in both human and mouse (Fig. 2b), and similar results are observed for *HNF1A* and its pcRNA *HNF1A-BT1/2* (Additional file 4: Figure S8a). These expression data suggest that pcRNAs share upstream regulatory elements with neighbouring protein-coding genes and/or have a role in their regulation.

### Identification of tapRNAs

To investigate the principles of pcRNA regulation, we first inspected chromatin modification profiles around their TSSs. Using the ENCODE genome-wide datasets for different human cell lines (ENCODE tier 1 lines GM12878, H1-hESCs, HSMM and K562) [33, 39], we detected a clear enrichment in H3K4 di- and tri-methylation (H3K4me2/3) and H3K9 and H3K27 acetylation (H3K9ac, H3K27ac) (Additional file 4: Figure S9), as well as H3K27 tri-methylation (H3K27me3) and bivalent marks specifically for pcRNA promoters in embryonic stem (ES) cells (Additional file 4: Figure S10). These profiles are indicative of RNA polymerase II promoters, similar to those observed for protein-coding genes (Additional file 4: Figure S9). Interrogation of the FANTOM5 Consortium database [35], containing comprehensive annotations of over 40,000 enhancer regions, identified the promoters of only three pcRNAs as enhancers (associated with *GATA2*, *HES1* and *KLF4*; data not shown).

**Fig. 3:**
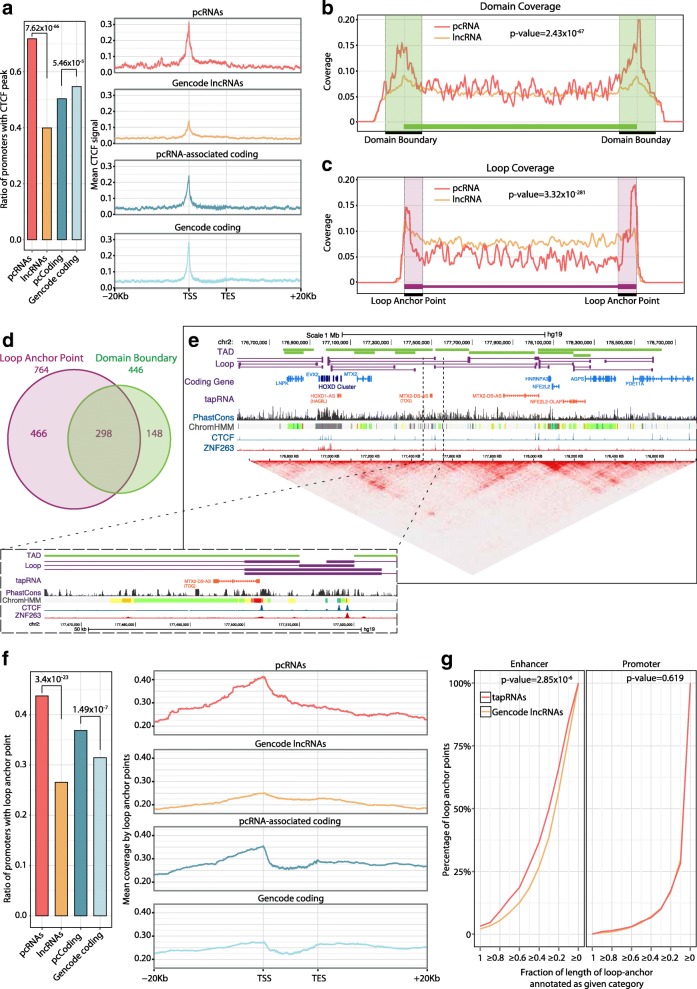
Identification of tapRNAs. **a** The proportion of pcRNAs, pcRNA-associated coding genes, Gencode lncRNAs and Gencode coding genes with a CTCF peak (based on Encode ChIP-Seq data) overlapping their promoter. The *p* values reported were calculated with hypergeometric tests. *Right*: CTCF peak coverage of loci of pcRNAs, pcRNA-associated coding genes, Gencode lncRNAs and Gencode coding genes. The plots report the loci from 20 kb upstream of the transcription start site (*TSS*) to 20 kb downstream of the transcription end site (*TES*). For visualization purposes these profiles show the coverage of a random sample of 5000 Gencode lncRNAs and 5000 random Gencode coding genes. **b, c** Aggregation density plots showing the distribution of the TSS of pcRNAs (*red*) and lncRNAs (*orange*) relative to chromatin topological domains (**b**) and chromatin loop anchor points (**c**). Domains and loop anchor points were defined based on HiC data. **d** Venn diagram showing the number of pcRNAs whose promoters overlap a loop anchor point (*purple*) or a domain boundary (*green*). **e** The *HOXD* locus showing the tapRNAs and chromatin loops defined by HiC data [43]. Modified from a screenshot of the UCSC genome browser. **f** The proportion of pcRNAs, pcRNA-associated coding genes, Gencode lncRNAs and Gencode coding genes with a HiC loop overlapping their promoter. The *p* values reported were calculated with hypergeometric tests. *Right*: HiC loop coverage of loci of pcRNAs, pcRNA-associated coding genes, Gencode lncRNAs and Gencode coding genes. The plotted genomic regions encompass the loci from 20 kb upstream of the TSS to 20 kb downstream of the TES. For visualization purposes these profiles show the coverage of a random sample of 5000 Gencode lncRNAs and 5000 random Gencode coding genes. **g** Cumulative distribution plot showing the percentage of distal genomic regions in contact with pcRNA promoters (*y-axis*) as a function of the fraction of length of loop-end annotated as enhancer (*left*) or promoter (*right*). For example, the “*≥ 0.4*” point (*x-axis*) of the *red line* in the first plot indicates that ~ 37 % (*y-axis*) of the distal genomic regions in contact with pcRNA promoters are annotated as enhancer for 40 % or more of their length. Promoters of pcRNAs are significantly more often in contact through loops with enhancer elements compared to generic Gencode lncRNAs (*p* value 2.85 × 10^−6^). The indicated *p* values were calculated using the Kolmogorov-Smirnov test

Using chromatin immunoprecipitation sequencing (ChIP-Seq) peaks for transcription factors obtained by the ENCODE project [33, 40], we observed a highly concordant pattern of occupancy in the promoters of pcRNAs and associated genes (Pearson correlation coefficient = 0.67, *p* value < 1 × 10^−3^; Fig. 2c). Such general co-regulation was further supported by the analysis of predicted transcription factor binding motifs in both promoters (Pearson correlation coefficient = 0.63, *p* value < 1 × 10^−3^; Fig. 2d). Surprisingly, we found evidence of binding for CCCTC-binding factor (CTCF) throughout pcRNA loci (Fig. 2c), but strongly enriched in the regions adjacent to the TSSs of most pcRNAs: 72 % of pcRNA promoters contain a CTCF peak and a significant enrichment is seen with respect to other spliced lncRNAs and protein-coding genes (Fig. 3a).

Since topological organization of chromatin is dictated to a large extent by CTCF binding [41, 42], we interrogated high resolution, genome-wide topological maps [43] to establish the positioning of pcRNAs and their coding genes relative to genomic loops. We found that pcRNAs are preferentially located at the boundaries of topologically associating domains (TADs) and chromatin loops (Fig. 3b, c). In particular, we noticed that a remarkable proportion of pcRNAs (912 out of 1700 pcRNAs isoforms, 54 %) have a promoter within 10 kb of a TAD boundary (446 pcRNAs) and/or directly intersecting a loop anchor point (764 pcRNAs; Fig. 3d). For example, the *HOXD* cluster is embedded in a region with high density of CTCF peaks, located at the edge of TADs with different syntenic lncRNAs in their boundaries, including *Hog* and *Tog* (*Hotdog* and *Twin-of-hotdog*) [44] (Fig. 3e). Similarly, the pcRNA *TBX2-BT* and other pcRNAs associated with important developmental genes lie at TAD boundaries and overlap multiple loop anchor points (Additional file 4: Figure S11).

The proportion of pcRNA promoters that overlap a loop anchor point or a TAD boundary is significantly higher than that of Gencode spliced lncRNAs (*p* value = 3.4 × 10^−23^; Fig. 3f; Additional file 4: Figure S12a–c). In addition, the promoters of pcRNA-associated protein-coding genes are enriched in TAD boundaries or loop anchor points compared to other coding genes, although to a lesser extent than pcRNAs themselves (Fig. 3f; Additional file 4: Figure S12a, b).

Interestingly, we found that the location where loop anchor points are positioned around and within pcRNAs peaks distinctively at their TSSs (Fig. 3f). This is observed for pcRNAs with promoters located proximally and distally relative to the promoters of associated genes (Additional file 4: Figure S12b, c) and is consistent with the observed positioning of CTCF binding sites at the TSSs (Fig. 3a), indicating that the transcription of several pcRNAs starts at the base of topological loops in precise correspondence with CTCF anchors (Fig. 3a–c). Given this marked and precise association of pcRNA promoters with loop anchor points, we defined this group of 764 pcRNAs as ‘topological anchor point RNAs’ (tapRNAs), representing the subset of pcRNAs whose promoters overlap a loop anchor point (Fig. 3d).

Based on the chromatin state segmentation by HMM from ENCODE/Broad for nine cell lines [45], we found that the contacting loop anchor points are marked by multiple chromatin states and that a high proportion overlaps marks of active transcription and/or enhancers (Additional file 4: Figure S12d). tapRNAs are significantly more likely to be in contact with enhancer elements through such loops, compared to Gencode lncRNAs (*p* value = 2.85 × 10^−6^; Fig. 3g). This is not the case for the contact with promoter elements, transcribed regions or other HMM-defined genomic regions (Fig. 3g; Additional file 4: Figure S12e). These results indicate that tapRNAs are likely to be associated with enhancer sequences present on the other end of the loop. Moreover, inspection of ChIP-Seq data showed binding of several factors associated with looping and regulatory elements on both sides of the majority (> 90 %) of the tapRNA loops (Additional file 4: Figure S13a), including RNA polymerase II, p300, C/EBP, EZH2 and zinc finger proteins such as Znf143 (see e.g. [42, 46, 47]).

### Conserved domains and motifs in tapRNAs

**Fig. 4:**
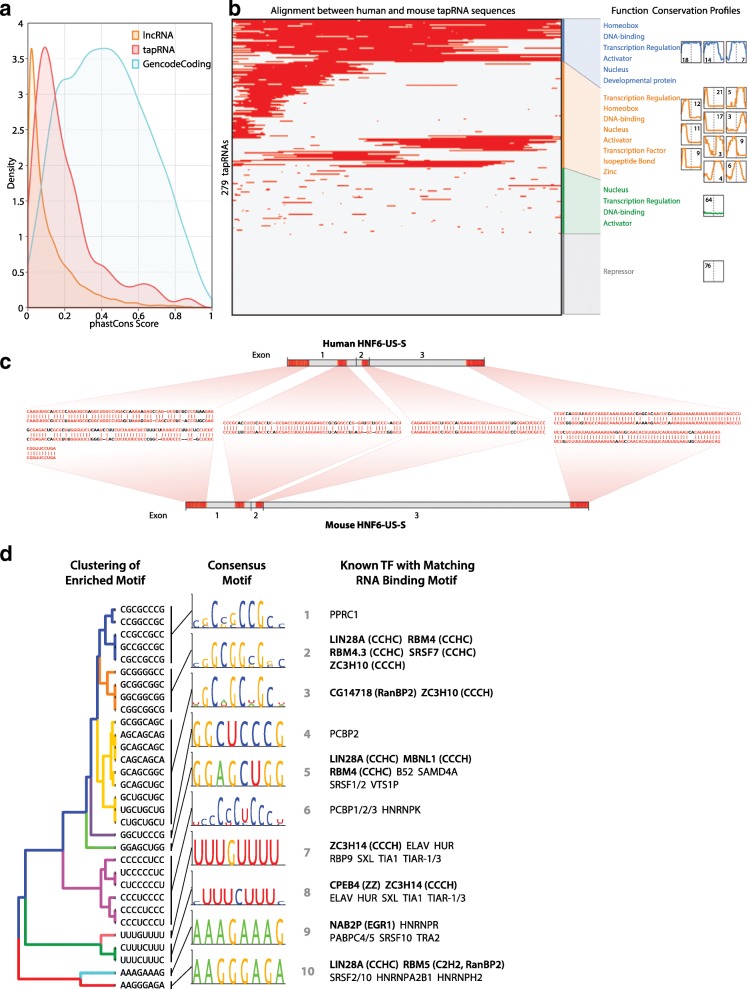
Conserved sequence motifs in tapRNAs. **a** Comparison of conservation between tapRNAs, lncRNAs and protein coding genes. The curves are kernel density estimation (KDE) of conservation scores calculated from the phastCons multiple alignments of 100 vertebrate species. **b** Clustered heatmap of conserved domains in transcribed tapRNAs. Aligned sequences (shown in *red*) in 279 non-redundant tapRNA isoforms are clustered (Euclidean distance). Sixteen minor clusters were identified and grouped into four major clusters. Each minor cluster’s centroids are shown with the number of tapRNAs belonging to each minor cluster. Thirty-nine tapRNAs (top group, *blue*) have a more than ~ 73 % conserved domain in their transcribed sequences. Functional category annotation search reveals that tapRNAs of the top group are highly related to developmental proteins or Homeobox proteins. In contrast, 76 tapRNAs of the bottom cluster (*grey*) do not have any sequence conservation and do not show significant common functionality. There are also some minor groups in which position-specific conservation is clearly present (e.g. 5′ end-specific or 3′ end-specific). **c** Example of conserved domains in a tapRNA. RNA sequence alignments of regions conserved between human and mouse *HNF6-US-S* tapRNA are represented in *red*. **d** Enriched RNA-binding motif in conserved domains of tapRNAs. Thirty-two significantly enriched 8-mer motifs (Additional file 2: Figure S13b; *p* value 1 × 10^−4^) in conserved domains in tapRNAs are identified and clustered into ten consensus motifs. *De novo* motif analysis discovers known RNA-binding proteins (RBPs) with matching binding consensus motifs. Seven out of ten consensus motifs are part of binding motifs of zinc finger proteins

We found that tapRNAs display higher sequence conservation across vertebrate genomes compared to generic Gencode lncRNAs, although this conservation is lower than that observed for protein-coding genes (Fig. 4a). This observation led us to further investigate whether there is any similarity of sequence that may provide clues to the function of tapRNAs. To this end, we applied a sliding-window direct RNA alignment approach, finding that 73 % of tapRNAs show high conservation in patches of sequence between human and mouse (Additional file 2: Supplementary methods), while the other 27 % lack these highly conserved patches (Fig. 4b). We divided the conserved 73 % into (a) high-, (b) medium-, and (c) low-conservation-region tapRNAs and carried out a GO enrichment analysis on their associated coding genes. In all cases, the predominant category of genes associated with conserved tapRNAs was linked to development. In contrast, the 27 % of tapRNAs with no highly conserved segments (d) were associated with genes showing no strong enrichment in functional categories. These data suggest that tapRNAs associated with developmental transcription factor genes have more significant sequence conservation than other tapRNAs.

The fact that we can detect discrete conserved sequence domains within the tapRNA group (Fig. 4b, c) prompted us to examine whether there are any sequence motifs in common between them. Motif enrichment analysis identified 32 8-mer motifs that were significantly more represented in the conserved domains of tapRNAs relative to non-conserved sequences (Fig. 4d; Additional file 4: Figure S13b). Closer inspection indicated that these 32 motifs were related and could be sub-categorised into ten consensus motifs. Analysis of the JASPAR database [48] of consensus sequences recognised by known RNA binding proteins (RBPs) found that each of the ten motifs has the potential to bind RBPs, including proteins involved in RNA metabolism and regulation (such as hnRNP A2/B1 and hnRNP K, HuR, ELAV and EGR1), with the predominant type (seven out of ten motifs) corresponding to binding motifs of RBPs containing different zinc finger (ZF) domains (Fig. 4d). This indicates that conserved sequences within tapRNAs may represent RNA binding motifs that regulate their processing and function. Furthermore, we found that the ten consensus motifs also match the binding motifs of known transcription factors, including a large number of ZF factors, such as CTCF, ZIC2 and ZNF263 (Additional file 4: Figure S13c). This association raises the possibility that some of the RNA aligned stretches between mouse and human tapRNAs might reflect an overlap to conserved transcription factor binding sites in the underlying DNA sequence, although the functional relevance remains to be determined (see “Discussion”).

### tapRNAs regulate neighbouring genes

**Fig. 5:**
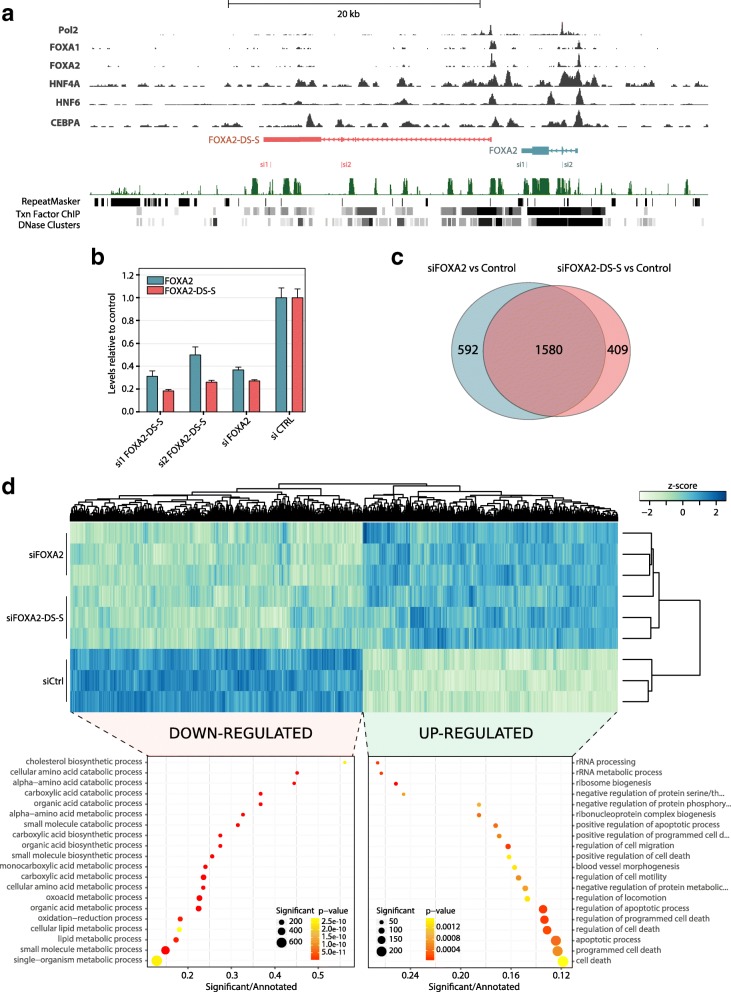
FOXA2-DS-S regulates FOXA2 expression. **a** Screenshot from the Dalliance genome browser [50] showing the FOXA2 locus with tracks displaying coverage data for ChIP-Seq experiments for Pol2, FOXA1, FOXA2, HNF4A, HNF6 and CEBPA. The ChIP-Seq tracks were produced by the ENCODE project on HepG2 cells. **b** Real time PCR data showing the expression of *FOXA2* and *FOXA2-DS-S* in Huh7 cells upon knock-down. Si1 and si2 FOXA2-DS-S indicate two different, non-overlapping siRNAs designed against *FOXA2-DS-S*. The data are expressed relative to the expression of the control transfected with scrambled siRNAs; the *error bars* indicate the standard error of the mean across three replicate experiments. **c** Venn diagram showing the number of significantly differentially expressed genes (adjusted *p* value < 0.05 and log2 fold change > or < 1.25) in the microarray experiment on Huh7 knock-down of *FOXA2* or *FOXA-DS-S*. **d** Heatmap showing microarray data upon knock-down of *FOXA2* or *FOXA-DS-S* in Huh7 cells. The colour scale indicates normalised intensities (z-score). The heatmap contains all genes that were significantly altered (adjusted *p* < 0.05) upon knoc- down of either *FOXA2* or *FOXA-DS-S*. The scatter plots in the lower part of the panel show GO enrichment data for genes that were significantly down-regulated (*left*) or up-regulated (*right*) in either siFOXA2 or siFOXA-DS-S

Given that tapRNAs and their neighbouring genes are co-expressed in a tissue-specific manner, we investigated their ability to regulate each other’s expression. First, we tested this hypothesis on a key liver master regulator, *FOXA2*, and its associated tapRNA. ChIP-Seq data [49, 50] show that the promoters of the *FOXA2* gene and *FOXA2-DS-S* are occupied by a similar set of transcription factors, and importantly, the same key regulators of liver differentiation (*FOXA1*, *FOXA2*, *HNF4A* and *HNF6*; Fig. 5a; Additional file 4: Figure S14). This again suggests that the mechanism of co-expression and co-induction is due, at least in part, to specific factors concomitantly regulating expression of both the coding and noncoding transcripts.

We next investigated whether *FOXA2-DS-S* can affect the expression of the associated coding gene, finding that *FOXA2-DS-S* is necessary for full expression of the *FOXA2* gene (Fig. 5b; Additional file 4: Figure S15a), since its down-regulation by RNA interference results in reduction of *FOXA2* expression in Huh7 liver cancer cells (Fig. 5b) and A549 lung cancer cells (Additional file 4: Figure S15a). Interestingly, knock-down of *FOXA2* also leads to down-regulation of *FOXA2-DS-S* (Fig. 5b; Additional file 4: Figure S15a). These results indicate that *FOXA2* not only auto-regulates by binding its own promoter (Additional file 4: Figure S14), but also affects the expression of the associated tapRNA, providing a positive feedback loop and suggesting interdependence.

Microarray analysis of the global transcriptional effects of *FOXA2-DS-S* or *FOXA2* knock-down showed a large overlap in the repertoire of affected genes (Fig. 5c, d). This suggests that the major target for *FOXA2-DS-S* is the *FOXA2* gene, although additional direct targets may yet be identified. These findings were recently independently supported by the *cis*-regulation of *FOXA2* in differentiating definitive endoderm cells by *FOXA2-DS-S* (also known as *DEANR1*, or ‘*definitive endoderm-associated lncRNA1*’), indicating that this lncRNA regulates *FOXA2* in different endoderm-derived tissues [51].

We obtained similar results for a tapRNA associated with a second liver factor, *HNF1A*, and five other tested tapRNAs in different cell lines (*FOXD2-AS*, *SETD1B-BT*, *POU3F3-BT* and *NR2F1-BT*; Additional file 4: Figure S8 and S15a–d). These were chosen among tapRNAs specifically co-expressed with the associated genes in different tissues and cell types, and in each of these cases, knock-down of the tapRNA reduces expression of the associated coding gene. Interestingly, knock-down of *HNF1A-BT1* reduces expression of *HNF1A* (Additional file 4: Figure S8a) but ectopic over-expression of full-length *HNF1A-BT* in human liver cells had no effect on the associated gene, even though very high levels of over-expression were achieved (data not shown), again suggesting a *cis*-based context-dependent mode of regulation. Although the functional association with chromatin looping needs to be systematically tested (see “Discussion”), we posit that novel tapRNAs co-expressed with their associated genes in mouse and humans are strong candidates for *cis*-regulators with important cellular roles. For instance, a recent large-scale phenotypic screen by CRISPR-interference has identified numerous lncRNAs essential for cellular growth [3]. This set was enriched for pcRNAs and tapRNAs (highlighted in Additional file 3: Table S2; *p* values = 1.14 × 10^−26^ and 6.51 × 10^−13^, respectively) and included numerous antisense and bidirectional tapRNAs (respectively, 28 and 40 tapRNAs), such as *FOXD3-BT* and *NKX1–2-AS*, as well as more distal ones (25 tapRNAs), such as *SOX4-DS-S* and *MTX2-DS-AS* (*TOG*) (Additional file 3: Table S2).

### tapRNAs are implicated in cancer

The Nanostring analysis of the cancer cell panel also demonstrated specific expression of pcRNAs, including many of the tapRNAs and associated genes, in different cancer lines (Additional file 4: Figure S16a–e). lncRNAs are already considered important players in disease [1, 52, 53] and many genes with roles in development have been previously linked to cancer and other disorders [54]. We therefore investigated the possible involvement of different pcRNAs in cancer cells. To explore this association, we performed a meta-analysis of the expression of pcRNAs in normal versus tumour samples in 63 microarray studies. After re-annotating the microarrays to identify probes targeting pcRNAs, we identified 203 pcRNAs significantly differentially expressed in tumours compared to normal tissues (Additional file 4: Figure S17a and Additional file 8: Table S6). These included known cases of lncRNAs involved in different cancers, such as *GAS5*, *DLEU2*, *PART1* and *MEG3*. We expanded this analysis to The Cancer Genome Atlas (TCGA) RNA-Seq data available for 24 different cancer types and over nine thousand samples [55] and verified that 443 pcRNAs were annotated and differentially expressed in this dataset (Additional file 4: Figure S17b). Among these, we catalogued the 203 tapRNAs that are differentially expressed in tumours compared to normal tissues, forming clusters of expression that clearly distinguish cancer types (Additional file 5: Figure S18a and Additional file 3: Table S2).

**Fig. 6:**
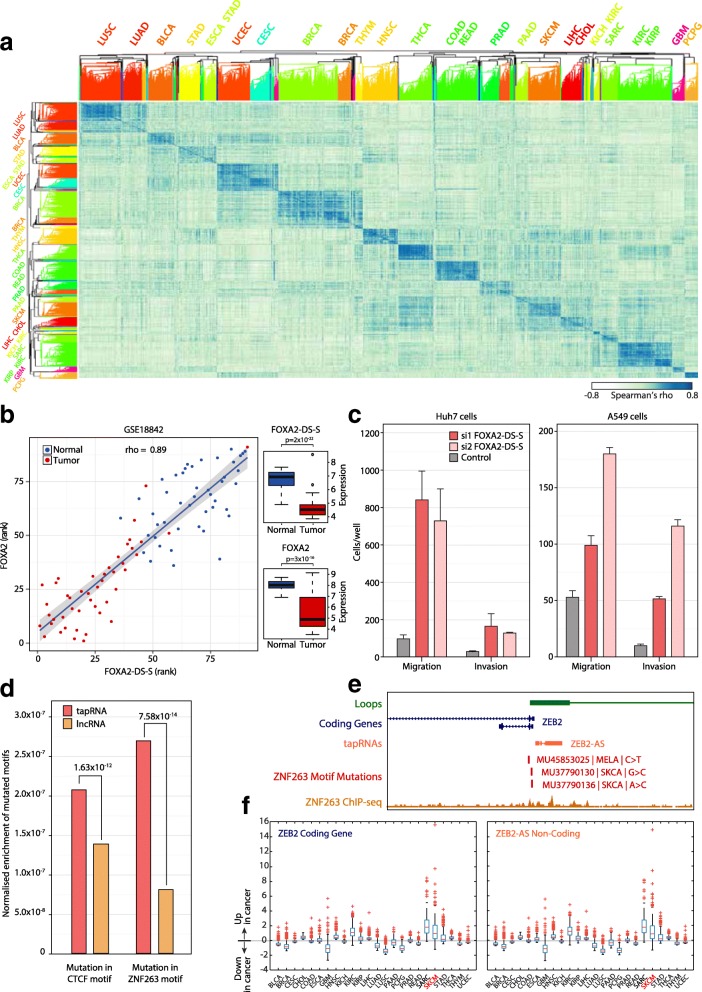
pcRNAs are differentially expressed in cancer. **a** Spearman’s rank-order correlation heatmap between tapRNAs and their associated coding genes in TCGA RNA-Seq V2 level 3 data. The correlation was calculated between the two matrices of TCGA RNA-Seq fold changes (Additional file 2: Figure S18a, b) and shows that the expression of pcRNAs and corresponding coding genes is correlated within specific cancers. **b** Spearman correlation between the expression of *FOXA2* and *FOXA2-DS-S* in lung cancers (GSE18842 dataset). Tumour and normal individual samples are represented as *blue* and *red dots*, respectively. Boxplots on the right show that both transcripts are down-regulated in tumour compared to normal samples (Student’s *t*-test *p* values are indicated). **c** Invasion and migration assay analysis of Huh7 (*left*) and A549 (*right*) cells upon knock-down of *FOXA2-DS-S* using two different siRNAs (si1 and si2) compared to negative control siRNA. The bars show the mean of three biological replicate experiments. The error bars indicate the standard error of the mean. **d** Mutational analysis of CTCF and ZNF263 motifs associated with tapRNA loci. CTCF and ZNF263 motifs inside of tapRNA loci have significantly higher chances to be mutated in cancer. In total, we catalogued 241 CTCF motif mutations in 171 motif sites (37 cancer types) and 196 ZNF263 motif mutations in 135 motif sites (27 cancer types). **e** Example of a mutational analysis of CTCF and ZNF263 motifs associated within the ZEB2/ZEB2-AS/BT tapRNA locus, depicting the mutations found in melanomas. **f** Expression profile of ZEB2 and ZEB2-AS/BT in different cancers, showing concordant increased expression in malignancies, including skin cutaneous melanoma (*SKCM*)

tapRNA expression in different cancers is largely mirrored by the expression of the associated coding genes (Additional file 4: Figure S18a, b), consistent with the positive correlation observed in normal tissues. Representation of the correlation of expression between tapRNAs and associated genes (*p* value = 1.13 × 10^−11^) showed marked positive correlation signatures, which contain several tapRNA–gene pairs with altered expression in different cancer types (Fig. 6a; Additional file 3: Table S2). Close inspection of individual tapRNA–gene pairs with altered expression in primary tumours showed that a number of them are consistently down- or up-regulated in cancer compared to normal tissues (Additional file 8: Table S6). For example, *FOXA2* and its tapRNA *FOXA2-DS-S* were found significantly down-regulated in lung tumour compared to normal samples (*p* values = 3 × 10^−16^ and 2 × 10^−22^, respectively), effectively separating tumour samples from controls (Fig. 6b).

To investigate whether *FOXA2-DS-S* tapRNA has a functional effect in cancer, we knocked it down in Huh7 and A549 cells (Fig. 5b; Additional file 4: Figure S15a). A dramatic increase in both cell invasion and migration capacity was observed, supporting a tumour suppressor function for *FOXA2-DS-S* transcripts similar to the reported role of *FOXA2* in cancer cells [56, 57] (Fig. 6c; Additional file 4: Figure S15e). These data further support the close functional link between the tapRNA and its associated gene. The fact that *FOXA2-DS-S* has the same effect on the phenotypic characteristics of cancer cells as its associated gene is consistent with its positive effect on the expression of *FOXA2* and the observation that it regulates a similar cohort of genes (Fig. 5c, d). We also analyzed the role of two other tapRNA–gene pairs in invasion and migration characteristics of the cancer cells in which they are expressed (Additional file 4: Figures S16 and S17a). We found that knock-down of *NR2F1-BT* and *NR2F1* or *POU3F3-BT* and *POU3F3* similarly reduced the invasion/migration potential of U251MG glioblastoma and U2OS osteosarcoma cells (Additional file 4: Figure S15f–h). Thus, in the case of *NR2F1-BT* and *POU3F3-BT*, their effect on cell invasion/migration suggests that tapRNAs may also have oncogenic roles in these cells, likely involving the regulation of the associated genes.

Finally, considering the recent involvement of CTCF and architectural mutations in activation of oncogenes in specific tumours [58, 59], which represent hitherto overlooked potential causative mutations, we performed a mutational analysis of CTCF motifs associated with tapRNA loci. We observed a significantly more frequent mutation of CTCF sites in tapRNA promoters and transcribed regions compared to Gencode lncRNAs (*p* value = 1.63 × 10^−12^; Fig. 6d). Given our finding that binding sites for additional zinc finger motif transcription factors are enriched in tapRNA conserved sequences (Additional file 4: Figure S13c), we asked if mutations in cancer are also present in ZNF263 sites, which was among the highly enriched motifs associated with tapRNAs and for which there is available ChIP-Seq data (Fig. 3e; Additional file 4: Figure S13c). Indeed, we observed an even more marked enrichment for mutations in ZNF263 sites in cancers than CTCF (*p* value = 7.58 × 10^−14^; Fig. 6d).

We investigated whether there was a connection between these mutations and the expression of tapRNAs, using matched tissues for TCGA expression data and cancer mutation. We found evidence that for 25 tapRNAs the mutation of CTCF (19 tapRNAs) or ZNF263 (7 tapRNAs) coincides with a significant change in tapRNA expression in the equivalent cancer (Additional file 9: Table S7). These included 16 mutated sites with ChIP-sequencing evidence for CTCF/ZNF263 binding, indicating that the mutation may interfere with TF binding and regulatory activity, and encompassed *cis*-acting lncRNAs previously implicated in cancer, such as *HOTAIRM1* (*HOXA1-AS*) [60], *HOTTIP* (*HOXA13-BT*) [61], *ZEB1-AS* [62] and *ZEB2-AS* [63, 64]. For example, mutations in ZNF263 binding sites are found in the promoter of *ZEB2-AS*/*ZEB2*, associated with altered expression of both the tapRNA and *ZEB2*, which is a master-regulator of epithelial-to-mesenchymal transition (EMT) and metastasis [65, 66] (Fig. 6e, f). This raises the possibility that, in addition to CTCF binding sites, mutations in ZNF263 and other motifs associated with tapRNAs may be involved in cancer. Taken together these data highlight the fact that tapRNAs can be considered as potential targets for cancer and that their expression pattern may act as a marker for the disease.

## Discussion

### Syntenic lncRNAs with promoter conservation linked to developmental genes

In this work, we systematically identify, on a genome-wide scale, lncRNAs with conserved promoters in mouse and human, cataloguing 665 lncRNA promoters that are genomically linked to 626 coding genes. This analysis has identified a subset of positionally conserved lncRNAs with a close relationship to a very specific cohort of neighbouring protein-coding genes, predominantly comprised of transcription factors. Our analysis indicates that positionally conserved lncRNAs and their associated developmental transcription factors have some common characteristics: they are (a) expressed in the same restricted tissues in mouse and human and (b) co-induced when cells are stimulated with a differentiation signal, (c) have promoters bound by similar transcription factors and (d) can affect each other’s expression.

Consistent with our findings, many lncRNAs defined here as tapRNAs have been shown to regulate developmental genes in a manner implying local regulation. These include *HOTTIP* (*HOXA13-AS*), *HOTAIRM1* (*HOXA1-AS*), *HAGLR* (*HOXD1-AS*), *NKX2–1-AS and DEANR1* (*FOXA2-DS-S*) [12, 22, 53, 67]. TapRNAs may add to a growing list of lncRNAs that regulate neighbouring genes *in cis*, via the RNAs themselves and/or their act of transcription [4, 13, 14].

We have annotated pcRNAs using a systematic nomenclature that reflects the conserved orientation of the pcRNA relative to the neighbouring coding gene. We believe that this catalogue will be a valuable resource in the characterization of these important coding genes and their associated noncoding RNAs.

### tapRNAs

The most striking feature of pcRNAs is their enrichment in tapRNAs, defined as RNAs found at chromatin loop anchor points and at boundaries of topological domains. These architectural landmarks are commonly occupied by the CTCF factor and indeed we find an enrichment of CTCF binding in the promoters of tapRNAs and within their conserved domains. The precise number of tapRNAs within the cohort of pcRNAs depends on certain definition criteria. We have taken a conservative approach and defined tapRNAs as those that have their promoters directly overlapping loop anchor points. If we consider those overlapping either an anchor point or a TAD boundary we instead identify 54 % (912) of pcRNA transcripts as tapRNAs, but more relaxed definitions of pcRNAs (for example including non-spliced transcripts or pcRNAs that are more clade-restricted and have lower promoter conservation) as well as high-resolution HiC data from a broader set of tissues and developmental stages will likely increase this figure.

tapRNAs have specific features that allow them to be defined as a distinct group of lncRNA: they (a) overlap topological anchor points, (b) have the architectural zinc finger protein CTCF bound within their promoter at much higher levels than other lncRNAs, and (c) have conserved domains within the transcripts that are enriched in consensus binding sites for zinc finger RBPs. Below we discuss these features associated with tapRNAs and how they may relate to the regulation of the associated developmental genes.

### tapRNAs and chromatin looping

The genome is highly structured and gene expression influences and is influenced by the topological organization of the chromatin [42, 43, 68–71] (see [42] for review). The causality of the association of lncRNA and transcription with chromatin topology is a matter of current investigation [72, 73]. Likewise, the extent to which the common properties of tapRNAs reflect the overlap with genomic features associated with conserved developmental loci is unknown. Nevertheless, lncRNAs are emerging as important actors in the regulation of nuclear architecture [7, 43, 68, 69] and several characterised lncRNAs, defined here as tapRNAs, have been implicated in chromatin looping and, in some of these cases, the regulation of neighbouring genes. These include several *HOX* loci-associated RNAs such as *HoxBlinc, HOTTIP*, *EVX1AS* and *HOTAIRM1* [12, 20, 74–77]. These reports provide experimental evidence that tapRNAs may be commonly implicated in regulating genes by affecting topological conformations. These may involve different roles for spliced or non-spliced isoforms of tapRNAs, as recently demonstrated for *HOTAIRM1* in the regulation of the immediate neighbouring genes and other *HOXA* genes within the looping region in differentiating NT2 cells [76]. Moreover, transcription of lncRNA loci associated with developmental genes can dictate nuclear compartmentalization and direct looping between associated enhancers and gene promoters. This has been recently demonstrated for the *ThymoD* (*thymocyte differentiation factor*) lncRNA, which regulates the neighboring *Bcl11b* gene by modulation of CTCF binding and cohesin-dependent looping in T-cell fate specification [78].

Further indications of such topological connections come from many other uncharacterised tapRNAs. These also include tapRNAs within the *HOXD* cluster, whose TAD boundaries and chromatin loops are specifically regulated during embryonic development, and are associated with differential *HOX* gene expression [44, 71, 79]. These boundaries are marked by syntenic lncRNAs such as *HOG* and *TOG* [44], but also additional non-annotated tapRNAs (Fig. 3e). Other important genes involved in cell-fate determination, such as *FOXA2* and *PDX1*, also have positionally conserved lncRNAs associated with their TAD boundaries [80, 81].

These data indicate that regulation of chromatin organization and of expression of developmental genes may be a common property of tapRNAs in mammals.

### Conserved domains and motifs within tapRNAs

Analysis of sequence conservation among tapRNAs has shown that this group of RNAs is more conserved than the bulk of lncRNAs. By direct alignment of human and mouse tapRNAs, we observed stretches of high sequence identity, which may reflect overlap of DNA regulatory regions within tapRNA loci, but which is also consistent with regions of ‘microhomology’ and of possible functional conservation in lncRNAs [5, 17, 29, 82, 83]. Within the conserved sequences of tapRNAs, ten degenerate motifs were found enriched. These motifs are similar to the consensus binding sites of known RBPs, predominantly of the CCCH zinc finger family. Although many of these are likely involved in RNA processing, the enrichment in conserved stretches of tapRNAs may also underscore a role in tapRNA regulation and function. Interestingly, an analysis of 58 diverse RBPs in human cells indicated that the majority of those RBPs also interact with chromatin in a genome-wide fashion, including a large fraction of gene promoters and regulatory sequences [84], suggesting the occurrence of co-transcriptional deposition of RBPs on target RNA substrates and a possible impact on genome regulatory processes [4, 85, 86].

We noticed that the ten conserved motifs in tapRNAs also correspond to consensus binding sites of transcription factors. Three of them are also enriched within enhancers on the other end of the loop anchor point (Additional file 4: Figure S13c). These three motifs all have the potential to bind ZF proteins, including Zic2, which has been associated with chromatin looping and enhancer function [87]. This raises the possibility that the mechanism of action of ZF motifs within tapRNAs is related to the presence of similar ZF motifs and DNA-bound proteins at enhancers. One mode of action could be that tapRNA ZF motifs could induce the sequestration or delivery of ZF factors to enhancers, for example involving the formation of RNA–DNA hybrids or triplex structures [88, 89]. In all these scenarios, the tapRNAs could have an effect on the transcription of the associated developmental coding gene by modulating the presence of ZF transcription factors on enhancers. Binding of the YY1 transcription factor to RNA has recently been demonstrated to play a role at enhancers [90], supporting the argument that RNA–transcription factor interactions may influence enhancer activity. Finally, we also found evidence that mouse tapRNAs are enriched in CLIP-Seq peaks for CTCF [91] (Additional file 4: Figure S13d, e), suggesting that regulation of CTCF binding may also be possible, as indicated by previous work that identified lncRNA binding of CTCF regulating chromatin looping and expression of neighbouring genes [78, 92].

### tapRNAs and cancer

Several lines of evidence presented here are consistent with the role for tapRNAs in cancer development. Firstly, a cancer microarray meta-analysis and the TGCA data show that tapRNAs are misregulated in selected tumour types. Second, we obtained a direct indication of their involvement in cancer from the fact that manipulation of their expression levels affects the phenotypic characteristics of cancer cells *in vitro*, such as invasion and migration. Once again, the associated coding genes, which can often be oncogenes or tumour suppressors, show a similar effect on invasion and migration. For example, *FOXA2* is a tumour suppressor and inhibitor of EMT in human lung cancers [57, 93] and reduced *FOXA2* expression in hepatocellular carcinoma (HCC) is associated with worse clinical outcome. Here, our data also suggest the implication of the tapRNA *FOXA2-DS-S* in this process, as well as other tapRNAs affecting metastatic characteristics of different cancer cells (Additional file 4: Figure S15).

Another indication of the involvement of tapRNAs in cancer comes from the finding that their promoters and/or gene bodies are enriched in mutations linked to cancer. Mutations in the binding site for the zinc finger protein CTCF have already been described [58, 59], and we find that tapRNAs overlap CTCF sites that are found mutated in cancer. In addition, we identify a second zinc finger protein (ZNF263) enriched in the conserved sequences of tapRNAs, which is also mutated in cancer cells. Our analysis highlights the possibility that ZNF263 and potentially other zinc finger factors may play a role in genomic organization, regulating genes that have critical roles in cancer.

## Conclusions

In this study we have defined tapRNAs as a new subset of lncRNAs with common structural and functional features. Positional conservation was used as the original criterion for this grouping, and a set of developmental genes was identified as co-regulated with these RNAs. This suggests the existence of an ‘extended gene structure’ where a small proportion of coding genes (3 %) are connected genomically with long noncoding RNAs in a functional unit. In addition, we found that in different cases the protein-coding gene may regulate itself and also the associated lncRNA, indicating the existence of an intimate connection between these pairs and of positive feedback loops that may confer robust tissue-specific expression. We expect this number of tapRNA–gene pairs to be an underestimate given the stringent criteria set for the current analysis. However the most important and unexpected common feature of tapRNAs is their genomic location at topological anchor points. This genomic positioning and the enrichment of CTCF motifs in the promoter and body of tapRNAs strongly suggest a role for these RNAs in genomic organization. The fact that tapRNAs overlap CTCF and ZNF263 motifs that are mutated in cancer also points to genomic organization as an important node in homeostasis and disease. The involvement of tapRNAs in higher order architectural organization may be particularly important for the expression of developmental genes and for their misregulation in cancer.

## Methods

### pcRNA cloning

Cloning of human full-length HNF1A-BT1 transcript was performed using Gateway Technology (Thermo Fisher Scientific, catalogue number 12535–019) according to the manufacturer’s instructions. Briefly, 2 μg total RNA from HepG2 cells were reverse transcribed in 20 μl reaction using Superscript III Reverse Transcriptase (Invitrogen, catalogue number 18080044). Touchdown-PCR was performed using 2 μl of the cDNA, mixed with 38.75 μl water, 1 μl of each primer (10 μM) (Additional file 10: Table S8), 1.25 μl dNTP (10 mM), 5 μl 10× Pfu Ultra reaction buffer and 1 μl Pfu Polymerase (Stratagene, catalogue number 600380). PCR was performed in the following conditions: (i) 98 °C for 30 s, (ii) 98 °C for 10 s, (iii) 70–50 °C for 30 s, (iv) 72 °C for 3 min (with 20 cycles repeating steps ii–iv, thereby decreasing the temperature of step iii 1 °C per cycle); followed by (v) 98 °C for 10 s, (vi) 50 °C for 30 s, (vii) 72 °C for 2 min, (viii) 72 °C for 5 min, with 15 cycles repeating steps v–vii. Nested PCR was performed with PCR products after gel extraction and 1:100 dilution. The cycling conditions were: (i) 98 °C for 30 s, (ii) 98 °C for 10 s, (iii) 59 °C for 30 s, (iv) 72 °C for 2 min, (v) 72 °C for 5 min, with 30 cycles repeating steps ii–iv. Gel purified PCR products were quantified and transferred into the pDONR221 entry vector (Life Technologies, catalogue number 12536–017). Transformations were performed according to the Gateway clonase protocol using *Escherichia coli* DH5α and the plasmids used in an LR reaction to generate the expression vectors using the LINC-EXPRESS plasmid (modified pLENTI6.3/TO/V5-DEST, kindly provided by John Rinn [94]).

### Cloning of shRNAs

Short hairpin (sh)RNA design was performed using the Broad Institute RNAi Consortium software (http://www.broadinstitute.org/rnai/public/seq/search), which was used to select three or four different candidate shRNAs per pcRNA. We tested their knockdown efficiencies in transient transfections and the effective shRNAs as well as negative control were used in subsequent experiments (Additional file 10: Table S8). Cloning of the annealed shRNA oligos into pLKO vectors for shRNA constructs was performed as described in the TRC Laboratory Protocol (version 2/12/2013). Briefly, HPLC-purified oligomers were annealed using final concentrations of 3 μM each in 1× NEB buffer 2 and used in ligations into pLKO vectors digested with AgeI and EcoRI (pLKO.1 puro (addgene #8453) [95] and Tet-pLKO-puro (addgene #21915) [96]. Ligation products were used for transformations of *E. coli* DH5α and plasmids were purified and sequenced.

### Cell culture

Cell lines (Additional file 10: Table S8) were acquired from ATCC and cultured at 37 °C and 5 % CO_2_ in the recommended media unless specified. Huh-7 and HepG2 cells were grown in growth medium (Dulbecco’s modified Eagle’s medium (DMEM), 10 % fetal bovine serum (FBS), 2 mM L-glutamine, 50 μg/ml penicillin and 50 μg/ml streptomycin) at 37 °C and 5 % CO_2_. Human erythroleukemia (K562) cells were cultured in RPMI 1640 media supplemented with 10 % FBS and 2 mM glutamine, and breast adenocarcinoma (MCF7), lung adenocarcinoma (A549), osteosarcoma (U2OS) and glioblastoma (U251MG) cells were cultured in DMEM media supplemented with 10 % FBS. Mouse embryonic stem cells (E14) were cultured in 1 % gelatin-coated dishes in DMEM media with 2 mM glutamine, 1 mM sodium pyruvate, 1× non-essential amino acids, 10 % FBS, 0.1 mM 2-mercaptoethanol, and supplemented with 1000 U/mL LIF (ESGRO). Human teratocarcinoma NT2/D1 cells were cultured in DMEM supplemented with 10 % FBS. For induction of differentiation, NT2 cells and mouse ES cells were cultured in media without LIF and treated with 10 μM all-*trans* retinoic acid (Sigma R 2625) and harvested at different time points.

### Knock-down and over-expression

shRNA and siRNA oligonucleotides were ordered from Thermo Fisher Scientific or as Qiagen FlexiTube siRNAs (Additional file 10: Table S8). Transfections for siRNA-mediated knock-down experiments were performed according to the Lipofectamine RNAiMAX (Thermo Fisher Scientific, catalogue number 13778150) procedure. Briefly, the day before transfection 1 × 10^5^ cells were seeded in 2.5 ml DMEM/10 % FBS in six-well plates. For each well, 50 nM siRNA duplexes were diluted in 250 μl Opti-MEM. Lipofectamine RNAiMAX (5 μl) was added to 245 μl Opti-MEM and combined with the siRNA mix. After incubation for 10–20 min at room temperature (RT) the mix was added dropwise to the cells. Cells were incubated for 48 h until harvest. For over-expression, the day before transfection 1 × 10^5^ cells per well were plated in six-well plates in 2 ml growth medium without antibiotics. Transfections were performed following the Fugene6 Transfection Reagent Protocol (Promega, catalogue number E2691). Briefly, 185 μl of medium was premixed with 5 μl transfection reagent per well in a six-well plate. After 5-min incubation at RT, 10 μl plasmid DNA (1 μg) were added to the mix and incubated for 30 min at RT. Subsequently the transfection reagent–DNA mixture was added dropwise to each well. The transfected cells were incubated for 48 h before processing.

### RNA isolation and RT-PCR expression analysis

RNA from cell cultures was purified using Qiazol (Invitrogen) and RNeasy Mini Kit (Qiagen) or Direct-zol RNA MiniPrep kit (Zymo Research, catalogue number R2072) and treated with DNase I (Invitrogen), according to the manufacturers’ instructions. Purified tissue RNA was purchased from Ambion (FirstChoice Human Total RNA Survey Panel, catalogue number AM6000) and Clontech (Mouse Total RNA Master Panel, catalogue number 636644) (Additional file 10: Table S8). RNA quality was assessed using the 2100 Bioanalyzer (Agilent Technologies) prior to further use. cDNA preparation and quantitative real-time PCR (qPCR) analysis were performed as previously described [20]. Each experiment was performed in at least two biological replicates. Primers were designed spanning splice sites in most cases and for normalization of transcript expression levels, *B2M*, *ALAS1* or *GAPDH* primers were used (Additional file 10: Table S8).

### Invasion-migration assays

We plated 2.5 × 10^4^ cells in serum-free media in an insert plate upper chamber with either non- or Matrigel-coated membranes (24-well insert; pore size, 8 μm; BD Biosciences, catalogue number 354578 and 354,480) for trans-well migration and invasion assay, respectively. The bottom chamber contained DMEM with 10 % FBS. After 24 h, the bottom of the chamber insert was fixed and stained with crystal violet and cells on the stained membrane were counted under a microscope. Each membrane was divided into four quadrants, and an average from all four quadrants was calculated. Each assay was performed in biological triplicates.

## Additional files

Additional file 1: **Table S1.** List of RNA-Seq and ChIP-Seq datasets used in the study. (XLSX 304 kb)

Additional file 2: Supplementary information. (PDF 324 kb)

Additional file 3: **Table S2.** Annotation of pcRNAs. (XLSX 494 kb)

Additional file 4: Supplementary figures. (PDF 14168 kb)

Additional file 5: **Table S3.** GO enrichment of pcRNA-associated protein coding genes. (XLSX 65 kb)

Additional file 6: **Table S4.** GO enrichment protein coding genes associated with pcRNAs in each possible orientation. (XLSX 54 kb)

Additional file 7: **Table S5.** Nanostring data. (XLSX 232 kb)

Additional file 8: **Table S6.** Metanalysis of pcRNA expression across different cancer studies. (XLSX 190 kb)

Additional file 9: **Table S7.** List of tapRNAs with mutated CTCF and/or ZNF263 sites. (XLSX 115 kb)

Additional file 10: **Table S8.** Oligonucleotides, clones and cell lines used in this study. (XLSX 11 kb)
