# Supplementary material for: Genomic positional conservation identifies topological anchor point RNAs linked to developmental loci
Source: Genome Biol. 2018 Mar 15;19:32. doi: 10.1186/s13059-018-1405-5 (PMC5853149; doi:10.1186/s13059-018-1405-5)
Supplement: Supplementary file 2 — Supplementary information. (PDF 324 kb) [file 13059_2018_1405_MOESM2_ESM.pdf]

Supplementary Information

Supplementary Information 1

Supplementary Figure Legends 3

Supplementary Table Legends 9

Supplementary Methods 10

Human and Mouse reference genomes 10
Human and Mouse reference transcriptomes 10
Genbank all RNAs 10
RNA-Sequencing data analysis 10
Mapping 10
Assembly 10
Abundance estimation and expression normalisation 11
Identification of pcRNAs 12
Human Data preparation 12
Mouse Data preparation 12
Identification of conserved promoters 13
Non-coding to coding positional annotation 13
Human-mouse positional comparison 14
Annotation of pcRNA genomic characteristics 14
Characterisation of pcRNA features and expression analysis 15
pcRNA expression heatmaps 15
pcRNA expression distance heatmaps 15
GO enrichment of pcRNA-associated coding genes 15
Correlation of expression between pcRNAs and coding genes and between human and mouse pcRNAs 15
Tissue specificity score and GO enrichment by tissue 16
Human-mouse conservation analysis 16
CAGE analysis 16
Splice sites conservation profiles 16
Subcellular localisation analysis 16
Identification of pcRNAs targeted by CRISPR-interference (CRISPRi) 17
Identification of Pfam domains in pcRNAs 17
Nanostring analysis 18
FOXA2-DS-S knock-down microarray analysis 19
pcRNA histone modification profiles 20
Analysis of H3K27me3 in ESCs 21
ENCODE TF ChIP-seq data analysis 22
Known TF-binding motif data analysis 23
Identification of CTCF binding sites in pcRNA promoters 24
Identification of HiC loops that overlap pcRNAs 25
TAD/Loop Boundary Enrichment Analysis 26
PhastCons Conservation Analysis 27
Conserved domain search 28
Motif search in conserved domains 29
Consensus motifs and De novo motif discovery 30
Enriched motif search in enhancer region of the other end of loop anchor points 31
CTCF CLIP-seq data analysis 32

Microarray meta-analysis 33
The Cancer Genome Atlas (TCGA) RNA-seq meta-analysis 34
International Cancer Genome Consortium (ICGC) pan-cancer somatic mutation analysis... 35

Supplementary References 36

Supplementary Figure Legends

Supplementary Figure S1: Characterisation of pcRNAs A: Bar chart showing the number of pcRNAs in each orientation. **B**: Density distribution of the distance between pcRNAs and respective coding genes, color-coded by positional orientation. The left plot shows pcRNA in antisense orientations, while the right plot shows pcRNAs in sense orientations. **C:** Bar chart showing exon-number distribution for each pcRNA. **D**: Boxplot showing the distribution of the distances between the TSS of pcRNAs and the TSS of their corresponding coding gene.

Supplementary Figure S2: Conservation of pcRNAs
A: Plot showing the cumulative distribution of the distance of the closest FANTOM5 CAGE tag from the TSS of each pcRNA. The y-axis reports the fraction of pcRNAs that have at least one CAGE tag at the distance reported on the x-axis (or less) from their TSS (upstream or downstream). **B:** Boxplot showing the fraction of sequence identity between human and mouse pcRNAs and human and mouse pcRNA-associated protein coding genes. Sequence identity was calculated with the Needleman-Wunsch algorithm [1]. **C**: Plot showing the average phastCons conservation score across 100 vertebrate genomes (see Supplementary Methods) for the first, last and internal exons of pcRNAs. The shaded area around the line reports the standard error of the mean. **D:** Left**:** Distribution of the inverse logit of the Relative Concentration Index (RCI) between nucleus and cytoplasm for protein coding genes, Gencode lincRNAs, Gencode ncRNAs and pcRNAs. A value of 0 indicates exclusively nuclear localisation whereas 1 indicates exclusively cytoplasmic localisation. Right: distribution of the inverse logit of the Relative Concentration Index for pcRNAs and Gencode lincRNAs subdivided into 3 groups of matched expression in whole cells. Data from the lncATLAS database [2]. **E:** GO enrichment analysis of pcRNA-associated protein coding genes using a background gene set with matched intergenic distance (i.e. the sum of the distances of the closest upstream and downstream genes, see Supplementary Methods). The plot on the left shows the distribution of intergenic distance for pcRNA-associated coding genes and for the background set used in the GO enrichment analysis.

Supplementary Figure S3: Expression analysis of pcRNAs A,B: Heatmap showing the expression profiles of human (A) and mouse (B) pcRNAs across tissues and cell lines. The horizontal sidebar reports the tissue specificity score of pcRNAs, ranging from 0.27 (white) to 1 (red). **C:** Density distribution of the Spearman's correlation coefficients between human and mouse pcRNA pairs based on the RNA-Seq expression data. Mean Spearman's rho between human and mouse 0.26, permutation test p-value $<10^{-6}$. The dotted line shows the background distribution of all pairwise Spearman's correlations between human and mouse pcRNAs. **D**: Density distribution of the Spearman's correlation coefficients between human and mouse pcRNA pairs as in (C) but based on Nanostring data. Mean Spearman's rho 0.33, permutation test p-value $<10^{-6}$. **E**: Density distribution of the Spearman's correlation coefficients between human pcRNAs and corresponding coding genes

based on Nanostring data. Mean Spearman's rho 0.40, permutation test p-values $<10^{-6}$. The dotted line shows the background distribution of all pairwise Spearman's correlations between pcRNAs and pcRNA-associated coding genes. **F:** Plot showing the Spearman correlation coefficient between the expression of pcRNAs and their corresponding coding genes as a function of their distance (TSS to TSS), indicating independence of TSS to TSS distance (R^2=0.008, p-value 3.23×10^{-4}). The black lines represent the linear fit.

Supplementary Figure S4: Tissue specificity of pcRNA expression. A,B: Heatmap showing the Euclidean distance between the expression profiles of human (A) and mouse (B) pcRNAs. The vertical sidebar reports the tissues in which each pcRNA has maximal expression. The horizontal side bar reports the tissue specificity score of human pcRNAs. **C:** Bar chart showing the number of pcRNAs (y-axis) detected to have the highest expression in each given tissue (x-axis). **D**: Density distribution of the Tissue Specificity Score (see Supplementary Methods) for pcRNAs (blue) and pcRNA-associated coding genes (red) showing significant higher specificity for pcRNAs (mean pcRNA tissue specificity score 0.55, mean associated coding gene tissue specificity score 0.37, p-value 4.25×10^{-220}, Wilcoxon test). **E,F** pcRNAs display higher tissue specificity than the associated coding genes even when taking into account their lower expression levels. **E:** Scatterplot showing the highest FPKM observed across tissues (x-axis) for pcRNAs (blue) and pcRNA-associated coding genes (red) plotted against their tissue specificity score (y-axis). **F:** Scatterplot and density distribution of Tissue Specificity Scores for pcRNAs (blue) and pcRNA-associated coding genes (red) divided into 5 expression sub-groups. Each of the five sub-plots only displays pcRNAs and coding genes with similar expression levels (see Supplementary Methods) and shows the highest FPKM observed across tissues (x-axis) plotted against their tissue specificity score (y-axis). The right part of the plot shows the distribution of tissue specificity scores for each sub-group, showing that pcRNAs have higher tissue specificity score then pcRNA-associated coding genes independently of their expression level.

Supplementary Figure S5: GO enrichment of tissue-specific pcRNAs A-D: GO enrichment analysis of coding genes associated to pcRNAs with expression specific for Brain (A), Heart (B), Liver (C), Testis (D). The x-axis shows the enrichment score, calculated as the number of pcRNA-associated genes in a given GO category divided by the total number of genes in the category. The size of the points indicates the absolute number of pcRNA-associated genes in the given GO category. The color-coding indicates the p-values.

Supplementary Figure S6: **pcRNA expression during differentiation of NT2 cells. A**: Real time PCR data showing the expression of HOXB6 (blue) and HOXB5/6AS (red) in a panel of 7 human somatic tissues. The data is expressed relative to the expression of GAPDH; the error bars indicate the standard error of the mean (SEM) across 3 technical replicate experiments. **B-G**: Real time PCR data showing the expression of *HOXB6*, *SOX2, EVX1, HOXA5, TBX2, NR2F1* (left) and associated pcRNAs (right) over time-points of NT2 cells differentiation with retinoic acid (RA). The data is

expressed relative to the expression of *B2M*; the error bars indicate the standard error of the mean (SEM) across three (B*)* or two (C-G) replicate experiments.

Supplementary Figure S7: Expression clustering of Nanostring data A: Heatmap showing the pairwise Pearson correlation coefficients between all human transcripts included in the Nanostring experiment (both pcRNAs and pcRNA-associated coding genes). **B:** Network displaying all human transcripts included in the Nanostring experiment (nodes) and the Pearson correlation coefficient between their expression profiles (edges). Only edges with correlation coefficient higher than 0.5 are shown. The color coding of the nodes indicates the result of applying the Markov Clustering Algorithm to the matrix of correlation coefficients (see Supplementary Methods).

Supplementary Figure S8: Analysis of Nanostring data A: Top: Illustration of the *HNF1A* locus modified from a screenshot of the UCSC genome browser. For clarity, only one representative isoform of the coding gene is displayed. Middle**:** Nanostring expression profiles of *HNF1A* and associated pcRNAs across human (left) and mouse (right) tissues. The plots report the mean value of two technical replicates, while the error bars report the value of each replicate. Bottom**:** Heatmap showing Spearman's correlation coefficients between human and mouse *HNF1A*, *HNF1A–BT1* and *HNF1A–BT2 (*left*)* Real Time PCR data (right) showing the expression of *HNF1A* and *HNF1A–BT* in HepG2 cells upon knock-down of *HNF1A–BT*. Sh1- and sh2- HNF1A–BT indicate two different, non-overlapping shRNAs designed against *HNF1A–BT*. The data is expressed relative to the expression of the control transfected with scrambled shRNAs; the error bars indicate the SEM across two replicate experiments. **B,C: (**Top) Illustrations of the *SETD1B* and *FOXD2* loci modified from a screenshot of the UCSC genome browser. For clarity, only one representative isoform of the coding genes is displayed. (Bottom) Real Time PCR data showing the expression of *SETD1B-BT* and *FOXD2* -AS in MCF7 and K562 cells respectively upon knock-down of the relative coding genes or tapRNAs. Sh1- and sh2- indicate two different, non-overlapping shRNAs. The data is expressed relative to the expression of the control transfected with scrambled shRNAs; the error bars indicate the SEM across two replicate experiments.

Supplementary Figure S9: Histone modifications of pcRNA promoters. A-D: Histone modification profiles of pcRNA promoters (split by their relative orientation), promoters of 1000 random Gencode lncRNAs and promoters of 1000 random Gencode coding genes based on ChIP-Seq data by the ENCODE project on H1-hESCs (**A**), GM12878 **(B**), HSMM (**C**) and K562 (**D**). The lines represent the mean ChIP-Seq coverage and the shaded area around the line represents the standard deviation of the mean.

Supplementary Figure S10: H3K27me3 profiles of pcRNA promoters A-D: We identified an embryonic stem cell specific signature of pcRNA promoters with high levels of both H3K27me3 and H3K4me3 (bivalent promoters) or high levels of tri-methylation of H3K27 (H3K27me3) and intermediate levels of H3K4me3 (**A,B**). The pcRNAs clustered in these groups show intermediate

level or no expression in ES cells, respectively (**C,D**). Whereas both clusters are associated with developmental genes, the bivalent cluster is particularly enriched in central nervous system development (**E-H**). These results suggest these pcRNAs are targets of Polycomb and silenced or transcriptionally poised in undifferentiated pluripotent cells [3] consistent with roles in differentiation and development. **A**: Boxplot showing the mean coverage of pcRNA promoters based on ChIP-Seq signal for H3K27me3 and H3K4me3 in GM12878, H1-hESC, HSMM and K562. **B**: Scatter plot reporting the signal intensities of H3K4me3 (x-axis) and H3K27me3 (y-axis) in the promoters of pcRNAs. The four subplots represent data from H1-hESCs, GM12878, HSMM and K562. The colour coding reports the hierarchical clustering results. A single pcRNA had 0 H3K4me3 signal in H1hESCs and fell alone in a fifth cluster (not shown). **C**: Boxplot showing the expression (log10 FPKM) of pcRNAs based on RNA-Seq data on ES cells (left total cells; middle, cytoplasm; right, nucleus) and split by the cluster determined by applying hierarchical clustering to the H3K27me3 and H3K4me3 ChIP-Seq data (See Supplementary Methods). **D**: Histograms showing the number of expressed pcRNAs based on RNA-Seq data on ES cells (left total cells; middle, cytoplasm; right, nucleus) and split by the cluster determined by applying hierarchical clustering to the H3K27me3 and H3K4me3 ChIP-Seq data (See Supplementary Methods). pcRNAs with FPKM higher than 0.1 were considered expressed. **E-H:** GO enrichment analysis of coding genes associated to pcRNAs in each of the clusters determined by applying hierarchical clustering to the H3K27me3 and H3K4me3 ChIP-Seq data (See Supplementary Methods). The x-axis shows the enrichment score, calculated as the number of pcRNA-associated genes in a given GO category divided by the total number of genes in the category. The size of the points indicates the absolute number of pcRNA-associated genes in the given GO category. The color-coding indicates the adjusted p-value.

Supplementary Figure S11: Hi-C contact matrices of pcRNA promoters. Examples of HiC heat-maps, showing TADs (blue rectangles), loop anchor points (yellow dots), and tapRNA positions (black dotted lines). HiC data from GM12878 cells [4], with the first, second and third tracks corresponding to RefSeq coding genes, tapRNAs and CTCF ChiP-seq signal in GM12878, respectively.

Supplementary Figure S12: Identification and characterization of tapRNAs A: Bar chart showing the proportion of pcRNAs, pcRNA-associated coding genes, Gencode lncRNAs and Gencode coding genes with a TAD boundary overlapping their promoter. The p-values reported were calculated with hypergeometric tests. **B:** TAD boundary coverage of loci of pcRNAs, pcRNA-associated coding genes, Gencode lncRNAs and Gencode coding genes. The plots report the loci from 20kb upstream of the transcription start site (TSS) to 20kb downstream of the transcription end site (TES). For visualization purposes these profiles show the coverage of a random sample of 5000 Gencode lncRNAs and 5000 random Gencode coding genes **C:** HiC loops coverage of loci of pcRNAs subdivided by relative orientation. The plotted genomic regions encompass the loci from 20kb upstream of the TSS to 20kb downstream of the transcription end site (TES). **D**: Heatmap showing the proportion of each distal genomic region in contact with pcRNA promoter annotated in each genomic category derived from the ENCODE chromatin segmentation data (see Supplementary

Methods). **E**: Cumulative distribution plot showing the percentage of distal genomic regions in contact with pcRNA promoters (y-axis) as a function of the fraction of length of loop-end annotated as Transcript (left) or Other (right) according to the ENCODE chromatin segmentation data (see Supplementary Methods).

Supplementary Figure S13: Enriched motifs and binding sites in tapRNAs and loop anchor point.

A: Scatter plot showing transcription factor binding patterns (based on ENCODE ChIP-Seq data) at the proximal and distal anchor points of tapRNAs loops. The x-axis indicates, for each transcription factor, the fraction of tapRNA loops that it binds. The y-axis shows, for each transcription factor, the fraction of tapRNA distal anchor points that it binds. **B:** Significantly enriched 8-mer motifs in conserved domains. Probability density function of Monte Carlo simulation results is shown in bar graph. The motifs that have p-value <= 10^{-4} are considered as enriched motifs (shown in blue). The numbers on the enriched 8-mer motif stems are the consensus motif numbers as in **Figure 4D**. **C:** Enriched TF-binding motif in both conserved domain of tapRNA and enhancer region of loop anchor points. 32 significantly enriched 8-mer motifs (see **Supplementary Figure S13B**; p-value $1x10^{-4}$) in conserved domains in tapRNAs are identified and clustered into 10 consensus motifs. *De novo* motif analysis discovers known RBPs and TFs with matching binding consensus motifs. In both RBP and TF matching analyses, we identified that seven out of ten consensus motifs are part of binding motifs of zinc finger proteins. The other three consensus motifs are part of binding motifs of developmental regulatory proteins. The bars on the right show the extended DNA-binding motif search in enhancer regions of the other end of loop anchor points, finding significant enrichments of zinc finger protein motifs. **D:** Bar chart showing the proportion of tapRNAs and lncRNAs that are bound by CTCF observed by analysis of CLIP-Seq [5]. **E:** Bar chart showing the average number of CTCF CLIP-seq peaks in tapRNAs and lncRNAs.

Supplementary Figure S14: Visualisation of the FOXA2-DS-S locus. Screenshot from the Dalliance genome browser [6] showing the *FOXA2* locus with tracks displaying coverage data for several ChIP-Seq experiments performed by the ENCODE project on HepG2 cells.

Supplementary Figure S15: Expression analysis of pcRNA knock-downs A-D: Real time PCR data showing the expression of pcRNAs and associated coding genes upon knock-down of *FOXA2* (**A**), *NR2F1* (**B, C**), *POU3F3* (**D**) and their associated pcRNAs. **E-H:** Invasion and migration assay analysis upon knock-down of *FOXA2* (**E**), *NR2F1* (**F,G**), *POU3F3* (**H**) and their pcRNAs compared to negative control siRNA.

Supplementary Figure S16: Expression profiles of pcRNAs in cancer cell lines A: Heatmap showing the Nanostring expression profiles of human pcRNAs across all the cancer cell lines included in the assay. **B-E**: Nanostring expression profiles of human pcRNAs and genes at *HNF1A* (**B**), *FOXA2* (**C**), *POU3F3* (**D**) and *NR2F1* (**E**) loci across all the cancer cell lines included in the assay.

Supplementary Figure S17: Expression profiles of pcRNAs in cancer Microarray data and TCGA RNA-seq data A: Heatmap showing pcRNAs differentially expressed in cancer microarray studies. Student t-test (p-value < 0.005 and fold-change > 1.25) was used to identify pcRNAs (rows) that were up (red) or down-regulated (blue) in tumors compared to normal tissues (rows) (see Supplementary Table S6). Examples of pcRNAs associated with specific loci are shown. **B:** Heatmap showing pcRNAs differentially expressed in TCGA RNA-seq V2 Level3 data.

Supplementary Figure S18: Expression profiles of tapRNAs and their associated coding genes in TCGA RNA-seq data **A**: Heatmap showing tapRNAs differentially expressed in TCGA RNA-seq V2 Level3 data. **B**: Heatmap showing tapRNAs-associated coding genes differentially expressed in TCGA RNA-seq V2 Level3 data.

Supplementary Table Legends

Supplementary Table S1: List of RNA-Seq and ChIP-Seq datasets used in the study.

Supplementary Table S2: Annotation of pcRNAs.

Supplementary Table S3: GO enrichment of pcRNA-associated protein coding genes.

Supplementary Table S4: GO enrichment protein coding genes associated with pcRNAs in each possible orientation.

Supplementary Table S5: Nanostring data. **A**: Annotation of probes used in the assay. **B:** List of samples tested in the assay. **C:** Normalised expression of tested human and mouse pcRNAs and associated coding genes.

Supplementary Table S6: Metanalysis of pcRNA expression across different cancer studies. **A:** Samples used in the meta-analysis. **B:** Expression of pcRNAs. **C:** Expression of pcRNA-associated coding genes. **D:** Summary table with the number of expressed pcRNAs and coding genes in each study.

Supplementary Table S7: List of tapRNAs with mutated CTCF and/or ZNF263 sites.

Supplementary Table S8: Oligonucleotides, clones and cell lines used in this study.

Supplementary Methods

Human and Mouse reference genomes

The reference genomes for human (hg38) and mouse (mm10) were downloaded from the UCSC FTP server [7] in 2bit format and converted to fasta format using the twoBitToFa tool from the UCSC genome browser. The fasta files were indexed using samtools faidx (v1.2).
The Bowtie index for both genomes were built with bowtie2-build (v2.1.0) [8].

Human and Mouse reference transcriptomes

The reference gencode transcriptomes for human and mouse (version 21 for human and version M4 for mouse) were obtained from the Gencode website in GTF format [9].

Genbank all RNAs

The annotation of mouse Genbank mRNAs was obtained from the "Mouse mRNAs from GenBank" track of the UCSC genome browser using the Table Browser [10].

RNA-Sequencing data analysis

In order to obtain comprehensive transcriptomes for human and mouse as well as to quantify pcRNA abundance, we integrated the reference Gencode transcriptomes with RNA-Seq data on human and mouse tissues and cell lines. We used RNA-Seq from six matched human and mouse tissues (Brain, Cerebellum, Heart, Kidney, Liver and Testis) as well as data produced by the ENCODE project from similar human and mouse cell lines (**Supplementary Table S1**).

Mapping

The RNA-Seq datasets were mapped to the reference human and mouse genomes (hg38 and mm10 respectively) using Tophat2 (v2.0.10, bowtie2 v2.1.0 [11]) with the options "--b2-sensitive --zpacker pigz" and the Gencode comprehensive GTF files as reference transcriptomes (v21 and M4 for human and mouse respectively). The reference transcriptomes were built with an independent Tophat run without fastq files and then provided to all subsequent mapping runs through the option "--transcriptome-index"
The --library-type option was set to "fr-unstranded" for unstranded datasets and "fr-firststrand" for stranded datasets.
For two very deep (>120mln reads each), single end 45nt reads mouse datasets (SRR549335 and SRR549339, see Supplementary Table S1) Tophat by default tried to identify splice junction by coverage search, but stopped at the stage "Searching for junctions via segment mapping" probably due to the very high number of reads. To overcome this problem, we disabled only for these two samples the coverage-search functionality (option --no-coverage-search) as suggested by Tophat's standard error.

Assembly

The transcriptomes were assembled independently for each RNA-Seq dataset using Cufflinks (v2.2.1). Cufflinks was configured to perform Reference Annotation Based Transcript assembly (RABT) to avoid assembling incomplete transcript models of known transcripts (-g option). For each gene, transcripts with abundance less than 5% of the most abundant transcript were discarded (-F option).
Cufflinks was started with the following options:

- **--library-type** "fr-unstranded" for unstranded datasets and "fr-firststrand" for stranded datasets.
- **-F** 0.05
- **--multi-read-correct**
- **--frag-bias-correct**, pointing to the genome fasta file
- **-M** a masking GTF files to exclude ribosomal transcripts and mitochondrial transcripts. This file was produced by selecting from the Gencode GTF files the lines that matched "Mt_" or "rRNA" in field 14.
- **-g** exon-cds-filtered reference transcriptome GTF. This file was produced by selecting only exon and CDS features from the Gencode reference GTF files (field 3), therefore excluding the "gene" and "transcript" entries. Such a filtered GTF file contains all the information needed by Cufflinks and provides a significant speed up in cufflinks' running time.

The Cufflinks assembled transcriptomes for each sample were then merged using Cuffmerge (with the same exon-cds reference transcriptome used for Cufflinks) and converted to BED12+ format using the gtfToBed tool [12] with the option “-a gene_id,oId,class_code” to preserve Gene ID, Gencode ID and Cufflinks class codes as additional fields.

Abundance estimation and expression normalisation

The human and mouse merged transcriptomes (merged.gtf) were then quantified against each BAM file using Cuffquant (v2.2.1) with the following options:
--library-type (see Cufflinks)
--multi-read-correct
--frag-bias-correct
-M Reference masked regions (see Cufflinks)
Finally, the Cuffquant binary output files were normalised with Cuffnorm (v.2.2.1) to produce the human and mouse expression matrices. Cuffnorm was run with the following options:
--output-format cuffdiff
--use-sample-sheet
--library-type fr-unstranded

Identification of pcRNAs

Human Data preparation

The purpose of this data preparation step is to produce an annotation of reference and novel non-coding transcripts from which we will later identify pcRNAs.

1) Annotation of coding transcripts and CDS
From the Gencode BED annotation we selected transcripts containing an annotated CDS. We then used the getCoding tool of Pinstripe [13] to obtain a BED annotation of only the coding portion (CDS) of each coding transcript.

2) Reference non coding RNAs
We filtered the Gencode V21 BED file in the following way:

1. We used awk to select all transcripts without an annotated ORF and composed of more than one exon.
2. We used overlapSelect (UCSC genome browser tool [12]) to exclude all transcripts that had more than 20bp of sense overlap with the CDS region of a coding transcript.

3) Novel non coding RNAs
We filtered the merged RNA-Seq transcriptome BED file in the following way:

1. We used awk to remove single exon transcripts as well as transcripts that don't map to the primary assemblies of the autosomes or sex chromosomes.
2. We used overlapSelect to discard transcripts with more than 20bp of sense overlap with the CDS region of a coding transcript.
3. We used bedtools intersect (v2.24.0) to discard transcripts with more than 50% sense exonic overlap with reference non-coding transcripts (previous step).
4. We used Pinstripe dedup (version v1.0.4554.32000, with option --exEncomp) to remove redundant transcripts.
5. We used CPAT (v1.2 [14]) to calculate the coding potential of each transcript and only retained transcripts with score <0.364 (see CPAT documentation for information on the threshold).

Finally, we combined *the Reference non coding RNA* annotation and the *Novel non coding RNAs* annotation and used bedtools intersect to remove all transcripts with more than 50% sense exonic overlap with coding transcripts (although in the previous step we had already filtered out CDS-overlapping transcripts, this step ensures that we do not have transcripts that have more than 50% overlap with the UTR of coding genes).
The file that we obtain is a comprehensive annotation of all reference and novel human non-coding RNAs and we will hereafter refer to it as *know+novel ncRNAs*.

Mouse Data preparation

The purpose of this data preparation step is to produce an annotation of reference and novel non-coding transcripts from which we will later identify pcRNAs.

1) Annotation of coding transcripts and CDS
From the Gencode BED annotation we selected transcripts containing an annotated CDS. We then used the getCoding tool of Pinstripe to obtain a BED annotation of only the coding portion (CDS) of each coding transcript.

2) Reference non coding RNAs
We filtered the Gencode M4 BED file in the following way:

1. We used awk to select all transcripts without an annotated ORF and composed of more than one exon.
2. We used overlapSelect to exclude all transcripts that had more than 20bp of sense overlap with the CDS region of a coding transcript.

3) Novel non coding RNAs
We filtered the merged RNA-Seq transcriptome BED file in the following way:

1. We used awk to remove single exon transcripts as well as transcripts that don't map to the primary assemblies of the autosomes or sex chromosomes.

2. We used overlapSelect to discard transcripts with more than 20bp of sense overlap with the CDS region of a coding transcript.
3. We used bedtools intersect (to discard transcripts with more than 50% sense exonic overlap with reference transcripts (previous step)
4. We used Pinstripe dedup (with option --exEncomp) to remove redundant transcripts
5. We used CPAT to calculate the coding potential of each transcript and only retained transcripts with score <0.44 (see CPAT documentation for information on the threshold).
6. We removed all transcripts with more than 50% sense exonic overlap with a coding transcript to remove UTR overlapping RNAs.

4) *Genbank non coding RNAs*
Given the lower number of lncRNAs annotated by Gencode in mouse (6951 in Gencode M4 vs 15877 in human Gencode V21), we also incorporated in our analysis Genbank non coding RNAs.
To identify them, we downloaded the "all mRNAs" GTF from the UCSC genome browser and processed it in the following way:

1. We used the gffread tool (v2.2.1, part of the Cufflinks suite) to exclude all transcripts with non-canonical splice sites (i.e. not GT-AG, GC-AG or AT-AC) and with introns shorter than 4nt, then we converted the filtered GTF file to BED with Pinstripe gtfToBed.
2. We retained only transcripts with more than one exon.
3. We discarded transcripts with more than 20bp of sense overlap with the CDS region of a coding transcript (overlapSelect).
4. We used CPAT to calculate the coding potential of each transcript and only retained those with score <0.44 (see CPAT documentation for information on the threshold).
5. We removed all transcripts with more than 50% sense exonic overlap with a coding transcript to remove UTR overlapping RNAs.

Identification of conserved promoters

For each transcript in the human *know+novel ncRNAs* annotation (see Human Data preparation section) we produced a BED file of their promoter regions by extending their TSSs of 500bp in each direction, then we obtained their FASTA sequence from the reference genome using the Pinstripe getDna tool.
In order to make a blast database of the mouse genome we first used the ncbi-blast convert2blastmask tool (v2.2.30+, options *-masking_algorithm repeat -masking_options "repeatmasker and tandem repeats from UCSC" -outfmt maskinfo_asn1_bin*) to extract masking information from the soft masked genome fasta file, and then the makeblastdb tool (v2.2.30+, *options -mask_data path-to-convert2blastmask –out -dbtype nucl*).
We then used the following command line to align human ncRNA promoters to the mouse genome with blast (v2.2.30+):

```
blastn -task blastn –db path/to/db -out path/to/out  -query path/to/promoters/fasta -outfmt 6 -evalue 0.001 -num_threads n-processors -db_soft_mask 40 -lcase_masking
```

Finally, we processed the blastn output file with awk to only retain alignments longer 100nt and with E-value <10^{-10} and convert the blast coordinates (1 based) into BED coordinates (0 based).

Non-coding to coding positional annotation

We next aimed to associate each ncRNA identified in human and mouse to its closest protein coding transcripts. To this end we used Pinstripe "closest", which returned – for each input ncRNA – the closest upstream, downstream and overlapping coding transcript. We then processed each entry and compared the non-coding and coding coordinates to annotate their TSS-to-TSS distance as well as the orientation of the non-coding relative to the coding in the following way:

- If the coding and non-coding intervals overlapped we defined the coding-non-coding pair as OLAP if on the same strand or AS if on different strands.
- If there was no overlap and the non-coding was upstream of the coding (relative to the strand of the coding), we defined the pair as US-S if coding and non-coding were on the same strand, otherwise US-AS if the TSS-to-TSS distance was >2000bp or BT if <=2000.
- If there was no overlap and the non-coding was downstream of the coding (relative to the strand of the coding), we defined the pair as DS-S S if coding and non-coding were on the same strand, otherwise DS-AS.

We then matched each human and mouse coding transcript to their corresponding Ensembl Gene Ids, and for each non-coding/coding gene pair in a given orientation we only retained the closest coding transcript.

Human-mouse positional comparison

To identify mouse ncRNAs arising from conserved human ncRNA promoters we extended each region in the mouse genome that resulted from blasting human ncRNA promoter (see "Identification of conserved promoters") of 500nt in each direction, and then we intersected these regions with the 5' exon of each mouse ncRNA.

This step allowed us to obtain pairs of human/mouse ncRNAs that have a conserved promoter. We then selected those pairs for which at least one coding neighbour of the human non-coding (where neighbouring means the closest upstream, downstream and overlapping as defined in the previous step) was orthologous to at least one coding neighbour of the mouse non-coding

To identify orthologous genes between mouse and human we programmatically downloaded from Ensembl Biomart (v80) a table that associates each human gene_id to the gene_id of the orthologous gene in mouse.

The resulting annotation contains human and mouse ncRNAs whose promoter is conserved and whose neighbouring gene(s) is(are) orthologous.

We further filtered this annotation by removing all human/mouse ncRNA pairs that were in opposite orientations relative to the coding genes in the two species (i.e. DS-S, US-S or OLAP in one species and AS, BT, DS-AS, US-AS in the other).

In numerous cases we could not univocally associate each ncRNA to a single coding gene, since the same ncRNA can have multiple neighbouring coding genes orthologous and in the same orientation in mouse and human. To resolve these ambiguities and univocally assign each ncRNA to a unique coding gene we applied the following criteria:

1) In case any of the possible coding genes were either AS or OLAP in human we retained the closest (TSS-to-TSS) of those.
2) In all other cases we retained the coding with shortest TSS-to-TSS distance in human.

Annotation of pcRNA genomic characteristics

To annotate pcRNAs that overlapped Gencode lncRNAs we intersected the human pcRNA annotation with the Gencode annotation of lncRNAs considering all exonic sense overlaps.

To annotate pcRNAs that overlapped miRNAs we queried the UCSC genome browser MySQL server for all transcripts containing the string "miR" in the geneName field.

To annotate pcRNA promoters we extended each pcRNA TSS by 2000bp in each direction and merged the resulting promoter regions that overlapped (bedtools mergeBed).

Characterisation of pcRNA features and expression analysis

To produce human and mouse expression matrices we matched the Ensemble Transcript IDs of human and mouse pcRNAs with the “oID” identifiers reported by Cuffmerge; we then used the corresponding Cuffmerge IDs to track pcRNAs in the isoforms FPKM tracking files reported by Cuffnorm.
For human and mouse coding genes we used a similar approach to extract the FPKM transcript information for all transcripts of each coding gene, and then summed the FPKMs to obtain a single expression measure at the gene level.
For the expression analysis all FPKM values below 10^{-3} were set 0 and all transcripts with 0 FPKMs in all samples were excluded.

pcRNA expression heatmaps

The expression heatmaps for human and mouse pcRNAs were produced with the function heatmap.2 of the gplots package. The rows and columns were clustered with the default methods. For visualisation purposes in order to calculate the log2 of the FPKMs the smallest FPKM value was added to each value. The vertical sidebar reports the tissue specificity score calculated as indicate below.

pcRNA expression distance heatmaps

The heatmaps showing the Euclidean distance between pcRNA expression profiles have been realized by calculating the matrix of pairwise Euclidean distances between all pcRNAs using the *dist()* function in R. The heatmap was produced with the heatmap.2 function of the gplots package using the default methods for clustering rows and columns. The horizontal sidebar reports the tissue specificity score calculated as indicate below. The vertical sidebar reports the tissue where a given pcRNA has maximal expression.

GO enrichment of pcRNA-associated coding genes

The GO enrichment of pcRNA-associated genes was obtained using the TopGO package of Bioconductor. The ontology mapping used was provided by the package org.Hs.eg.db. The background set of coding genes consisted of all human protein coding genes with an annotated mouse ortholog. GO nodes with less than 10 annotated terms were excluded from the analysis. The p-values were calculated using the “default” method of TopGO and Fisher’s Exact test. P-values were corrected using the Benjamini-Hochberg method as implemented in the *p.adjust(method="BH")* function in R. For the GO enrichments of pcRNA-associated genes divided by relative orientation, we used as background the set of all pcRNA-associated coding genes. P-values were calculated as described above but were not corrected for multiple hypothesis testing.
To control for the intergenic-distance distribution of pcRNAs-associated coding genes, we first annotated the “intergenic island” size for each Gencode gene: we first selected the longest transcript for each gene, and then used bedTools *closestBed* to measure the distance of the closest upstream and downstream genes. We defined the sum of the upstream and downstream distances as the island size, and then used the matchit function of the MatchIt R package (ratio=5, method="nearest") to subsample a control group of Gencode protein coding genes (5 times as big as the group of pcRNA associated coding genes) that has the same distribution of island size as pcRNA associated coding genes. This group of genes was then used as the background set for the GO enrichment analysis as described above.

Correlation of expression between pcRNAs and coding genes and between human and mouse pcRNAs

To calculate the Spearman’s rank correlation coefficients between human pcRNAs and coding genes we first calculated a matrix of coefficients between each pcRNA and each coding gene, where the diagonal represented the coefficients between each pcRNA and its associated coding gene.
To test whether the mean correlation coefficient was higher than expected by chance we performed a permutation test: we selected 10^6 samples of random coefficients from the entire matrix, and calculated how many times the mean of the random sample was higher or equal to the mean of the diagonal of the matrix. We reported a p-value<10^6 when none of the random samples’ means was higher or equal to the mean of the diagonal.
The correlation coefficients were calculated in R with the function *cor(method=”spearman”)*.

The correlation of expression between human and mouse pcRNAs was calculated in the same way. When the same human pcRNA was associated to multiple mouse pcRNAs we calculated the correlation between all pairs.

Tissue specificity score and GO enrichment by tissue

The tissue specificity score for human and mouse pcRNAs and coding genes was based on the square root of the Jensen-Shannon divergence as in [15]. The p-value for the difference between pcRNAs and coding genes was calculated with the Wilcoxon test as implemented in the wilcox.test function in R. To verify whether the difference of tissue specificity score between pcRNAs and coding genes was due to their different expression levels, we used the MatchIt R package (*method="subclass", subclass=5, sub.by="control"*) to subdivide pcRNAs and coding genes in 5 classes so that each class had similar distributions of maximal FPKM. We then calculated the tissue specificity score distribution for each of the 5 classes.
The GO enrichment of pcRNA by tissue of maximal expression was done by selecting the coding genes associated with pcRNAs with maximal expression in the given tissue and with a tissue specificity score above the mean of all specificity scores. The GO enrichment was performed in R using the TopGO package. The background set of coding genes consisted of all pcRNA-associated coding genes. GO nodes with less than 20 annotated terms were excluded from the analysis. The p-values were calculated using the “default” method of TopGO and Fisher’s Exact test. P-values were not corrected for multiple hypothesis testing.

Human-mouse conservation analysis

To calculate the sequence conservation of human and mouse pcRNAs, we aligned the human and mouse pcRNA sequences using the Needleman–Wunsch algorithm (Needleman and Wunsch, 1970). In case multiple mouse pcRNA isoforms were associated with the same human pcRNAs we performed all possible pairwise alignments and only retained those with the highest sequence identity. Similarly, to calculate the sequence conservation of pcRNA-associated protein coding genes we performed pairwise alignments (Needleman–Wunsch algorithm) between all transcripts of the human gene and all transcripts of the mouse gene, and retained the alignment with the highest sequence identity.

CAGE analysis

The FANTOM5 CAGE data was downloaded in hg19 coordinates from the FANTOM portal (cage_peak_phase1and2combined_coord.bed) and converted to hg38 coordinates using *liftOver*. We then used bedTools *closestBed* to identify the closest CAGE tag to the TSS of each pcRNA and calculate its distance. The cumulative distribution of the distances was calculated in R and plotted with ggplot2.

Splice sites conservation profiles

For each pcRNA we extracted the annotation of their first, internal and last exons. We then used deepTools *computeMatrix* to calculate the average phastCons100way score (as downloaded from UCSC) for each non-overlapping bin of size 10bp spanning the length of each exon (scaled to a uniform length of 10kb) as well as the region of 1kb downstream or upstream the splicing donors and acceptors respectively. We then calculated the average of each bin in R and plotted it with ggplot2.

Subcellular localisation analysis

Gene expression data from subcellular fractions was downloaded from the lncATLAS database (ref). The atlas data in the database is quantified at the gene level, therefore for subsequent analysis we discarded all pcRNAs that are annotated as ‘protein_coding’ in Gencode (i.e. pcRNAs which are annotated as non-coding isoforms of a protein coding gene). After this filtering step, 386 pcRNA genes were retained. The database annotates each gene as either ‘coding’ or ‘nc’, therefore we used the Gencode annotation to further subdivide the ‘nc’ class into Gencode lincRNAs, pcRNAs and other ncRNAs. We then filtered the database to only retain genes with FPKM>0 in whole cells and FPKM>0 in either nucleus or cytosol and calculated the inverse logit function of the Relative Concentration Index. Since the database reports expression levels in multiple cell types, for each gene we only retained the cell type where the expression is highest in the whole cells. We then used the R function matchit (MatchIt package, method=”nearest”, subclass=3, sub.by=”treat”) to partition the data into 3 subclasses of matched expression in whole cells. We then compared the distribution of the RCI between pcRNAs and lincRNAs in each subclass.

Identification of pcRNAs targeted by CRISPR-interference (CRISPRi)

To identify pcRNAs with potential roles in cellular growth, we used bedtools intersect to annotate pcRNA promoters (+/- 500bp) overlapping with coordinates of sgRNAs targeting 16,401 lncRNA promoters in seven cell lines and resulting in a significant growth defect (either 'lncRNA hit' or 'Neighbor hit') [16]. We identified 202 pcRNAs are in the 916 growth defected lncRNA list. To test whether pcRNAs have significant enrichment, we performed a hypergeometric test as implemented in the phyper function in R.

Identification of Pfam domains in pcRNAs

The sequence of pcRNAs was translated into all possible ORFs (all frames) using transeq (EMBOSS suite). The resulting amino acid sequences where then scanned against the Pfam database (v31.0) using pfamScan (v1.6) with default parameters. All pcRNAs with significant hits where then annotated in Supplementary Table S2.

Nanostring analysis

For the nanostring experiment we designed probes to detect 50 pairs of pcRNAs and corresponding coding genes in human and mouse. The probes were designed according to the Nanostring guidelines and to maximize their specificity (Supplementary Table S5) and included 9 house-keeping genes for normalization (*ALAS1, B2M, CLTC, GAPDH, GUSB, HPRT, PGK1, TDB, TUBB*).

The raw count data were first normalized by Nanostring Technologies with the nSolver software using a two-step protocol. First, data were normalized to internal positive controls, then to the geometric mean of house-keeping genes. The normalised data was then imported into R for further analysis. The correlation of expression between pcRNAs and coding genes was calculated with the *cor()* function in R after averaging replicate samples. To test whether the mean correlation coefficient between pcRNAs and coding gene as well as between human and mouse pcRNA pairs was higher than expected by chance, we used a permutation test as described for the RNA-Seq analysis.

To cluster pcRNAs and coding genes based on their expression profiles with first used the mcxarray tool of MCL [17] to produce a graph where nodes represented human pcRNAs and corresponding coding genes, and edges connected nodes with a Pearson correlation coefficients higher than 0.5. We then run MCL on such graph with the inflation parameter set to 3 to identify clusters of pcRNAs and coding genes.

FOXA2-DS-S knock-down microarray analysis

RNA samples were amplified using the TotalPrep 96-RNA amplification kit from Ambion (Applied Biosystems). Briefly, the RNA was converted into cDNA, and amplified by In Vitro Transcription (IVT) to generate biotin-labeled cRNA. The cRNA was then hybridized to the *HumanHT-12 Expression Chips, version 4* following the Direct Hybridization assay.

The data obtained was imported into R and analyzed with the beadarray Bioconductor package [18] and the illuminaHumanv4.db annotation package. We summarized the data for each array using the *summarize()* function of beadarray with default parameters (log2 transformation, removal of outliers with a 3 median absolute deviation cutoff) and removed all probes without a quality score or with a “Bad” quality score in the annotation package. We then normalized the data with the *normaliseIllumina()* function with the quantile method and retrieved Ensembl IDs for each array probe using biomaRt. We then performed the differential expression analysis using limma with the model formula ~0+Condition, where Condition identifies the Control samples, FOXA2-KD samples and FOXA2-DS-S samples. We also supplied to the *lmFit()* function a weight for each array estimated using the *arrayWeights()* function. The GO enrichment analysis was performed separately on significantly up-regulated (adjusted p-value<0.05 and log2 fold change>0) and down-regulated (adjusted p-value<0.05 and log2 fold change<0) genes using the TopGO package. As background set we used all probes in the array with an Ensembl gene id. The GO enrichment was performed with the classic algorithm and p-values calculated with the fishes exact test. P-values were corrected for multiple hypothesis testing using the Benjamini-Hochberg method as implemented in the *p.adjust()* function. All GO terms with less than 20 annotated genes were excluded from the analysis.

pcRNA histone modification profiles

To produce histone modification maps of pcRNA promoters we downloaded the normalised bigWig files from the EBI ENCODE repository for 14 ChIP-Seq experiments (Control, Ctcf, H2az, H3k27ac, H3k27me3, H3k36me3, H3k4me1, H3k4me2, H3k4me3, H3k79me2, H3k9ac, H3k9me1, H3k9me3, H4k20me1) in GM12878, H1-hESC, HSMM and K562 cells. We then converted the data to bedGraph format (with bigWigToBedGraph) and used liftOver to convert the coordinates from hg19 to hg38. Then, we used bedtools mergeBed to merge the overlapping intervals and converted the resulting files back to bigWig format (bedGraphToBigWig).

For each cell line we then used the computeMatrix of the Deeptools package (reference point TSS) to calculate the coverage in each ChIP-Seq experiment of each pcRNA as well as of 100 random Gencode lncRNAs and 100 random Gencode coding Genes (random coding genes and lncRNAs selected with bedtools sample with seed 383847). The resulting matrix was then loaded in R to produced the profile plots.

Analysis of H3K27me3 in ESCs

To study the H3K27me3 profiles of pcRNA promoters, the bedGraph files (in hg38 coordinates, see above for details of conversion from hg19) of H3K27me3 and H3K4me3 in GM12878, H1-hESC, HSMM and K562 have been mapped to the promoters of pcRNAs (defined as TSS +/- 1Kb) using the mapBed tool of bedtools to calculate the mean coverage of each promoter in each cell line. The data was then loaded into R and the data for H1-hESC was subjected to hierarchical clustering using the hclust function (default parameters) on the Euclidean distances matrix (dist function) between the base 10 logarithms of the mean promoter coverage for H3K27me3 and H3K4me3 (the log10 was calculated after adding 0.01 to each value). The GO enrichment for the coding genes associated to the pcRNAs in each cluster was performed using TopGO (classic algorithm) using the Fisher Exact Test to compute p-values. P-values correction and background set were the same as described for "*GO enrichment of pcRNA-associated coding genes*".

ENCODE TF ChIP-seq data analysis

We downloaded 2,216 ChIP-seq experiment data from the ENCODE Project. The list of the data is in Supplementary Table S8. The data were lifted over from hg19 to hg38. We found overlapping peaks on four different categories: (1) 500bp upstream the promoter region of pcRNA-associated coding genes, (2) 500bp upstream promoter region of pcRNAs, (3) pcRNA genomic loci, and (4) pcRNA genomic loci but not overlapping with promoter region. To understand the correlation of TF binding patterns in the four categories, we made a binary matrix per category that consists of rows of TFs and columns of pcRNA/coding genes. Hence, the matrix contains connections between TF and pcRNA/associate coding genes. The matrix of category 2 is clustered by Euclidian Distance. To check the extent to which promoter sharing or proximity determines TFBS correlation, we also separated the clustered heat-map in the pcRNA bidirectional transcript (BIDIR) subgroup to the other subgroups (Non-BIDIR). To directly compare the TF binding patterns between each category, the other three matrices were sorted by the same order of the clustered matrix. We used the MatLab function *corr2* to calculate r-value between category (1) and (2). We performed Monte Carlo simulation to calculate the p-value and test the significance of the r-value.

Known TF-binding motif data analysis

We downloaded known TF-binding motifs from JASPAR database [19] (freeze 2014-12-10, 263 motifs) [20] (2,065 motifs) and [21] (843 motifs). We applied same analytical procedures on these datasets as described in previous section (ENCODE TF ChIP-seq data analysis).

Identification of CTCF binding sites in pcRNA promoters

To identify CTCF binding sites within pcRNA promoters we downloaded the TFBS clusters (V3) from the ENCODE portal at the UCSC Genome Browser (wgEncodeRegTfbsClusteredWithCellsV3.bed.gz) and filtered the file for CTCF sites. We then converted the CTCF binding sites to hg38 coordinates using the liftOver tool and calculated how many pcRNA promoters overlapped CTCF sites by (1) extending the TSS of each pcRNA by 2kb in both directions, (2) merging overlapping promoters (bedtools merge) and (3) intersecting the promoters with the CTCF sites. We repeated the same procedure for pcRNA-associated genes, Gencode coding genes and Gencode lncRNAs. To test whether pcRNA promoters were significantly enriched in CTCF binding sites compared to Gencode lncRNAs, we performed a hypergeometric test as implemented in the phyper function in R.

Identification of HiC loops that overlap pcRNAs

We obtained the annotation of HiC loops by downloading the loops list files for HMEC, HUVEC, NHEK, K562, HeLa, KBM7, IMR90 and GM12878 cells deposited on GEO (GSE63525) [4] and converted the intervals into Hg38 coordinates using liftOver. To calculate how many pcRNA promoters overlap HiC loops, we first extended the TSS of each pcRNA by 2kb in both directions, merged overlapping promoters (bedtools merge) and intersected the promoters with the loop coordinates. We also repeated the same procedure for all pcRNA-associated coding genes, Gencode coding genes, and Gencode lncRNAs. To test whether pcRNA promoters are significantly enriched in HiC peaks compared to Gencode lncRNAs we performed a hypergeometric test as implemented in the *phyper()* function in R.
We applied the same strategy to identify TAD boundaries overlapping pcRNA promoters. However, because TAD boundaries are single nucleotides rather then intervals, we extended each boundary 10kb in each direction.

To analyze the end-points of the loops that overlap pcRNA promoters we downloaded the ENCODE Broad HMM data [22] from the UCSC repository for GM12878, H1-hESC HEPG2, HMEC, HSMM, HUVEC, K562, NHEK and NHLF cells. After converting the coordinated to Hg38 with liftOver we intersected each HMM dataset with the coordinates of the end points of the loops that overlapped pcRNAs or – as a control – all Gencode lncRNAs.

The data were then loaded into R for further analysis. First, we simplified the data by reducing the number of HMM categories in the following way: Strong and Weak Enhancer categories were grouped as Enhancer; Active, Weak and Poised promoter were grouped as Promoter; Txn_Elongation, Txn_Transition and Weak_Txn were grouped as Transcript; everything else was grouped as Other.

Then, for each end-point in each cell line we calculated the fraction covered by each HMM category and plotted these data as a heatmap using the heatmap.2 function of the gplots package. To determine whether pcRNA-loop end-points were enriched in any specific HMM category we calculated for each HMM category *x* the fraction of end-points annotated as *x* for at least *y%* of their length, where *y* ranged from 1 to 0 in steps of 0.1. Finally, we compared this distribution to the distribution obtained for all Gencode lncRNAs using the Kolmogorov-Smirnov test (as implemented in the *ks.test()* function in R).

TAD/Loop Boundary Enrichment Analysis

To check whether pcRNAs are localized at the boundary of TAD/loop, we generated a density plot that shows cumulative counts of pcRNA appearance across TAD/loop regions (for each TAD including 10% proximity regions outside the TADs). First, we extended the TSS of each pcRNA by 2kb in both directions and merged overlapping promoters (bedtools merge). Second, we extended TAD regions by 10% in both directions. We then intersected the promoters with the loop coordinates and the extended TAD coordinates (bedtools intersect).

To visualize the cumulative count as a density plot, we only cumulated 10-bp window centered by TSS of each overlapping pcRNA to show precise localization of pcRNA TSS. We used all lncRNAs in the Gencode database (excluding pcRNAs) as a control. We then performed Kolmogorov-Smirnov test (as implemented in the kstest2 function in MatLab) to check the significance of the enrichment.

PhastCons Conservation Analysis

To understand the general conservation level of pcRNAs, we used phastCons scores for multiple alignments of 99 vertebrate genomes to the human genome. We downloaded wigFix format files from UCSC database to analyze at single base-pair resolution (http://hgdownload.cse.ucsc.edu/goldenPath/hg38/phastCons100way/hg38.100way.phastCons/). We fetched phastCons scores spanning the regions of pcRNA exons. The scores were summed up per pcRNA and then divided by the total length of exons per pcRNA. We also repeated the same procedure for Gencode coding genes and lncRNAs. We then used Kernel smoothing function estimate (as implemented in the ksdensity function in MatLab) to plot the density of the normalized phastCons scores per pcRNAs. To test whether pcRNAs are significantly more conserved than Gencode lncRNAs, we performed Kolmogorov-Smirnov test as implemented in the kstest2 function in MatLab.

Conserved domain search

To identify conserved domains, we aligned transcribed sequences of human pcRNAs against their counterpart mouse pcRNAs. We took two alignment approaches: sliding-window and exon-by-exon. For the first approach, we made a 200nt-long window on each human pcRNA sequence and shifted the window by 40nt to align against the whole length of the transcribed mouse pcRNA sequence. For the second approach, we took each exon of human pcRNAs and aligned them against the whole length of the transcribed mouse pcRNA sequences. In both approaches, we used MATLAB function localalign, which returns local optimal and suboptimal alignments between two sequences. We found highly concordant search results in the results of both approaches and further analyzed the alignments by applying the following steps: (1) retain alignments only if the alignment score is greater than 100 or the ratio of identical matches is greater than 80%, (2) remove duplicate alignments among isoforms of pcRNAs based on merged isoforms of pcRNAs list, (3) remove alignments if the aligned regions in human and mouse pcRNAs are not in same order of exons on their transcribed sequences, and (4) retain the best alignment if there are multiple alignments for the same region. Regarding the merged isoforms, we extracted only exonic regions of each pcRNA, and then merged the regions by using the bedtools merge function. The merged isoform allowed us to search once per a given genomic region that prevented multiple counting of same conserved domain and motif.

Through this process we generated a heatmap of conserved domains in human pcRNAs and clustered it by using the MATLAB function kmeans, which performs k-means clustering to partition the observations of a given matrix into k clusters. We used kmeans on the squared Euclidean distance measure and the k-means++ algorithm for cluster center initialization. We found 16 clusters and merged them into four larger clusters (**Figure 4A**). To annotate the functional category for each four clusters, we used DAVID functional annotation tool with default settings [23].

Motif search in conserved domains

To determine which regulatory motifs are over-represented in conserved domains with respect to background non-conserved regions, we identified all possible ungapped 8-mers in conserved domains and computed their frequency. An 8-mer is considered over-represented if its frequency in conserved domain is significantly higher than the frequency in background non-conserved region. In the list of over-represented motifs, we found the presence of repeats that are consisted of a single nucleotide or dimer repeated for the entire 8-mer. This phenomenon is common in genomic sequences and generally is associated with non-functional components, and thus, these were filtered out.

To assess the statistical significance of the computed frequency for the over-represented motifs, we generated random sequences according to the nucleotide composition of the original sets of sequences. The frequencies for the random 8-mers were computed, and the distribution of the frequencies was approximated by the extreme value distribution. We used the MATLAB function gevfit to compute the maximum likelihood estimation of the extreme value distribution. We then overlaid a scaled version of its probability density function, computed using MATLAB function gevpdf, with the histogram of the frequency of the random 8-mer sequences. We repeated this process 100 times for bootstrapping and calculated the p-value. We concluded that the over-representation of the 8-mer motifs in conserved domain is statistically significant if the p-value estimate is less than $1x10^{-4}$ (**Supplementary Figure S13 A**).

Consensus motifs and De novo motif discovery

To identify consensus motifs, 32 enriched 8-mers were phylogenetically clustered into 10 groups. We used the MATLAB function seqlinkage to construct phylogenetic tree from pairwise distances. We then used the MATLAB function seqlogo to identify consensus motifs and their weight matrix for the clustered 8-mer(s) in each group. We downloaded known RNA-binding motifs and TF-binding motifs from JASPAR database (freeze 2014-12-10) [19]. We found known RBPs and TFs per 10 identified consensus that have aligned part of sequence with the consensus by using the MEME suite [24] (Figure 4D).

Enriched motif search in enhancer region of the other end of loop anchor points

We checked whether the 32 enriched motifs found in conserved pcRNA domains are also over-represented in enhancer regions on the other end of the loop anchor points. The definitions for enhancer region and loop anchor points are described in previous Method section, “Identification of HiC loops that overlap pcRNAs”.

We found pcRNA that overlaps with loop anchor points by using Bedtools intersect. The 32 enriched motifs were searched in both pcRNA transcribed sequence as well as enhancer region of the other end of overlapping loop anchor points. We counted a given motif only if the motif was found in both pcRNA and enhancer region. We also searched non-enhancer region of the other end of the loop anchor points as a control set. The counts were normalized by the total length of enhancer or non-enhancer region accordingly (**Supplementary Figure 13B**).

CTCF CLIP-seq data analysis

We downloaded CTCF CLIP-seq data from the Gene Expression Omnibus (GEO, accession number: GSE58242). The mm9 coordinates of the data converted to mm10 coordinates using *liftOver.* The individual data files for two replicates and two strand are combined by time points (day 0 and 3). We used bedtools intersect to identify CTCF-bound tapRNAs and lncRNAs (as control).

Microarray meta-analysis

Probe set sequences of GPL570 platform (Affymetrix Human Genome U133 Plus 2.0 Array) were retrieved from Affymetrix website (http://www.affymetrix.com/), and aligned against hg38 human genome assembly using Blat [25] . We next removed probe sets with less than 90% identity and coverage, and cross-referenced the remaining ones against the pcRNAs genomic coordinates (BED12 format) and the protein coding genes (Gencode version 23) using BEDtools [26]. Probe sets were annotated as pcRNA or as coding gene if at least 70% of its sequence mapped to the reference transcript sequence.

We then download from GEO database (http://www.ncbi.nlm.nih.gov/geo) 63 microarray studies of GPL570 platform, which contained tumor and non-tumour tissue samples (Supplementary Figure 17A, Supplementary Table S6). For each study, raw data (CEL files) were processed and normalized using RMA algorithm, and samples were manually classified as “normal” or “tumor” according to the description provided by authors. We used student t-test (fold-change > 1.25 and p-value < 0.005) to identify transcripts differentially expressed in either tumor or normal samples. Spearman correlation was used to compare the expression between the pcRNA and its associated coding gene. Plots were generated using the following R packages: gplots and ggplot2.

The Cancer Genome Atlas (TCGA) RNA-seq meta-analysis

TCGA RNA-seq V2 Level3 data were downloaded from TCGA Genomic Data Commons Data Portal (https://gdc-portal.nci.nih.gov), consisting of 11,303 samples in 34 cancer projects (33 cancer types). Nine cancer types that do not have corresponding non-tumour samples were filtered out, and the analysis was focused on tumour versus non-tumour comparison. 24 cancer types were used in this meta-analysis: BLCA, BRCA, CESC, CHOL, COAD, ESCA, GBM, HNSC, KICH, KIRC, KIRP, LIHC, LUAD, LUSC, PAAD, PCPG, PRAD, READ, SARC, SKCM, STAD, THCA, THYM, UCEC (https://gdc-portal.nci.nih.gov). The nine filtered cancer types were ACC, DLBC, LAML, LGG, MESO, OV, TGCT, UCS and UVM.

To extract expression values from TCGA RNA-seq data, we used genomic coordinates to retrieve UCSC Transcript IDs that correspond to the identifiers in TCGA RNA-seq V2 Level3 data (isoform level). The GAF (General Annotation Format) file was used to map the coordinate to UCSC Transcript ID, and it was downloaded form https://tcga-data.nci.nih.gov/docs/GAF/GAF.hg19.June2011.bundle/outputs/TCGA.hg19.June2011.gaf. This file contains genomic annotations shared by all TCGA projects. More details of the GAF file format can be found at https://tcga-data.nci.nih.gov/docs/GAF/GAF3.0/GAF_v3_file_description.docx. We filtered out any coding exons overlapping UCSC Transcript IDs to eliminate expression value of coding genes and evaluate lncRNA expression.

We could find the expression values of 443 pcRNAs and 203 tapRNAs in TCGA data, as many of non-coding regions are not yet fully annotated in the TCGA RNA-seq V2 Level3 data. The expression value of pcRNAs and tapRNAs were extracted and clustered by un-supervised Pearson correlation method (Supplementary Figure 18A). The expression values of tapRNA-associated coding genes were also extracted and used to generate the heat-map (Supplementary Figure 18B), which shows the similar pattern of expression with tapRNAs across the cancer types.

To show that tapRNAs and associated coding genes have similar expression profiles in cancers we generated a Spearman's Rank-Order Correlation heatmap (Figure 6A) between tapRNAs and their associated coding genes based on the TCGA RNA-seq data. We used the MatLab function *corr* to calculate the Spearman's rho. This function takes two matrices X (197-by-8,850 expression profiling matrix of tapRNA) and Y (197-by-8,850 expression profiling matrix of tapRNA-assocated coding gene) and returns an 8,850-by-8,850 matrix containing the pairwise correlation coefficient between each pair of 8,850 columns (TCGA cancer samples in Supplementary Figure 18A and B). Thus, the rank-order correlation matrix that we computed from the matrices of expression profiling data (Supplementary Figure S18A and B) allowed us to compare the correlation between two column vectors i.e. cancer samples. This function also returns a matrix of p-values for testing the hypothesis of no correlation against the alternative that there is a nonzero correlation. Each element of a matrix of p-values is the p value for the corresponding element of Spearman's rho. The p-values for Spearman's rho are calculated using large-sample approximations. To check significance level of correlation between tapRNA and its associated coding gene, the diagonal of the p-value matrix was extracted and used. The median is 1.31×10^{-11} and the mean is 1.03×10^{-4} with standard deviation 0.0029.

To identify cancer-specific tapRNAs, we considered not only the global expression pattern of a given tapRNA in each cancer type, but also expression pattern of specific sub-group that is significantly distinct, to take into account cancer sample heterogeneity. Thus, two conditions were applied: (1) average expression level of a tapRNA in a given cancer type is in top 10% or bottom 10% and (2) a tapRNA has at least 10% of samples in a given cancer type that are significantly up-regulated (Z-score > 2) or down-regulated (Z-score < -2).

International Cancer Genome Consortium (ICGC) pan-cancer somatic mutation analysis

ICGC Data Portal Release 21 was downloaded from ICGC Data Portal (https://dcc.icgc.org/releases/release_21), which has been released on May 16, 2016. This release included 68 Cancer Projects, 21 Cancer primary sites, 15,613 donors with molecular data in DCC, 18,677 total donors, 42,584,179 simple somatic mutations, and 57,656 mutated genes.

We tested whether CTCF and ZNF263 motifs that are localised inside tapRNA loci or promoters have an increased chance to have cancer somatic mutations. To do this, we first used the JASPAR database to get the 19 nucleotide-long CTCF consensus motif and 21 nucleotide-long ZNF263 motif. The two motifs were then used to scan the whole genome to find all possible matching regions by using PWMscan (Ambrosini G., PWMTools, http://ccg.vital-it.ch/pwmtools). The matched regions to CTCF or ZNF263 consensus motifs were then filtered with two conditions to generate two different categories corresponding to: (A) DNA region bound by its protein using ENCODE ChIP-seq data (e.g. CTCF matched motif regions bound by CTCF protein) or (B) the region has evolutionary sequence conservation using PhyloP score. Finally, we separately searched the matched motif regions of the two categories to assess whether the motif regions contain cancer somatic mutation sites. The counted mutated motif numbers were then normalised by the total number of CTCF/ZNF263 motifs that were found within tapRNA loci or promoters. As a control, we also searched the two categories using Gencode lncRNAs that are spliced. The counted mutated motif numbers in lncRNA were also normalised by the total number of CTCF/ZNF263 motifs within lncRNA loci or promoters. To directly compare the enrichment of mutated motifs between CTCF and ZNF263, the normalised counts were then normalised by the total number of CTCF/ZNF263 motifs that were found in entire genome because there are more CTCF motifs in the genome than ZNF263 motifs.

Supplementary References

1. Needleman SB, Wunsch CD: **A general method applicable to the search for similarities in the amino acid sequence of two proteins.** *J Mol Biol* 1970, **48:**443-453.
2. Mas-Ponte D, Carlevaro-Fita J, Palumbo E, Hermoso Pulido T, Guigo R, Johnson R: **LncATLAS database for subcellular localization of long noncoding RNAs.** *RNA* 2017, **23:**1080-1087.
3. Bernstein BE, Mikkelsen TS, Xie X, Kamal M, Huebert DJ, Cuff J, Fry B, Meissner A, Wernig M, Plath K, et al: **A bivalent chromatin structure marks key developmental genes in embryonic stem cells.** *Cell* 2006, **125:**315-326.
4. Rao SS, Huntley MH, Durand NC, Stamenova EK, Bochkov ID, Robinson JT, Sanborn AL, Machol I, Omer AD, Lander ES, Aiden EL: **A 3D map of the human genome at kilobase resolution reveals principles of chromatin looping.** *Cell* 2014, **159:**1665-1680.
5. Kung JT, Kesner B, An JY, Ahn JY, Cifuentes-Rojas C, Colognori D, Jeon Y, Szanto A, del Rosario BC, Pinter SF, et al: **Locus-specific targeting to the X chromosome revealed by the RNA interactome of CTCF.** *Mol Cell* 2015, **57:**361-375.
6. Down TA, Piipari M, Hubbard TJ: **Dalliance: interactive genome viewing on the web.** *Bioinformatics* 2011, **27:**889-890.
7. Rosenbloom KR, Armstrong J, Barber GP, Casper J, Clawson H, Diekhans M, Dreszer TR, Fujita PA, Guruvadoo L, Haeussler M, et al: **The UCSC Genome Browser database: 2015 update.** *Nucleic Acids Res* 2015, **43:**D670-681.
8. Langmead B, Salzberg SL: **Fast gapped-read alignment with Bowtie 2.** *Nat Methods* 2012, **9:**357-359.
9. Harrow J, Frankish A, Gonzalez JM, Tapanari E, Diekhans M, Kokocinski F, Aken BL, Barrell D, Zadissa A, Searle S, et al: **GENCODE: the reference human genome annotation for The ENCODE Project.** *Genome Res* 2012, **22:**1760-1774.
10. Karolchik D, Hinrichs AS, Furey TS, Roskin KM, Sugnet CW, Haussler D, Kent WJ: **The UCSC Table Browser data retrieval tool.** *Nucleic Acids Res* 2004, **32:**D493-496.
11. Kim D, Pertea G, Trapnell C, Pimentel H, Kelley R, Salzberg SL: **TopHat2: accurate alignment of transcriptomes in the presence of insertions, deletions and gene fusions.** *Genome Biol* 2013, **14:**R36.
12. Kent WJ, Sugnet CW, Furey TS, Roskin KM, Pringle TH, Zahler AM, Haussler D: **The human genome browser at UCSC.** *Genome Res* 2002, **12:**996-1006.
13. Gascoigne DK, Cheetham SW, Cattenoz PB, Clark MB, Amaral PP, Taft RJ, Wilhelm D, Dinger ME, Mattick JS: **Pinstripe: a suite of programs for integrating transcriptomic and proteomic datasets identifies novel proteins and improves differentiation of protein-coding and non-coding genes.** *Bioinformatics* 2012, **28:**3042-3050.
14. Wang L, Park HJ, Dasari S, Wang S, Kocher JP, Li W: **CPAT: Coding-Potential Assessment Tool using an alignment-free logistic regression model.** *Nucleic Acids Res* 2013, **41:**e74.
15. Cabili MN, Trapnell C, Goff L, Koziol M, Tazon-Vega B, Regev A, Rinn JL: **Integrative annotation of human large intergenic noncoding RNAs reveals global properties and specific subclasses.** *Genes Dev* 2011, **25:**1915-1927.
16. Liu SJ, Horlbeck MA, Cho SW, Birk HS, Malatesta M, He D, Attenello FJ, Villalta JE, Cho MY, Chen Y, et al: **CRISPRi-based genome-scale identification of functional long noncoding RNA loci in human cells.** *Science* 2017, **355**.
17. van Dongen S, Abreu-Goodger C, Enright AJ: **Detecting microRNA binding and siRNA off-target effects from expression data.** *Nat Methods* 2008, **5:**1023-1025.
18. Dunning MJ, Smith ML, Ritchie ME, Tavare S: **beadarray: R classes and methods for Illumina bead-based data.** *Bioinformatics* 2007, **23:**2183-2184.
19. Mathelier A, Fornes O, Arenillas DJ, Chen CY, Denay G, Lee J, Shi W, Shyr C, Tan G, Worsley-Hunt R, et al: **JASPAR 2016: a major expansion and update of the open-access database of transcription factor binding profiles.** *Nucleic Acids Res* 2016, **44:**D110-115.
20. Kheradpour P, Kellis M: **Systematic discovery and characterization of regulatory motifs in ENCODE TF binding experiments.** *Nucleic Acids Res* 2014, **42:**2976-2987.
21. Jolma A, Yan J, Whitington T, Toivonen J, Nitta KR, Rastas P, Morgunova E, Enge M, Taipale M, Wei G, et al: **DNA-binding specificities of human transcription factors.** *Cell* 2013, **152:**327-339.

22. Ernst J, Kheradpour P, Mikkelsen TS, Shoresh N, Ward LD, Epstein CB, Zhang X, Wang L, Issner R, Coyne M, et al: **Mapping and analysis of chromatin state dynamics in nine human cell types.** *Nature* 2011, **473:**43-49.
23. Huang da W, Sherman BT, Lempicki RA: **Systematic and integrative analysis of large gene lists using DAVID bioinformatics resources.** *Nat Protoc* 2009, **4:**44-57.
24. Bailey TL, Boden M, Buske FA, Frith M, Grant CE, Clementi L, Ren J, Li WW, Noble WS: **MEME SUITE: tools for motif discovery and searching.** *Nucleic Acids Res* 2009, **37:**W202-208.
25. Kent WJ: **BLAT--the BLAST-like alignment tool.** *Genome Res* 2002, **12:**656-664.
26. Quinlan AR: **BEDTools: The Swiss-Army Tool for Genome Feature Analysis.** *Curr Protoc Bioinformatics* 2014, **47:**11 12 11-11 12 34.
