# Supplementary material for: Genomic positional conservation identifies topological anchor point RNAs linked to developmental loci
Source: Genome Biol. 2018 Mar 15;19:32. doi: 10.1186/s13059-018-1405-5 (PMC5853149; doi:10.1186/s13059-018-1405-5)
Supplement: Supplementary file 4 — Supplementary figures. (PDF 14168 kb) [file 13059_2018_1405_MOESM4_ESM.pdf]

Supplementary Figure 1

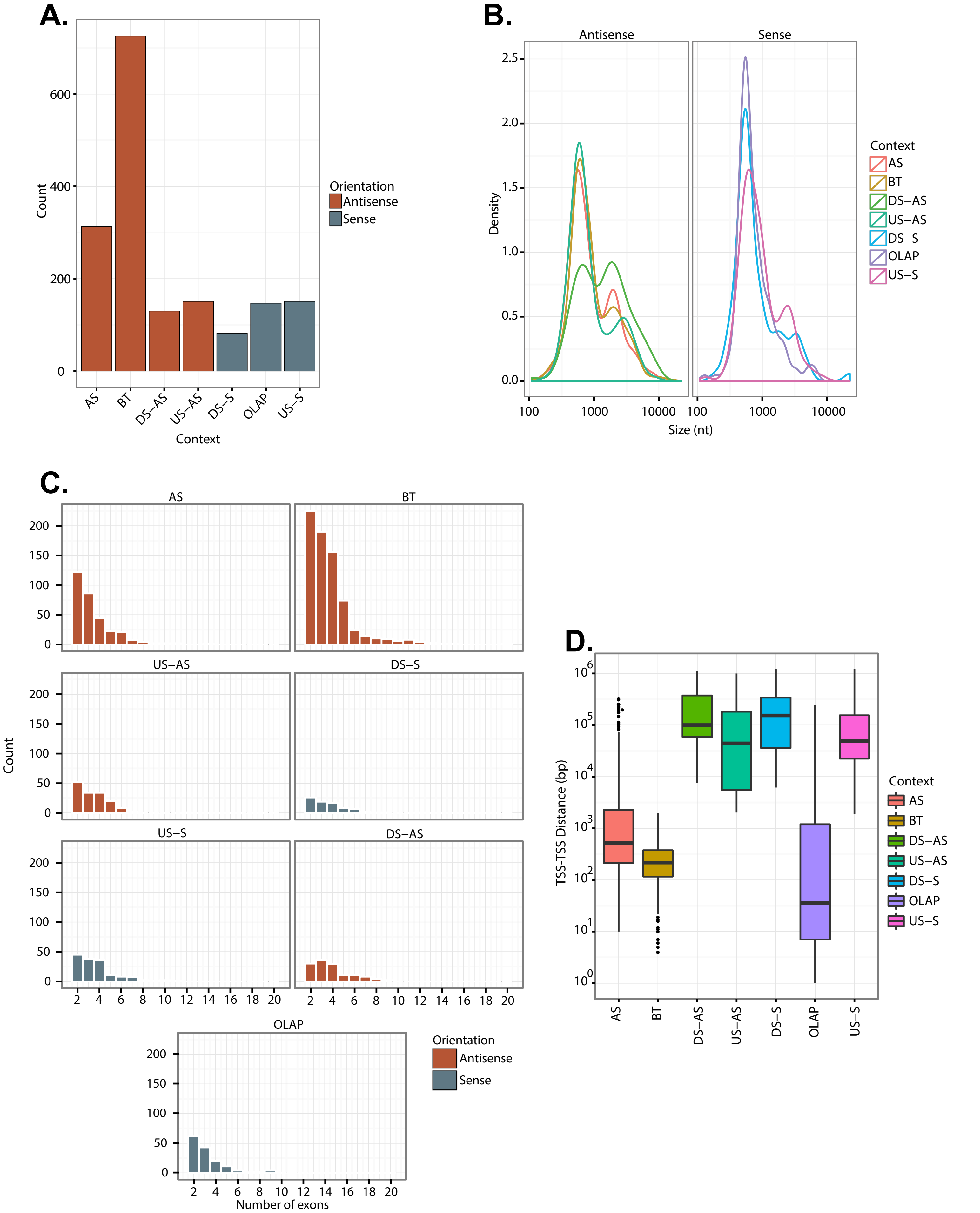

Supplementary Figure 2

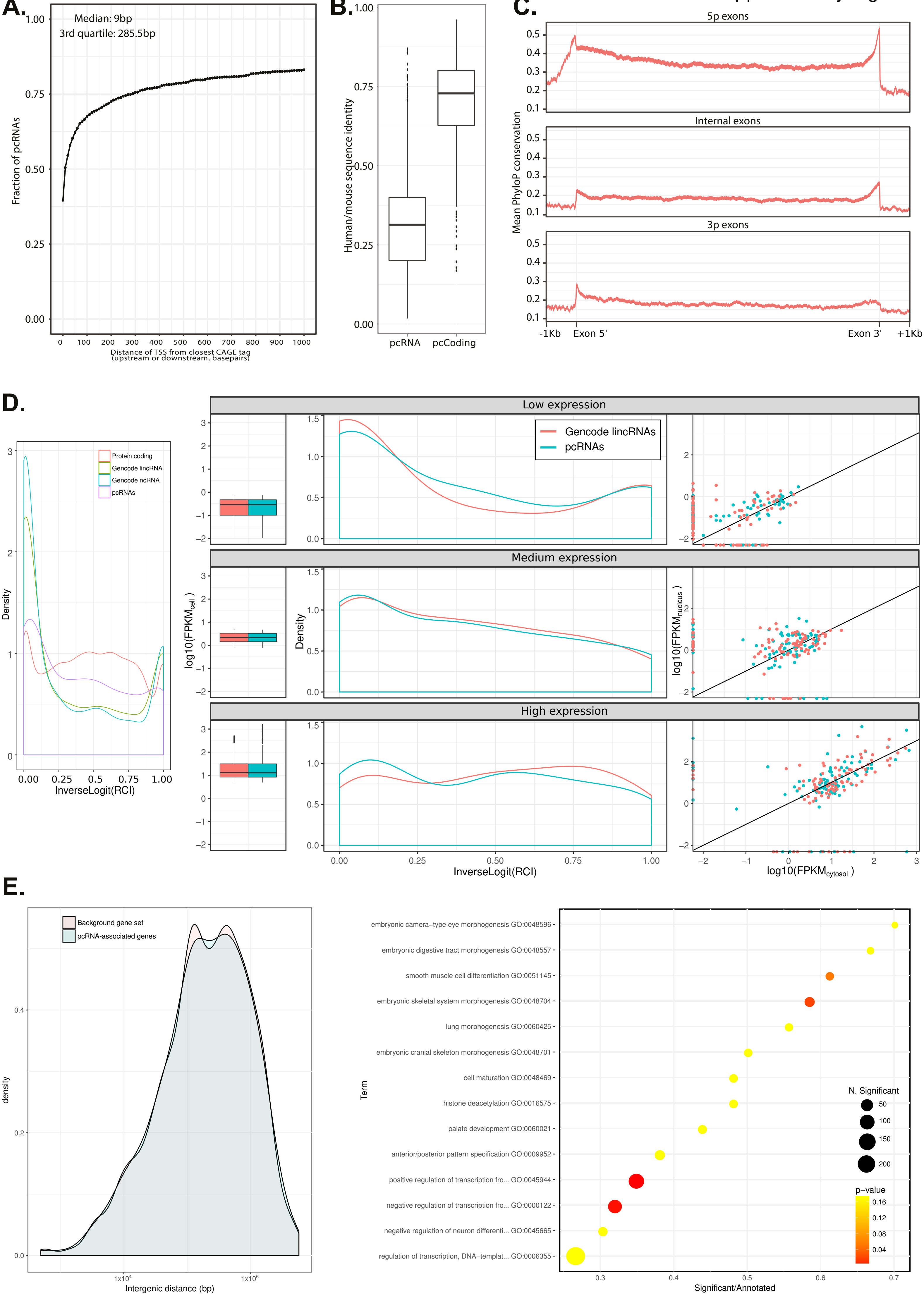

Supplementary Figure 3

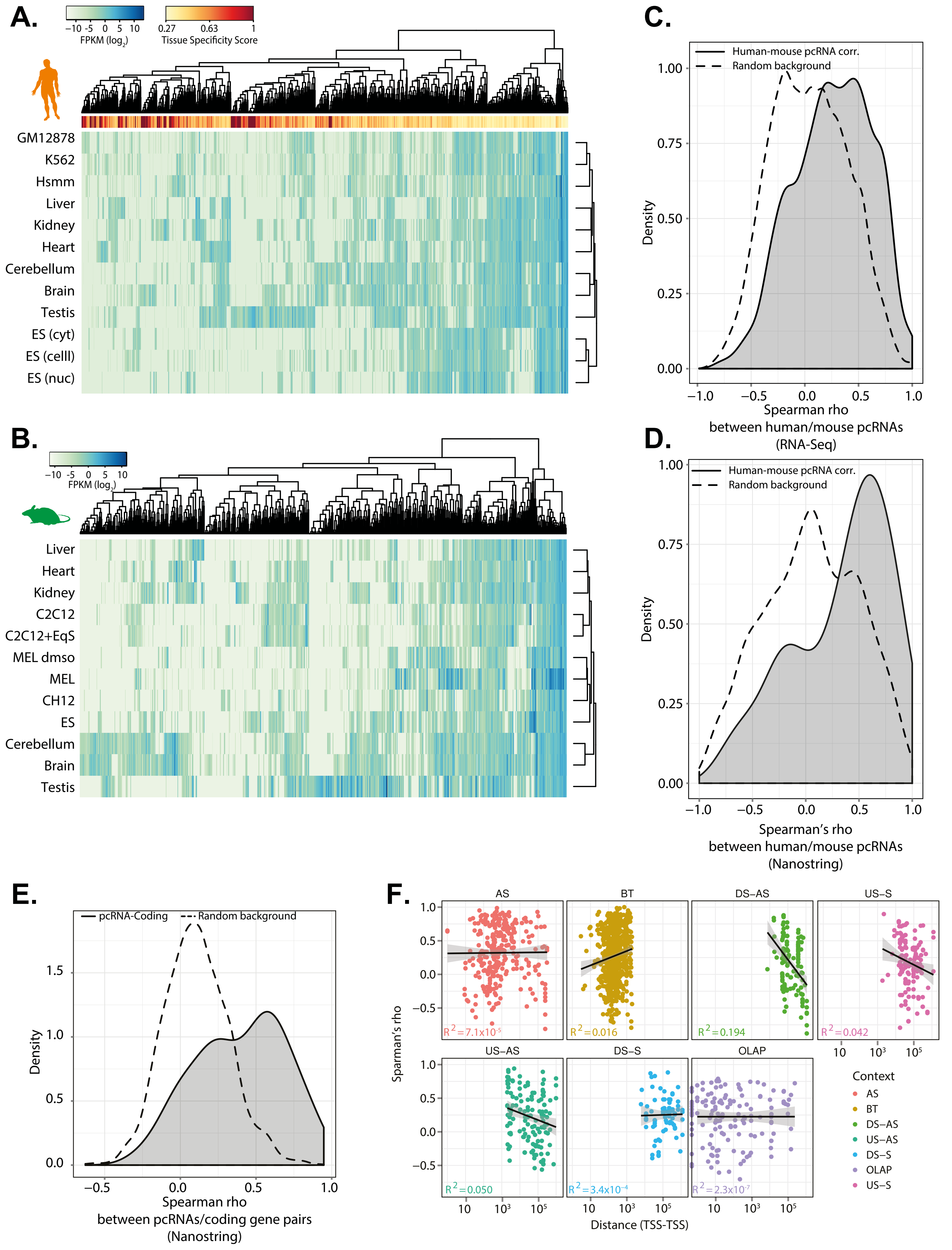

Supplementary Figure 4

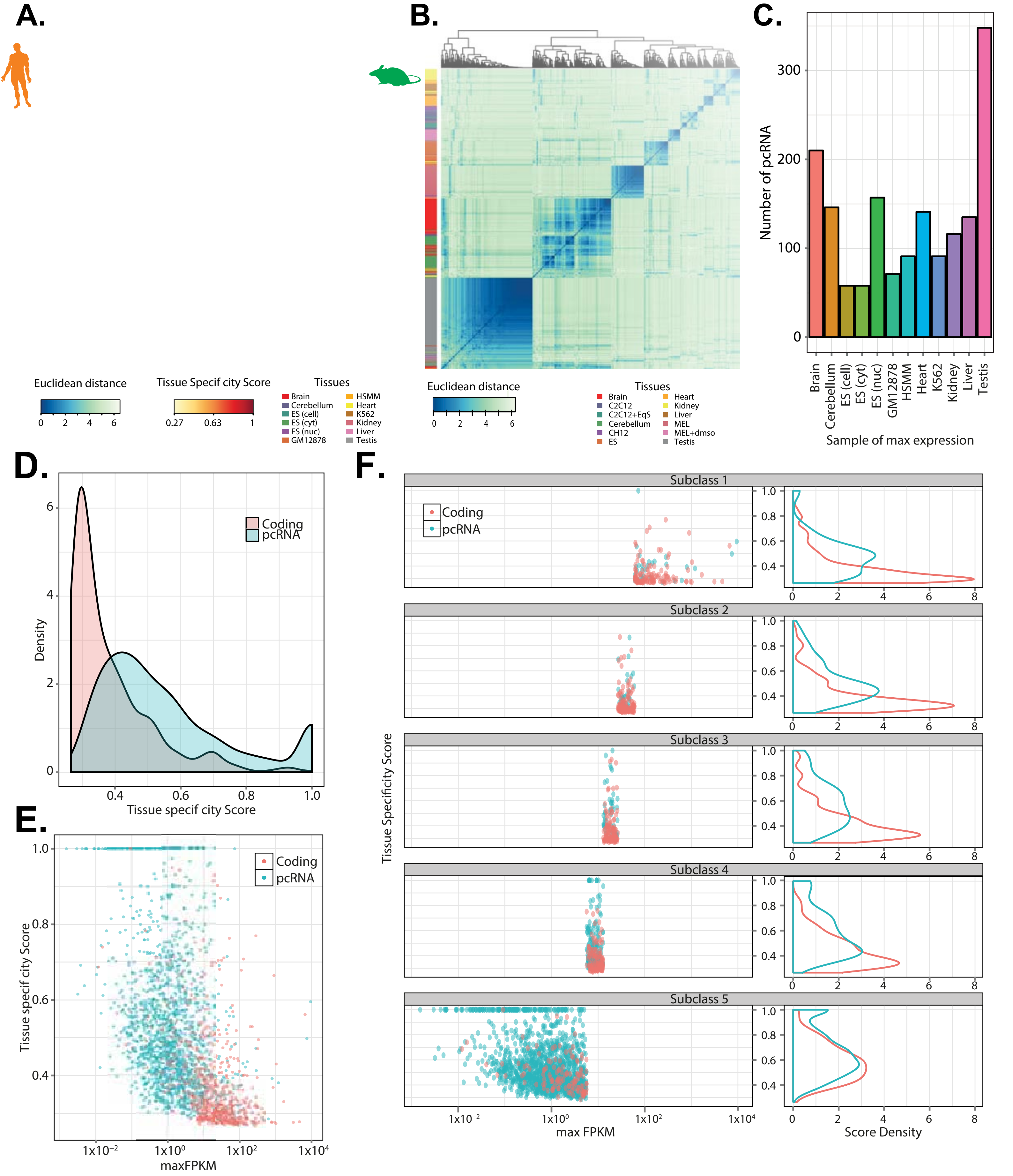

Supplementary Figure 5

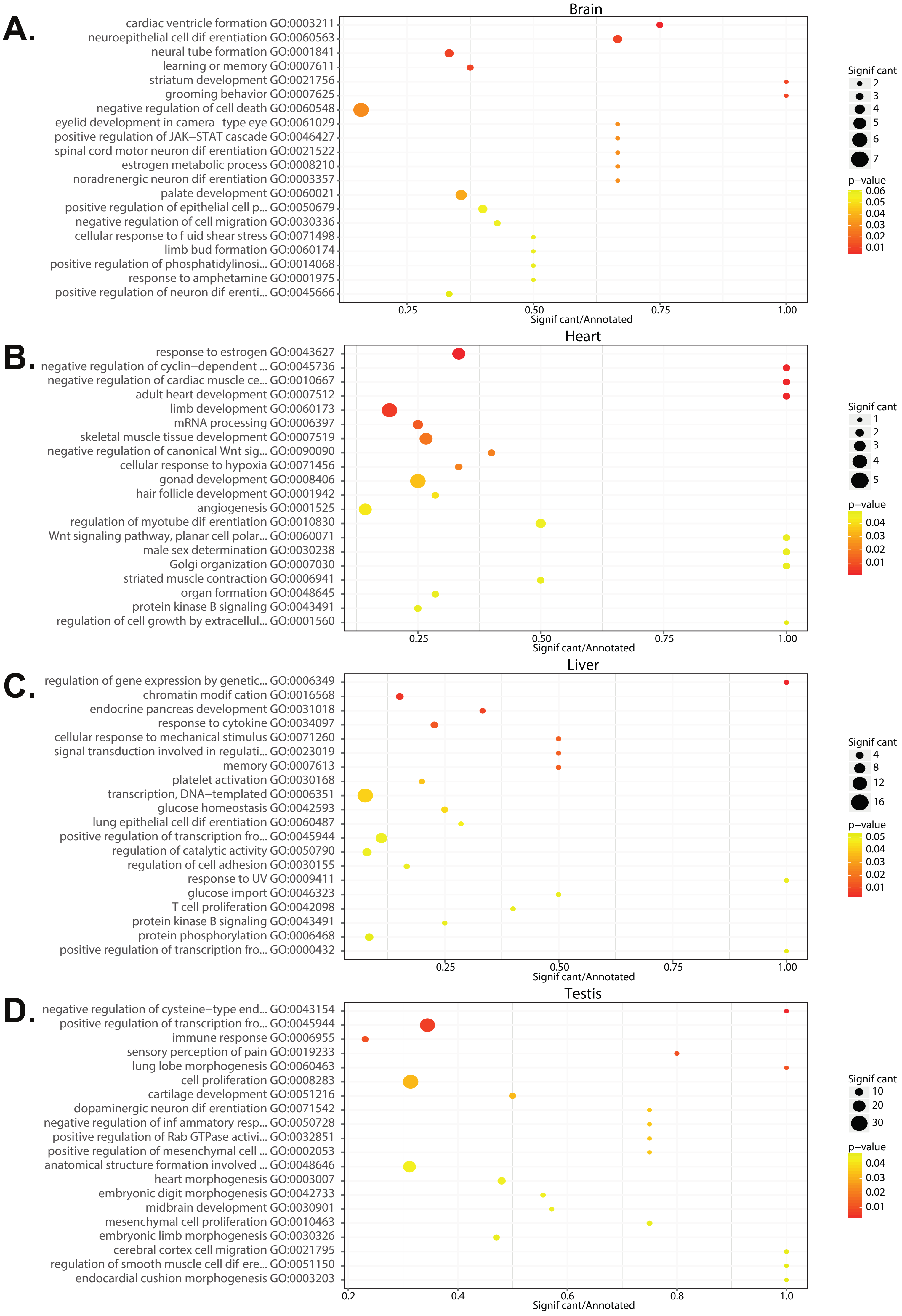

Supplementary Figure 6

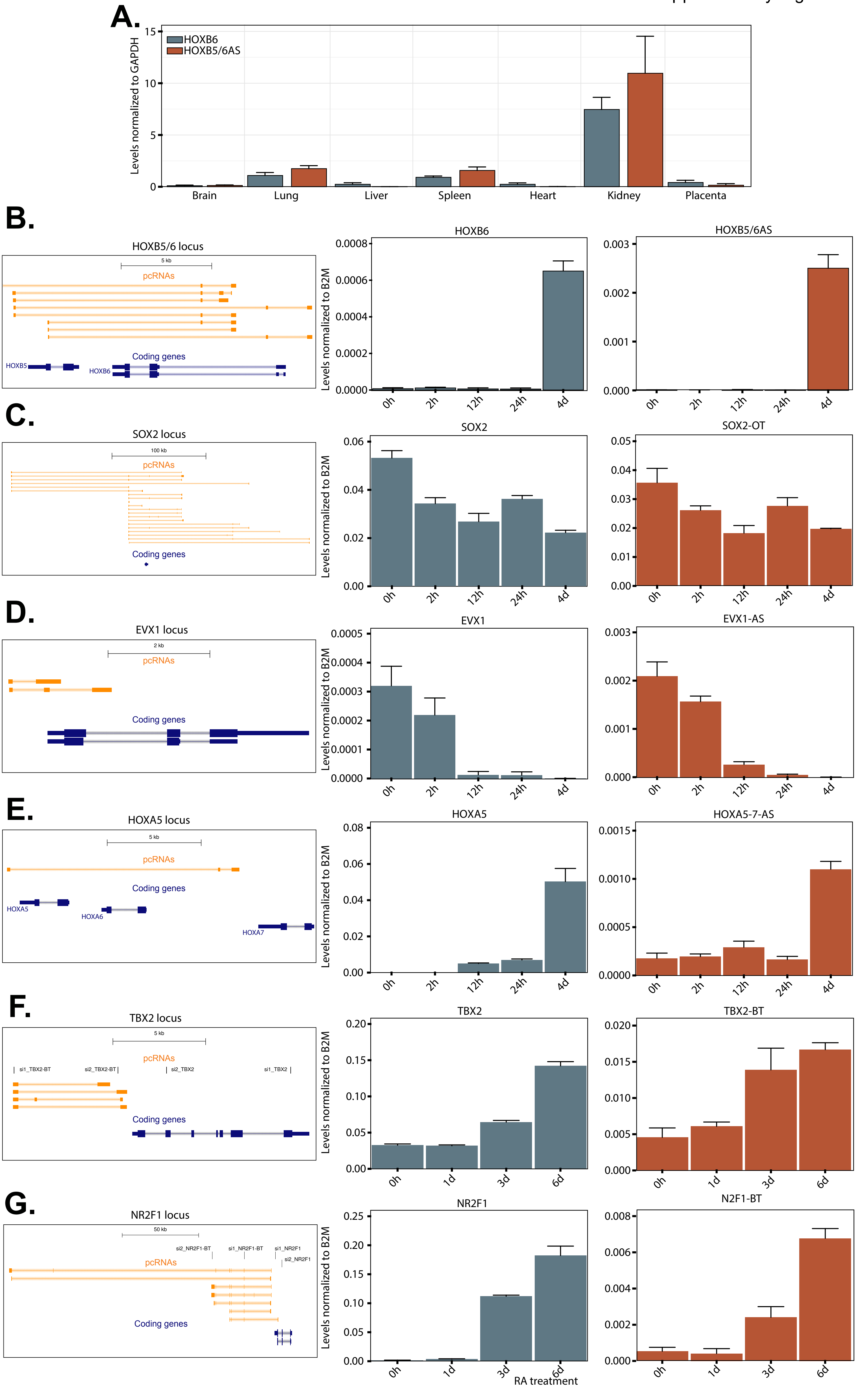

Supplementary Figure 7

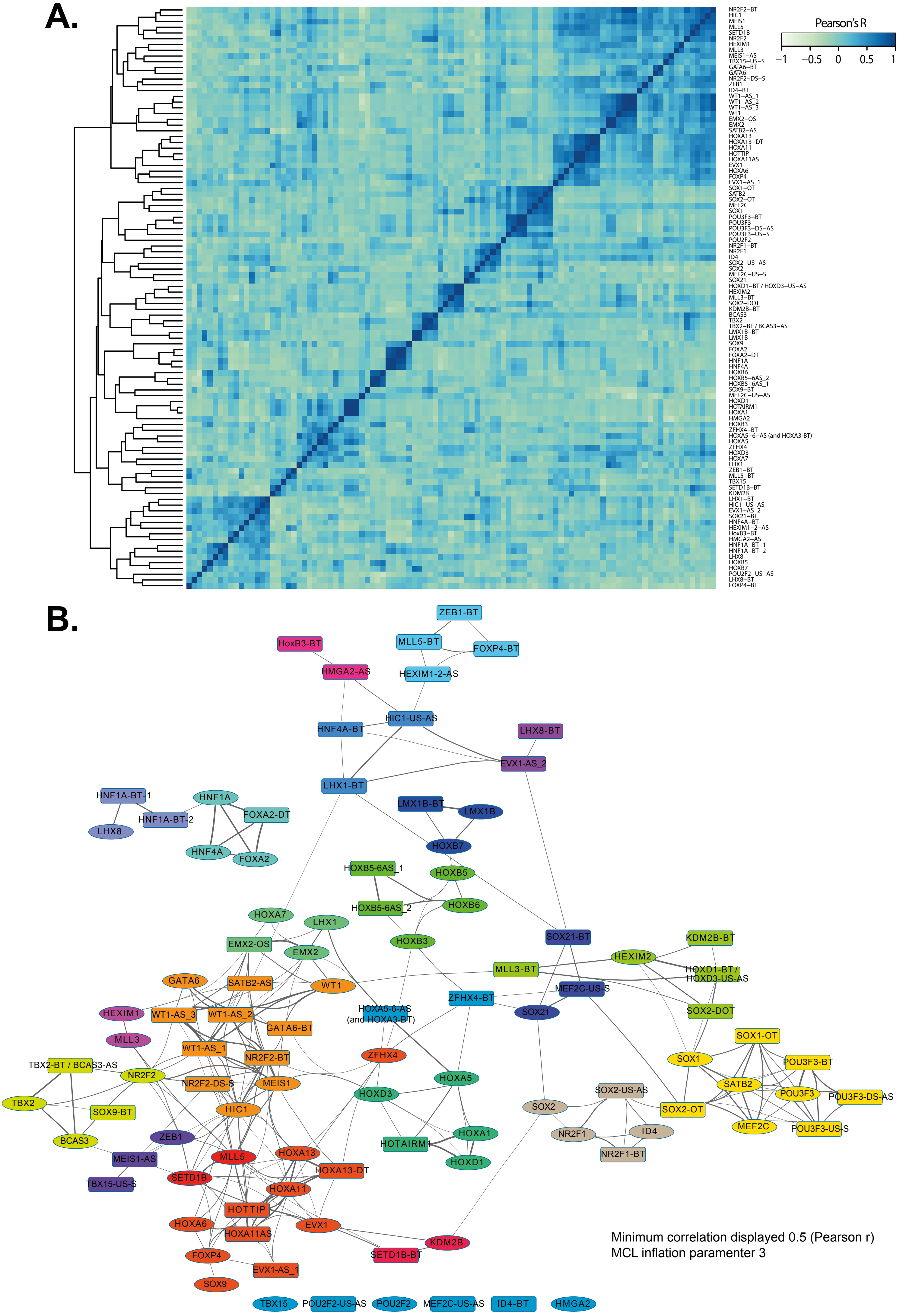

Supplementary Figure 8

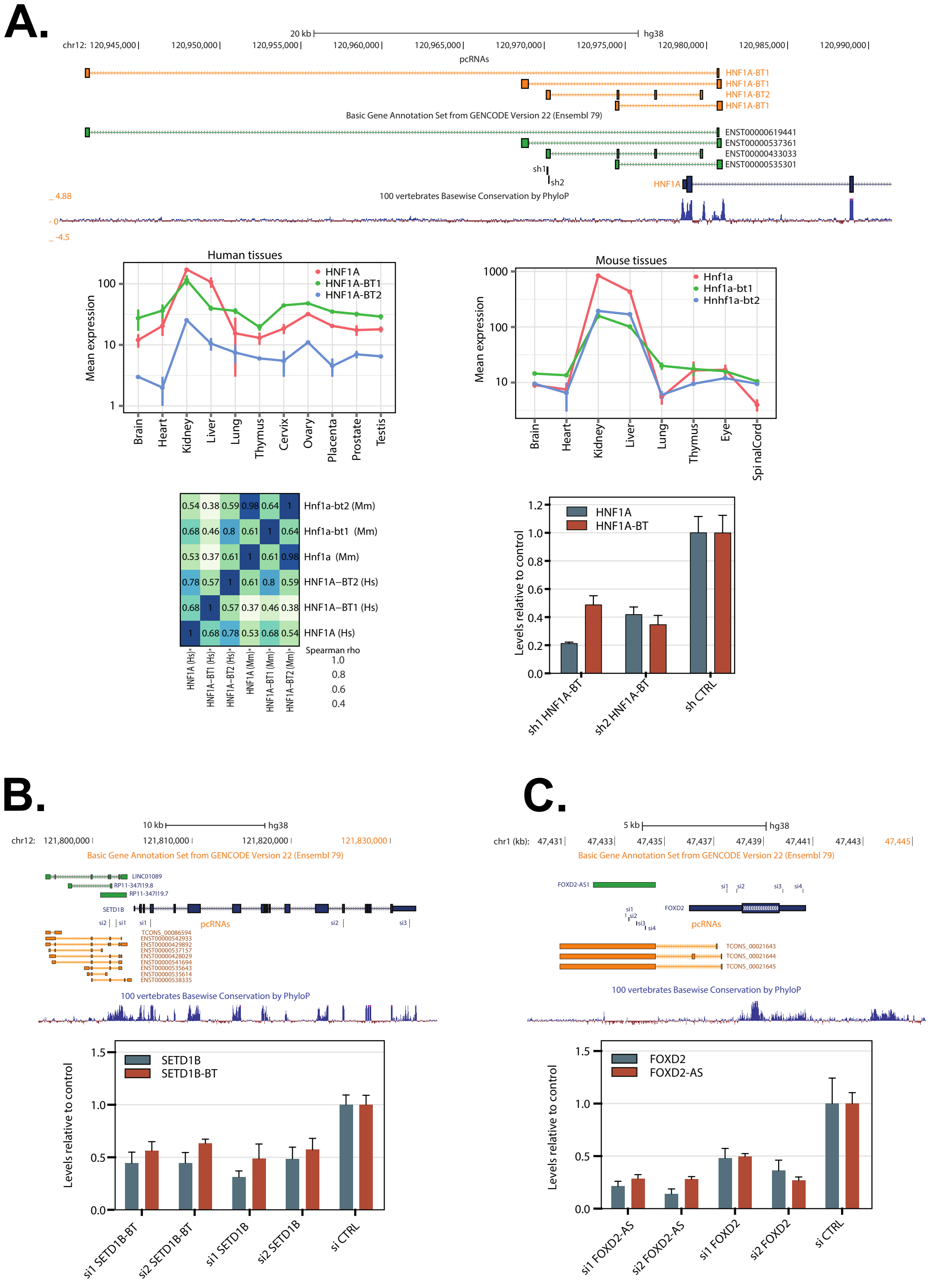

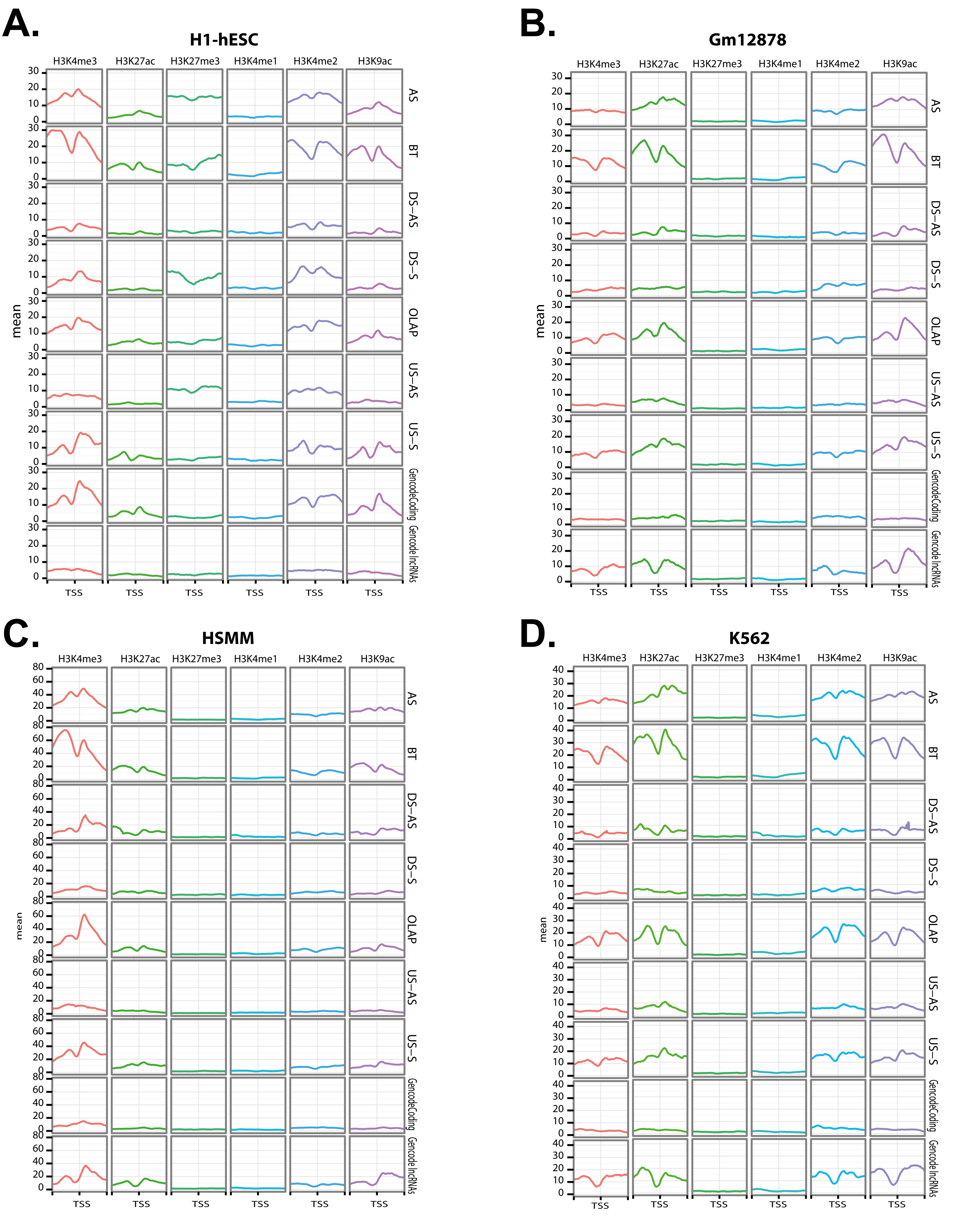
A.
H1-hESC
B.
Gm12878
C.
HSMM
D.
K562
H3K4me3
H3K27ac
H3K27me3
H3K4me1
H3K4me2
H3K9ac
AS
BT
DS−AS
DS−S
OLAP
US−AS
US−S
GencodeCoding
Gencode lncRNAs
mean
TSS

Supplementary Figure 10

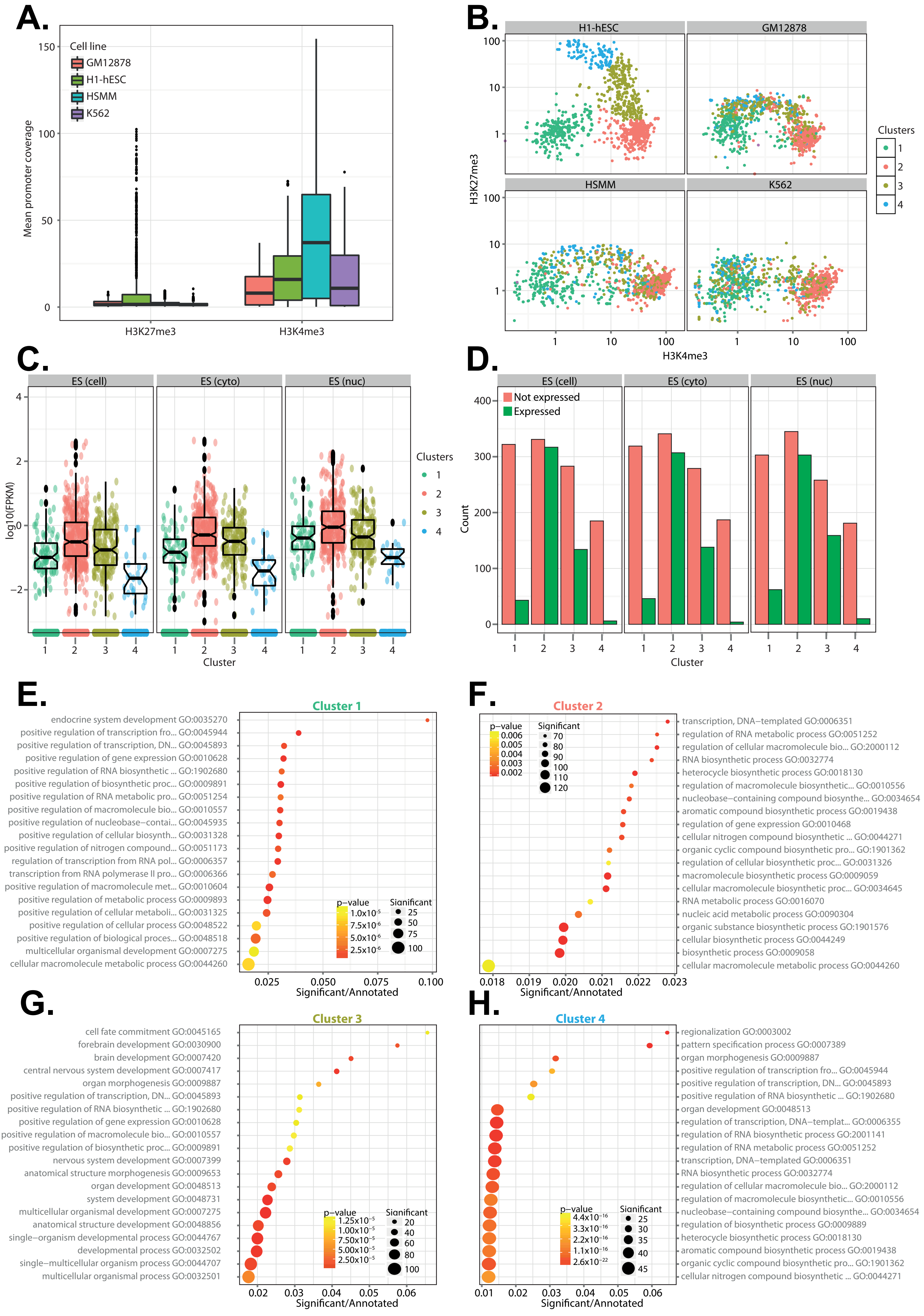

Supplementary Figure 11

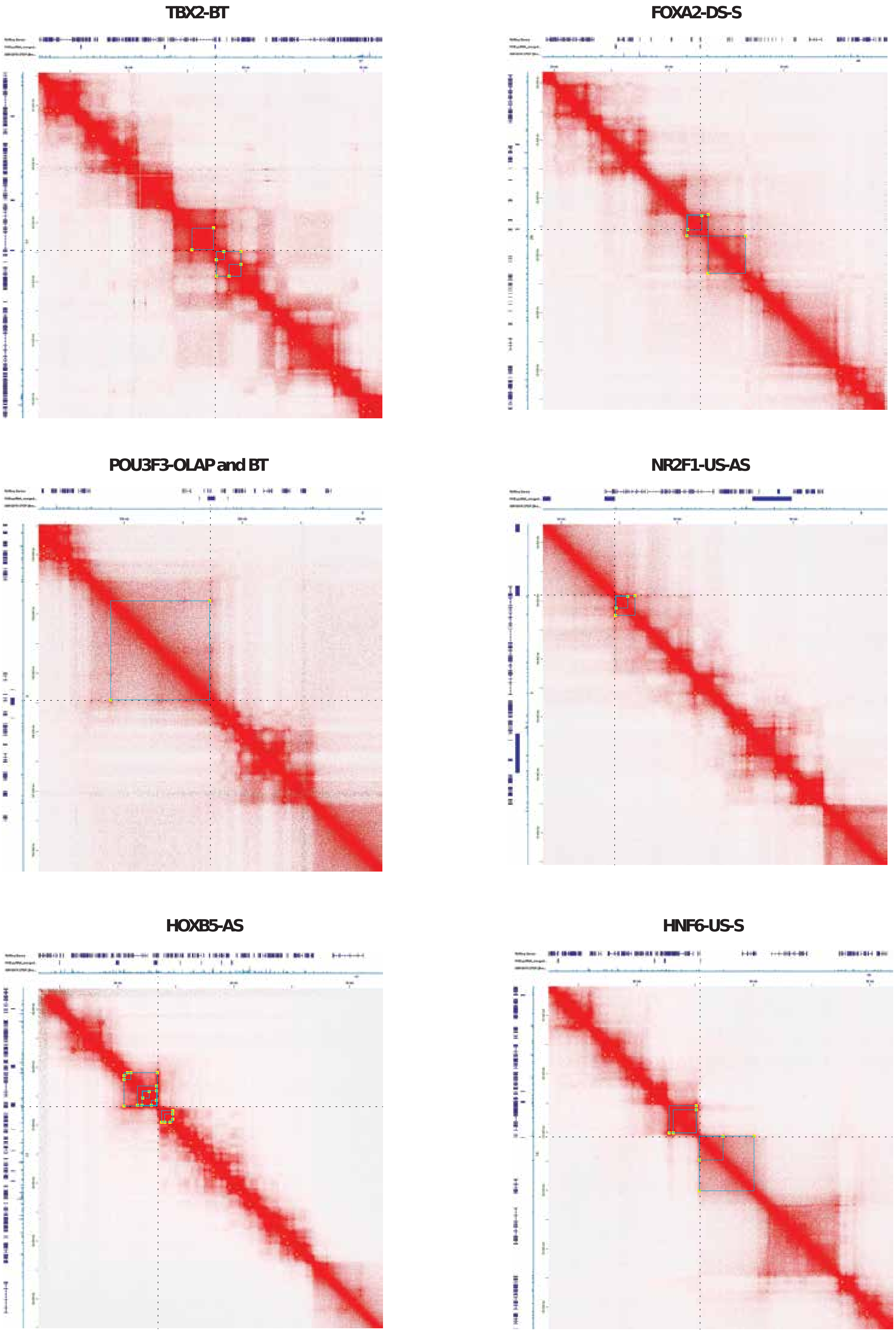

Supplementary Figure 12

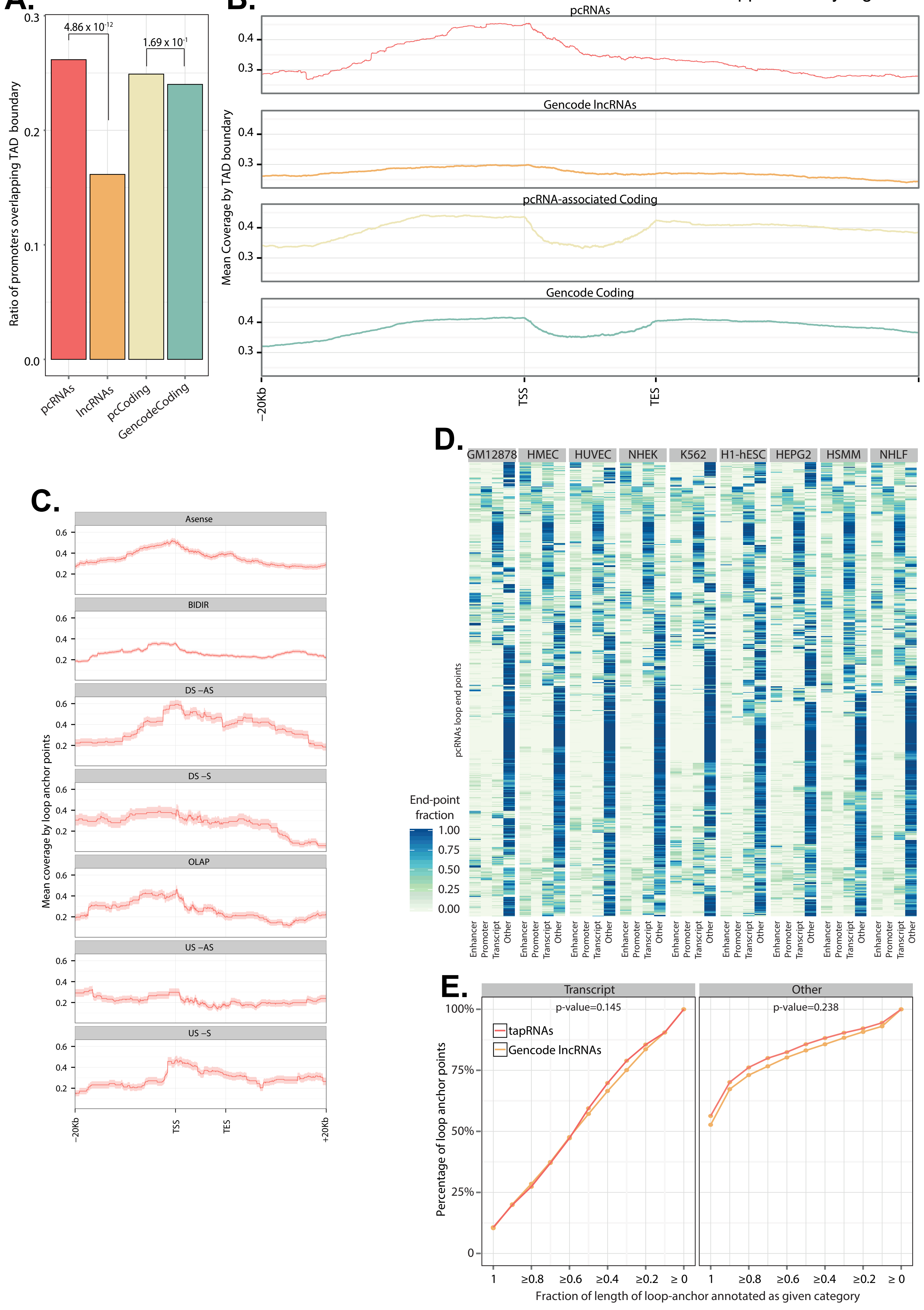

Supplementary Figure 13

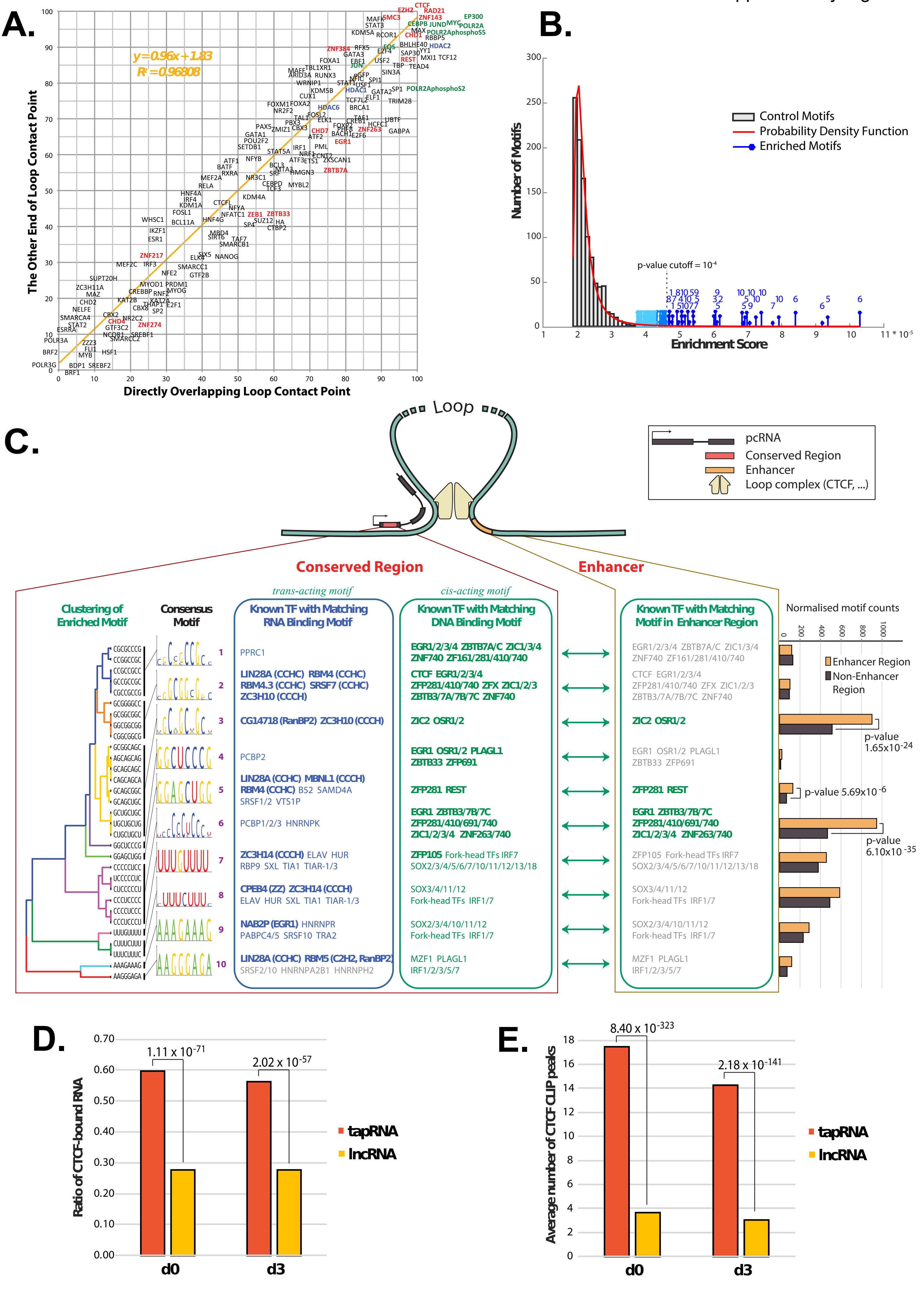

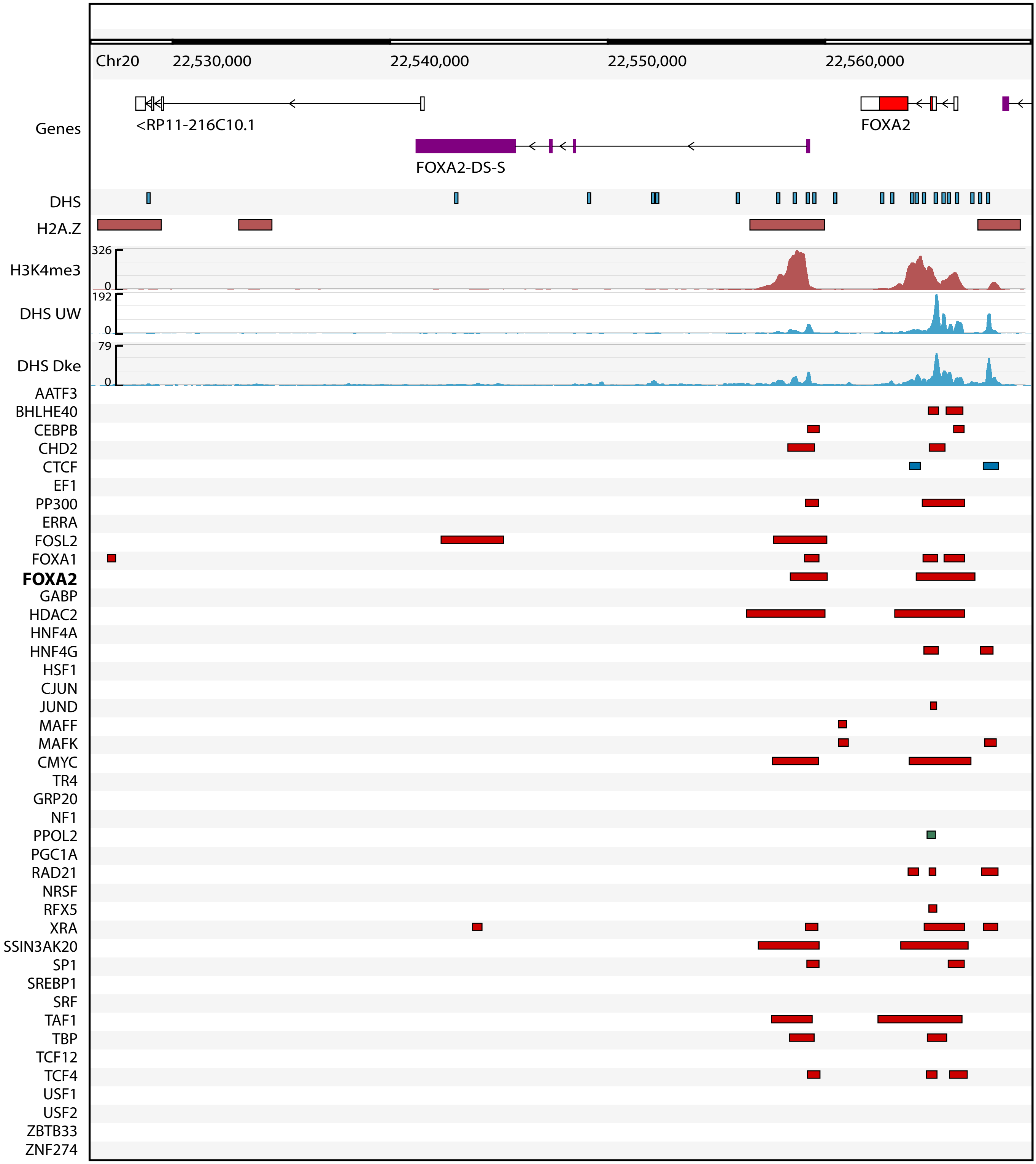
Chr20
22,530,000
22,540,000
22,550,000
22,560,000
Genes
<RP11-216C10.1
FOXA2
FOXA2-DS-S
DHS
H2A.Z
H3K4me3
326
0
DHS UW
192
0
DHS Dke
79
0
AATF3
BHLHE40
CEBPB
CHD2
CTCF
EF1
PP300
ERRA
FOSL2
FOXA1
FOXA2
GABP
HDAC2
HNF4A
HNF4G
HSF1
CJUN
JUND
MAFF
MAFK
CMYC
TR4
GRP20
NF1
PPOL2
PGC1A
RAD21
NRSF
RFX5
XRA
SSIN3AK20
SP1
SREBP1
SRF
TAF1
TBP
TCF12
TCF4
USF1
USF2
ZBTB33
ZNF274

Supplementary Figure 15

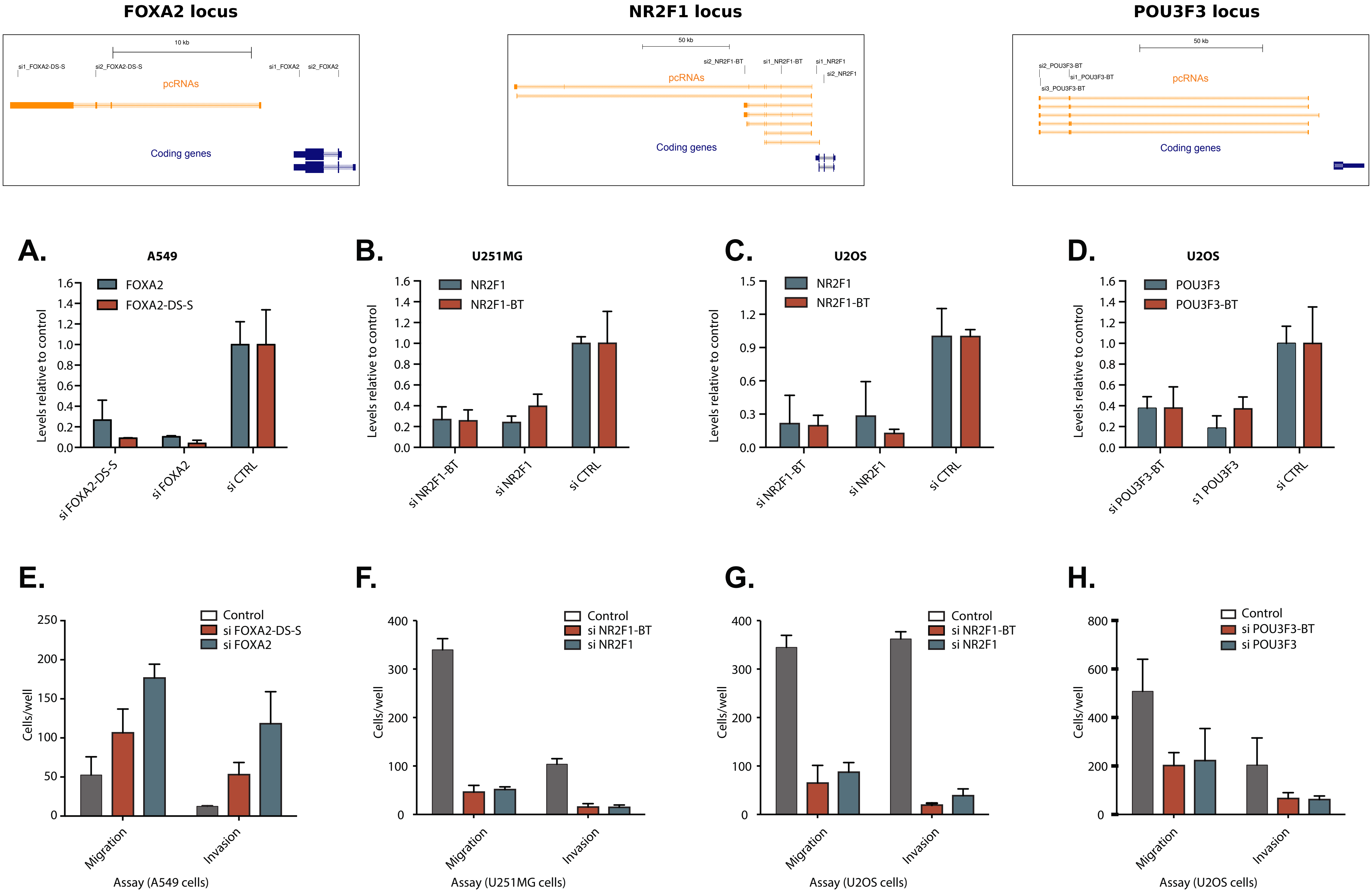

Supplementary Figure 16

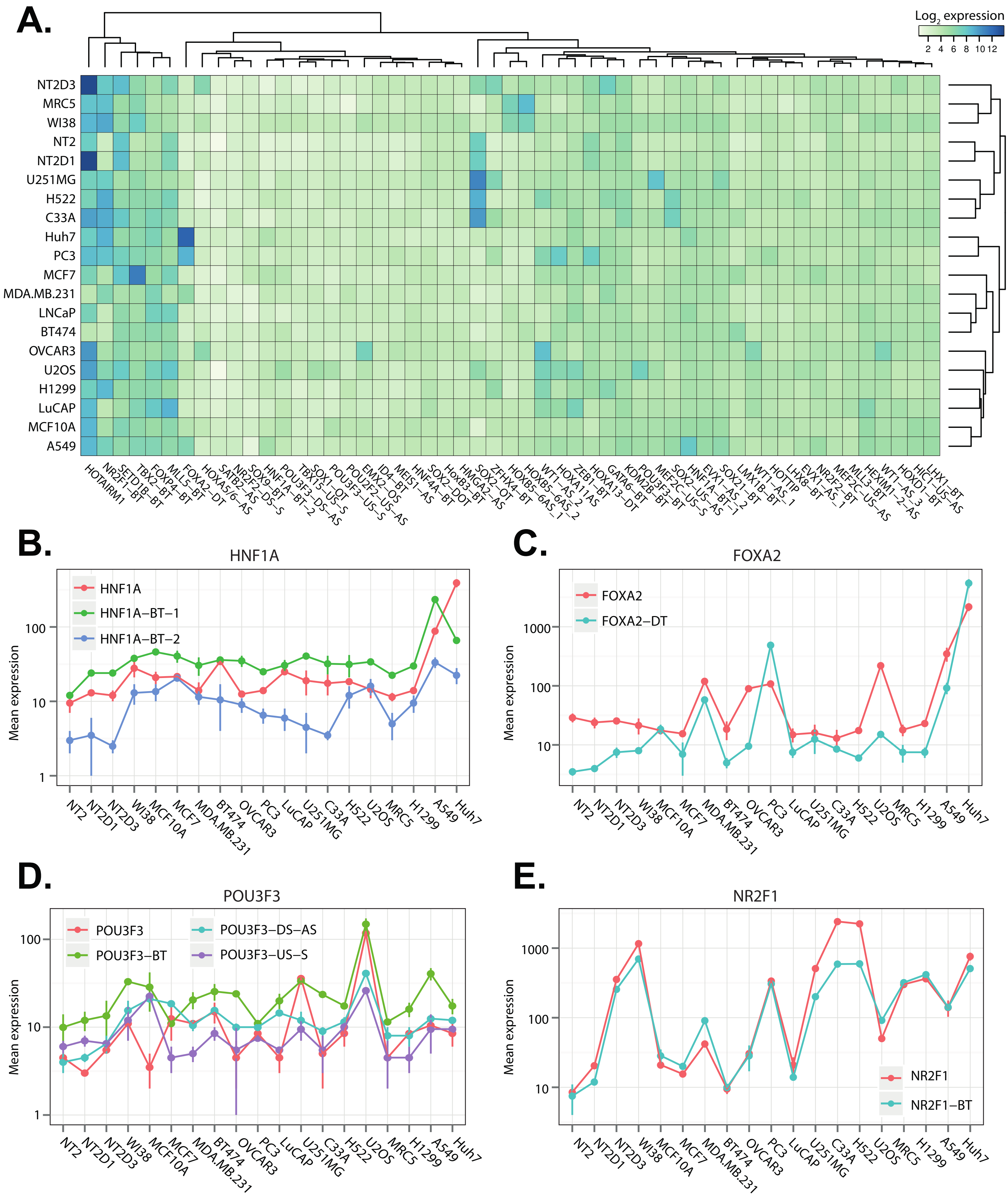

Supplementary Figure 17

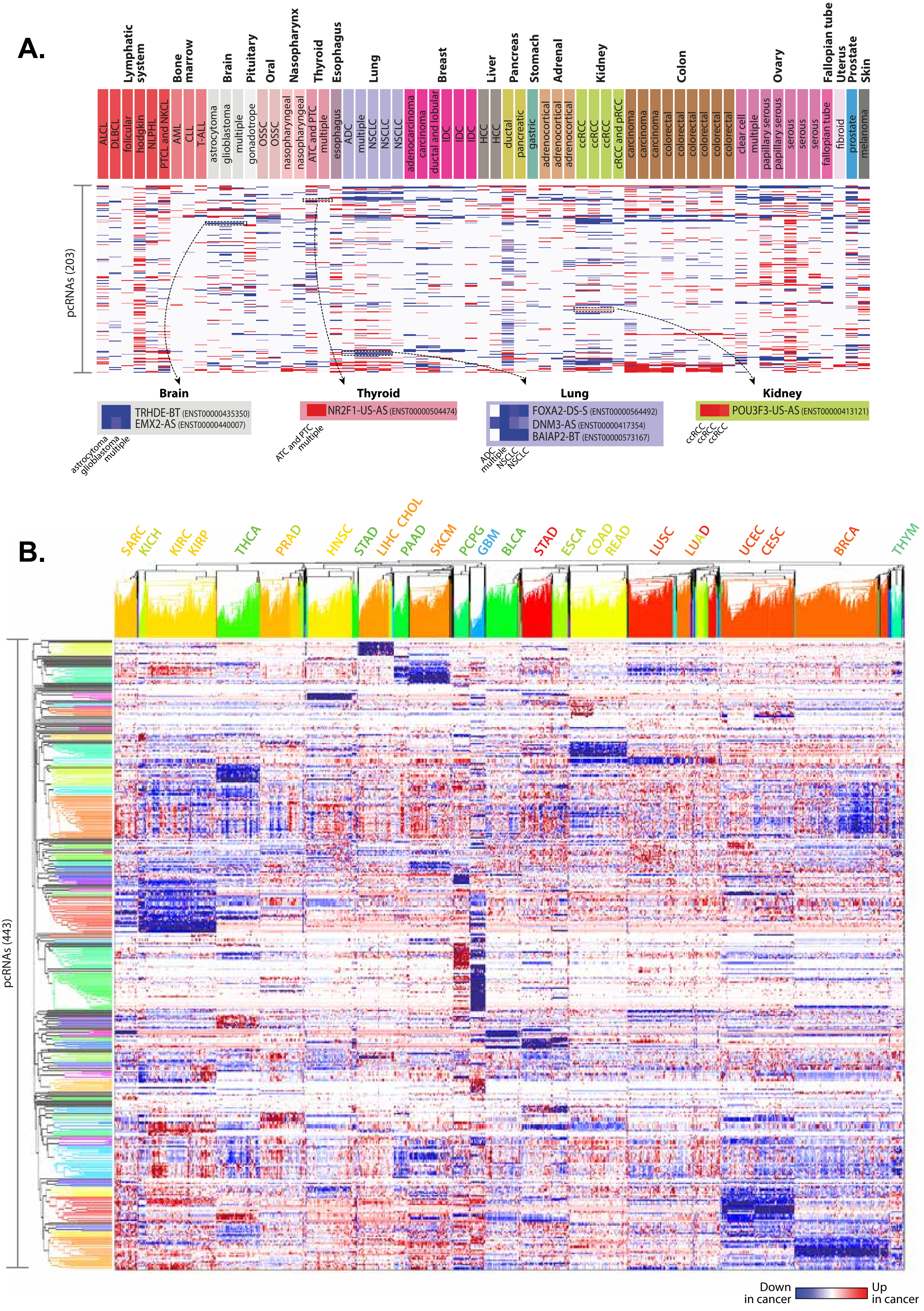

A.

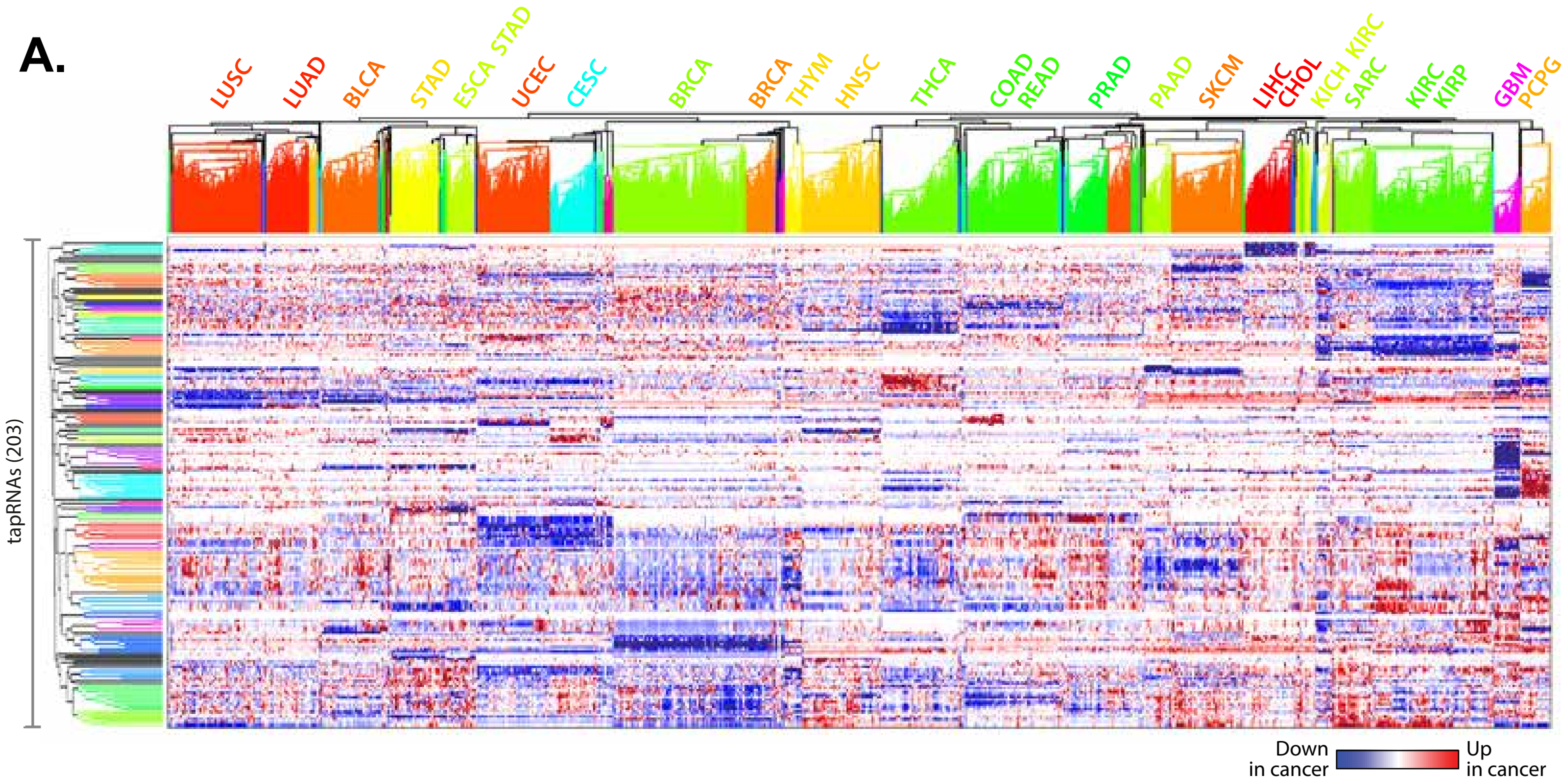
LUSC
LUAD
BLCA
STAD
ESCA STAD
UCEC
CESC
BRCA
BRCA
THYM
HNSC
THCA
COAD
READ
PRAD
PAAD
SKCM
LIHC
CHOL
KICH KIRC
SARC
KIRC
KIRP
GBM
PCPG
tapRNAs (203)
Down in cancer
Up in cancer

B.

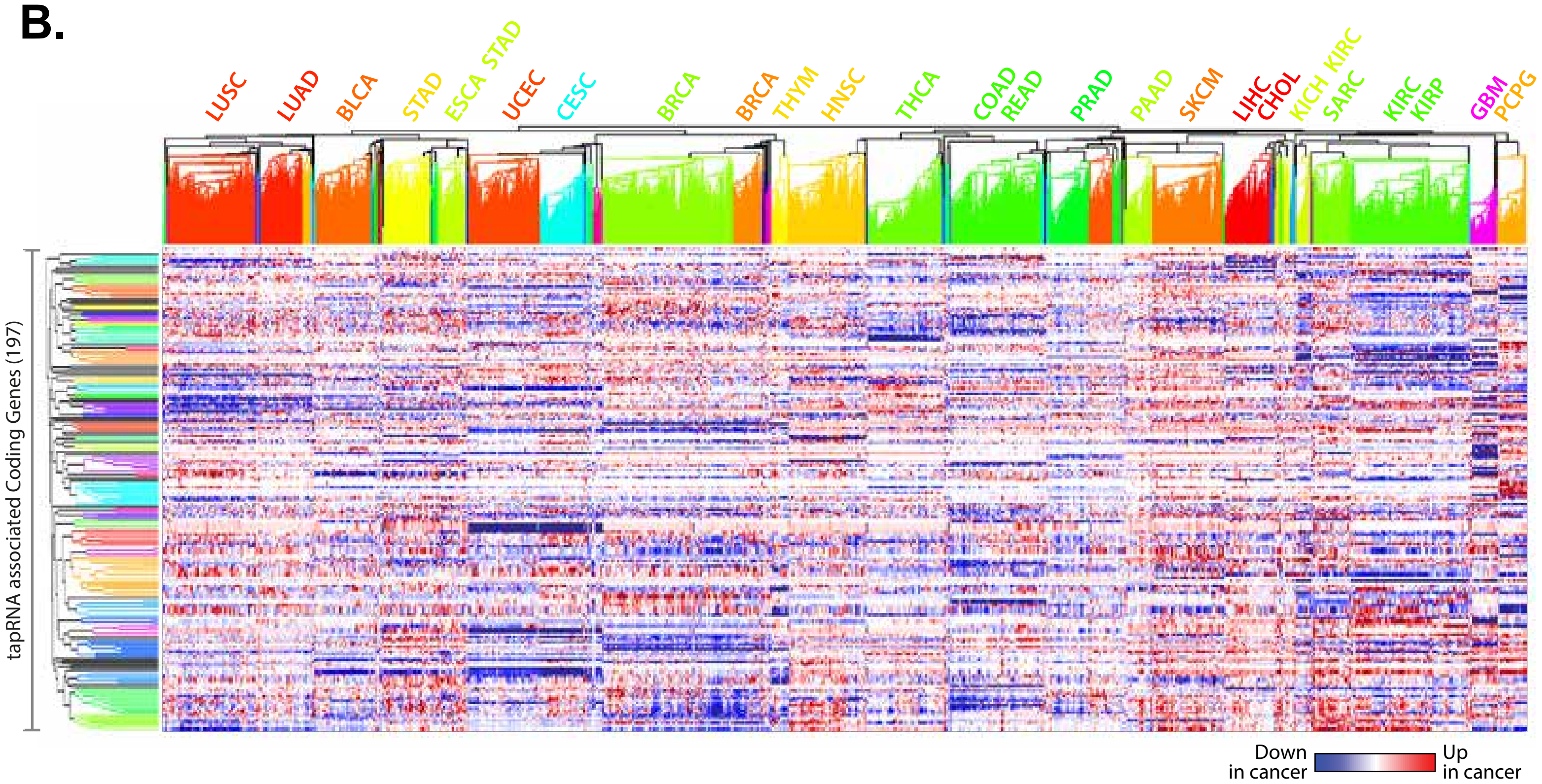
LUSC
LUAD
BLCA
STAD
ESCA STAD
UCEC
CESC
BRCA
BRCA
THYM
HNSC
THCA
COAD
READ
PRAD
PAAD
SKCM
LIHC
CHOL
KICH KIRC
SARC
KIRC
KIRP
GBM
PCPG
tapRNA associated Coding Genes (197)
Down in cancer
Up in cancer
